# Primer-BLAST: A tool to design target-specific primers for polymerase chain reaction

**DOI:** 10.1186/1471-2105-13-134

**Published:** 2012-06-18

**Authors:** Jian Ye, George Coulouris, Irena Zaretskaya, Ioana Cutcutache, Steve Rozen, Thomas L Madden

**Affiliations:** 1 National Center for Biotechnology Information, National Library of Medicine, National Institutes of Health, Building 45, 8600 Rockville Pike, Bethesda, MD, 20894, USA; 2 Neuroscience and Behavioral Disorders Program, Duke-NUS Graduate Medical School, 8 College Road, Singapore, 169857, Singapore

## Abstract

**Background:**

Choosing appropriate primers is probably the single most important factor affecting the polymerase chain reaction (PCR). Specific amplification of the intended target requires that primers do not have matches to other targets in certain orientations and within certain distances that allow undesired amplification. The process of designing specific primers typically involves two stages. First, the primers flanking regions of interest are generated either manually or using software tools; then they are searched against an appropriate nucleotide sequence database using tools such as BLAST to examine the potential targets. However, the latter is not an easy process as one needs to examine many details between primers and targets, such as the number and the positions of matched bases, the primer orientations and distance between forward and reverse primers. The complexity of such analysis usually makes this a time-consuming and very difficult task for users, especially when the primers have a large number of hits. Furthermore, although the BLAST program has been widely used for primer target detection, it is in fact not an ideal tool for this purpose as BLAST is a local alignment algorithm and does not necessarily return complete match information over the entire primer range.

**Results:**

We present a new software tool called Primer-BLAST to alleviate the difficulty in designing target-specific primers. This tool combines BLAST with a global alignment algorithm to ensure a full primer-target alignment and is sensitive enough to detect targets that have a significant number of mismatches to primers. Primer-BLAST allows users to design new target-specific primers in one step as well as to check the specificity of pre-existing primers. Primer-BLAST also supports placing primers based on exon/intron locations and excluding single nucleotide polymorphism (SNP) sites in primers.

**Conclusions:**

We describe a robust and fully implemented general purpose primer design tool that designs target-specific PCR primers. Primer-BLAST offers flexible options to adjust the specificity threshold and other primer properties. This tool is publicly available at http://www.ncbi.nlm.nih.gov/tools/primer-blast.

## Background

PCR is a commonly used method to amplify DNA of interest in many fields such as biomedical research, diagnostic testing and forensic testing. While the outcome of PCR can be influenced by many other conditions such as the template DNA preparation and reaction conditions, designing a good pair of primers is a critical factor. A general requirement is that primers should have similar melting temperatures (Tm) and a balanced G/C content, but should avoid self-complementarity and hair-pin structure. Additional requirements may also apply in certain cases. For example, to avoid unwanted amplification of genomic DNA in reverse transcription PCR (RT-PCR), it is recommended that a primer pair span an intron, or that one of the primers be located at an exon-exon junction. Another concern is the possible impact of SNPs in the primer regions. Since a SNP may act as a mismatch in some cases, one should consider picking primers outside of such regions.

One critical primer property is the target specificity. Ideally, a primer pair should only amplify the intended target, but not any unintended targets. This is especially important for real time quantitative PCR (qPCR) where in many cases the amount of PCR product is represented by the total intensity of fluorescence incorporated into amplified DNA and any amplification of unintended targets can affect the measurement [1]. Since different parts of chromosomes or transcripts may share some nucleotide similarity due to either homologous regions or fortuitous matches, it is not uncommon that a primer pair intended for one target will also bind to another one, resulting in non-specific target amplifications.

A number of studies have investigated the effects of mismatches between targets and primers and have shown that a target can be amplified even if it has a few mismatches to the primers [2-5]. In general, mismatches towards the 3’ end affect target amplification much more than mismatches towards the 5’ end. Although the results from these studies vary and the precise relationship between mismatches and amplification is difficult to establish, the consensus is that a two base mismatch at the 3’ end generally prevents amplification. A single base mismatch (even at the very 3’ end), as well as a few mismatches in the middle or toward the 5’ end, still allows amplification, though at a reduced efficiency for some cases. Given the variable effects of the mismatches and the likelihood that users may have different criteria based on their own experimental conditions, it is important that a software tool should offer the capability to detect up to a few mismatches over the entire primer range and the flexibility to change the specificity settings. In this regard, it is worth pointing out that the BLAST program [6] is in fact not an ideal tool for this purpose, as it uses a local alignment algorithm and does not necessarily return complete match information between the primer and target, particularly when the match is not perfect toward the primer ends.

A number of public software tools have been developed to aid the primer design process. Notably, the widely used Primer3 program [7] designs primers based on a variety of parameters. Since it does not perform target analysis, users typically need to test the primer specificity using additional tools. However, this process is time-consuming and sometimes even impractical if the primers have too many database matches (as with a BLAST search, for example).

Several software programs, such as In-Silico PCR [8] and Reverse ePCR [9], do not design primers but rather determine the amplification targets of user-supplied primer pairs. However, even with the help of these tools, finding specific primers can still be a difficult process, because users often need to go through many candidate primers manually. In addition, since these software tools mostly use an index-based strategy, which requires computationally intensive pre-processing of the search database, they are limited by the availability of databases and are usually not sensitive enough to detect targets that have a significant number of mismatches to primers yet are potentially amplifiable.

It is therefore desirable to combine various elements of primer design requirements into one process such that users can simply input the template and obtain the desired target-specific primers. There are several existing programs that have addressed some aspects of this issue. Autoprime [10] designs primers spanning exon junctions or introns so that the primers only target mRNA. However, it does not address the primer specificity issue. QuantPrime [11] is a specialized tool to design target-specific primers for detecting mRNA in real time PCR. Likewise, the PRIMEGENS Sequence Specific Primer Design tool [12] can also be used for specific primer design for a limited number of organisms. However, neither of these tools guarantees an accurate count of nucleotide matches between primer and target due to the fact that they both use a local alignment algorithm (i.e., BLAST) alone for the similarity search and thus may miss part of an alignment between primer and target [6]. Other limitations in these tools include low target detection sensitivity, limited specificity stringency options, no or limited support for designing primers based on exon/intron boundary requirements and limited coverage of organisms in search databases.

We have developed Primer-BLAST as a general purpose public tool that helps users design target-specific primers. Primer-BLAST offers flexibility to accommodate different primer design needs. Users can either design new primers or check the specificity of pre-existing primers. Notably, Primer-BLAST incorporates a global alignment mechanism and is designed to be very sensitive in detecting potential amplification targets. Finally, it has the capability to place primers based on exon/intron boundaries and SNP locations. We are not aware of any other general purpose public tool that has integrated similar functionality to design target-specific primers.

## Implementation

The Primer-BLAST program consists of a module for generating candidate primer pairs and a module for checking the target specificity of the generated primer pairs. Primer3 is used to generate the candidate primer pairs for a given template sequence. The specificity checking module uses BLAST along with the Needleman-Wunsch (NW) global alignment algorithm [13] to look for matches between the primers and targets. The Primer-BLAST program was implemented using the NCBI C++ toolkit and the Primer3 C programming interface, which recently added the capability to target primers to one or more specified regions as well as between specified bases (for splice site specification). The Primer-BLAST program is run on a farm of machines at the NCBI to provide faster and more reliable service to users.

In order to increase the chance of finding specific primer pairs, at least one primer (for a given primer pair) should be located in regions where the PCR template does not share high similarity to unintended targets if possible. To achieve this, the PCR template sequence is submitted to MegaBLAST [14] for a fast search to identify regions that are highly similar to unintended sequences in the user-specified database. Primer3 is then instructed to place at least one primer (for a given primer pair), if possible, outside of such regions. If the user-submitted template is a RefSeq accession or NCBI-gi, Primer-BLAST retrieves exon/intron boundaries as well as SNP locations associated with the template from the NCBI Entrez database in case of a requirement to place the primers based on exon/intron boundaries and SNP locations.

The candidate primer pairs are then subject to the specificity checking process. Since Primer3 generates many candidate primer pairs and all of them may need to undergo specificity checking to obtain the specified number of target-specific primer pairs, the entire search process can be very long if each pair is searched with BLAST individually. To solve this problem, we observe that any primer is essentially a sub-region of the PCR template and a single BLAST result using the template as a query should contain alignment information for all primer pairs. As a result, when a user supplies a template sequence to design new primers (the template case), the template itself is submitted for a BLAST search just once, which greatly reduces the total search time. For cases where users submit a pre-existing primer pair to perform specificity checking (the primer-only case), an artificial template sequence is generated for the BLAST search by connecting both primers with a 20 base spacer region of N’s. This ensures that each primer will be treated separately in the BLAST search and thus achieves the equivalent effect of performing a separate BLAST search for each primer. To further minimize the search time, all regions on the template that do not contain candidate primers are masked out to avoid irrelevant BLAST hits. Since all candidate primer locations on the template are established by Primer3 already, amplification targets (amplicons) for all primer pairs can be identified using the single BLAST result above. A primer pair is deemed to be specific only if it has no amplicons on any targets other than the submitted template within the specificity checking threshold specified by the user. Otherwise, it is considered non-specific. In addition to checking for amplicons between the forward and the reverse primers, Primer-BLAST also checks amplicons arising from either primer alone. For example, the forward primer could also act as a reverse primer if it happens to match some regions on the minus strand of the template.

The specificity checking module by default uses BLAST search parameters that ensure high sensitivity such that it can detect a target that contains up to 35% mismatches to the primer sequence. The default BLAST expect value cutoff is 30,000 for the primer-only case and it is typically adjusted much higher for the template case (see below). This expect value is 3000 times higher than the standard BLAST program default (the higher the expect value cutoff, the more sensitive the search) and is necessary to ensure detection of targets that have a significant number of mismatches to primers yet are potentially amplifiable in PCR. Other highly sensitive default parameters include a word size of seven (standard BLAST uses 11), 50,000 for the maximum number of database sequences (standard BLAST uses 250) and 1 for match reward to mismatch penalty ratio (standard BLAST uses 1.5). The expect value for a given BLAST match between a primer and a target is roughly proportional to the query sequence length given the same search database [6], but the query lengths used in the primer-only case and the template case are often very different. Therefore, there can be a large discrepancy in the expect values between the BLAST matches in the two cases, even though the same primer sequences are being aligned. To resolve this issue, we internally adjust the specified expect value cutoff for the template case using the length of the artificial template from the primer-only case as a guide. This ensures that the BLAST results are equivalent between submitting a template and submitting primers only.

Since a complete alignment between a primer and its targets is desired for accurate specificity checking, the NW global alignment algorithm [13] is incorporated into Primer-BLAST to realign any regions that are not fully aligned by BLAST.

## Results and discussion

### User interface

**Figure 1:**
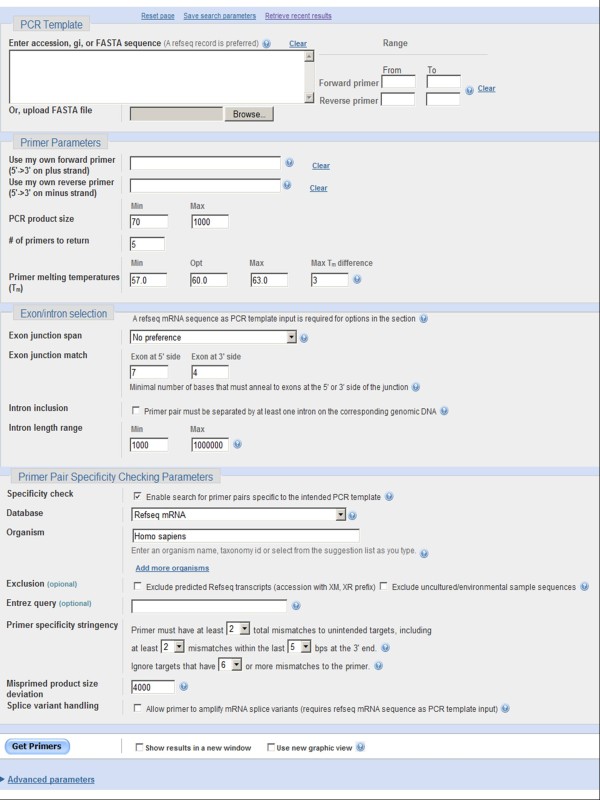
The Primer-BLAST web interface.

The interface consists of several sections where users can input the PCR template and/or pre-existing primers as well as other user-adjustable parameters (Figure 1).

Users can design new primer pairs by entering the DNA template alone or they can design one primer by entering the template plus the other pre-existing primer. Primer-BLAST can check the specificity of pre-existing primers with or without the template. The PCR template can be a raw DNA sequence in FASTA format or an NCBI accession. If available, a RefSeq accession is recommended as it carries more information about the sequence [15], which allows Primer-BLAST to better identify the template and thus perform better primer specificity checking. Primer-BLAST also performs a fast check on any raw sequence input to determine if it is an exact match to a RefSeq sequence, in which case Primer-BLAST will use the RefSeq accession as the template. The template length is limited to 50,000 bases. For longer templates, the primer range (upper right corner of Figure 1) should be used to limit the length.

Primer-BLAST also offers the capability to design primers based on exon/intron structure so that PCR amplification can better be targeted to mRNA. Users can specify whether a primer should span an exon/exon junction with an adjustable number of bases on each side of the junction and whether the primer pair should span an intron along with an option to specify intron size. As this option depends on accurate exon/exon boundary annotation, a RefSeq accession (as PCR template) is required since RefSeq represents the best curated sequence category at NCBI.

Several database options are available for specificity checking with broad organism coverage. They include the RefSeq mRNA database and RefSeq genome database, which, as of Nov 18, 2011, contain 226 and 7,546 organisms, respectively. These databases are non-redundant as they don’t have the same sequence regions covered more than once, thus allowing better specificity checking. They are the databases of choice for designing new target-specific primers. The traditional nr database, containing redundant entries, is also available and is mostly recommended for organisms that are not covered by other databases or for sequence entries not covered by the RefSeq databases.

Primer-BLAST offers flexible specificity stringency options. Users can specify the number of mismatches that a primer pair must have to unintended targets as well as a 3’ end region where these mismatches must be present. In addition, users can specify the mismatch threshold above which any targets should be ignored (i.e., filtering out targets having too many mismatches to be a concern for non-specific amplification). The default specificity settings are that at least one primer (for a given primer pair) must have two or more mismatches to unintended targets in the last five bases at the 3’ end, and that any targets with six mismatches or more to at least one primer (for a given primer pair) should be ignored.

It is not always possible to generate primers specific to a particular splice variant mRNA when the difference in exons is not sufficient to distinguish one from the rest. Therefore, Primer-BLAST offers the splice variant handling option that allows amplification of other variants from the same gene.

Other options, including parameters for BLAST search sensitivity, SNP exclusion and primer properties, etc. can be found under the “advanced parameters” section.

### Presentation of results

The results page reports the specificity of the generated primers, a graphic summary of primer pairs in relation to the PCR template and certain features such as exons, as well as detailed information on each primer pair. It will only show target-specific primers if found; otherwise, it will report all primers. In all cases, the actual targets will be listed along with detailed alignments between primers and targets.

**Figure 2:**
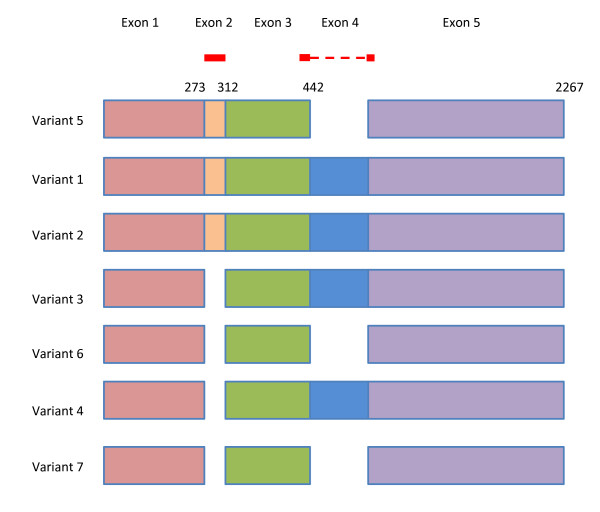
**Schematic alignment of mRNA transcript variants from the ZNF419 gene.** Numbers indicate the end positions of exons for variant 5. The red lines indicate the primer regions picked by Primer-BLAST. Note that several transcripts differ by 3 nucleotides due to use of slightly different splice sites even though they share same exons (i.e., variant 2 v.s. variant 1, variant 7 v.s. variant 6 and variant 4 v.s. variant 3). The graph is adapted from NCBI gene report (http://www.ncbi.nlm.nih.gov/sites/entrez?db=gene&cmd=Retrieve&dopt=full_report&list_uids=79744. Data accessed 11/02/2011).

**Figure 3:**
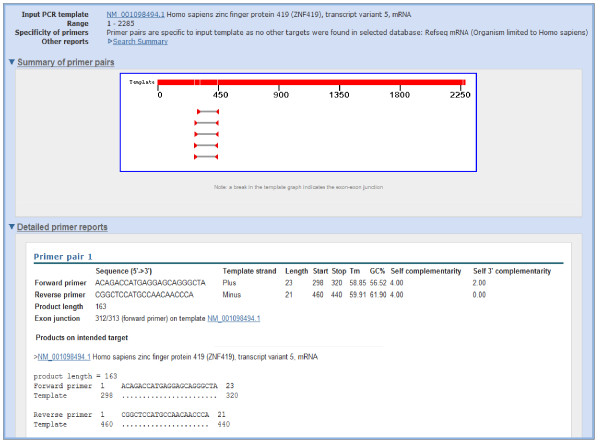
**Example results of designing target-specific primers.** Note that while five primer pairs were returned (as shown in graphic summary), due to space limitation, the figure shows details for the first primer pair only. The “Search Summary” link, when clicked, shows the search parameters used as well as the total number of BLAST hits that are generated during the search process (355,744 hits for the current search). Numbers in alignments indicate the start and end positions for primer and target. A dot (.) indicates nucleotide identity to primer sequence. The search was done on 11/02/2011.

As an example to illustrate the functionality of Primer-BLAST, we design primers using the human zinc finger protein 419 (ZNF419) transcript variant 5 mRNA (Genbank accession NM_001098494). As shown in Figure 2, there are seven transcript variants for the ZNF419 gene according to the NCBI Gene report. The search used default values that require at least one primer (for a given primer pair) to have two or more mismatches to unintended targets in the last five bases at the 3’ end. The specificity checking was performed against the NCBI RefSeq mRNA database with organism limited to human, since the goal was to find primer pairs that are specific to this transcript only among the human transcriptome. To avoid possible genomic DNA amplification, the option “Primer must span an exon-exon junction” is selected. As shown in Figure 3, Primer-BLAST successfully returned five specific primer pairs and the detailed alignment between targets and primers are shown. During the search process, Primer-BLAST examined a total of 355,744 BLAST matches (see Figure 3 legend) that represent not only the transcript variants of this gene but also a large number of transcripts from other genes that show matches of varying degrees to the candidate primers. This underscores the challenge if the same thorough examination of primer specificity task were to be performed manually. The average search time for designing new primers with default parameters using a human mRNA template of average length (2800-3000 bases) is 2.6 minutes.

Examination of the alignment of transcript variant 5 with other variants indicates that the existence of exon 2 and absence of exon 4, when combined, are the only features that distinguish it from the rest (Figure 2). Not surprisingly, part or all of the forward primers picked by Primer-BLAST are located in exon 2 and all reverse primers are on the junctions between exon 3 and 5 (since exon 4 is not present).

**Figure 4:**
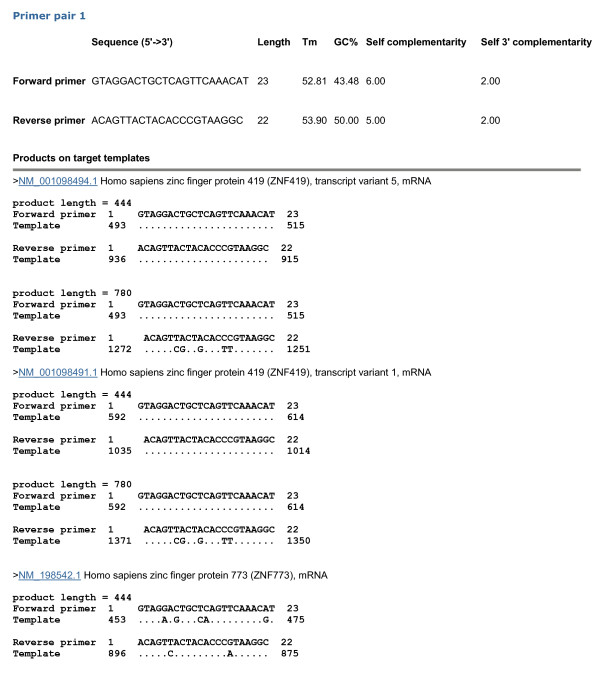
**Specificity checking of pre-existing primers.** This search was performed by entering the forward and reverse primers without entering any template. The primers (forward primer: GTAGGACTGCTCAGTTCAAACAT, reverse primer: ACAGTTACTACACCCGTAAGGC) were obtained from PrimerBank (http://pga.mgh.harvard.edu/primerbank/) on 11/02/2011 using ZNF419 transcript variant 5 (GenBank accession NM_001098494). While the results indicated all 7 transcript variants from the ZNF419 gene have the same amplicons, this figure shows the details only for variants 1 and 5 due to space limitation. The current search generated 11,236 BLAST hits (done on 11/02/2011).

Primer-BLAST can also be used to check the specificity of pre-existing primers. As an example, we obtained the primers for the same PCR template as above (i.e., ZNF419 transcript variant 5 mRNA) from PrimerBank, which deposits many pre-computed gene-specific primers for detecting mRNA [16]. Again, the default specificity parameters were used and the result is presented in Figure 4. The search generated 11,236 BLAST hits from the RefSeq mRNA database with organism limited to human, which again illustrates the difficulty of manually examining the BLAST results, even for a single primer pair. This primer pair indeed shows perfect matches to the ZNF419 gene transcript variant 5 as well as other transcript variants from the same gene and would generate a 444 base amplicon. This is consistent with PrimerBank’s selection criteria that the primers are specific only at the gene level, not at the transcript level. Interestingly, some other potential amplicons are also detected. One is an additional 780 base amplicon present in the intended ZNF419 transcript. The other is the 444 base amplicon from a different gene (i.e., human zinc finger protein 773, Genbank accession NM_198542.1). However, there are up to 5 mismatches between at least one of the primers and the targets, which is probably sufficient to prevent amplification interference or non-specific amplification. Nevertheless, users can scrutinize this result and make judgment based on their own experimental experiences.

### Comparison to other primer design tools

Primer-BLAST offers a number of features that are not available in other software tools. Table 1 gives a brief summary of these features, many of which are important for various primer design requirements and allows examination of primer specificity details by users. For example, Primer-BLAST is the only tool that offers the ability to specify the number of mismatches that a specific primer pair must have to unintended targets and a custom 3’ end region where certain number of mismatches must be present. This option is important to meet different primer specificity stringency requirements by users, since the specificity of a primer is typically judged by the number of mismatches it has to unintended targets (higher number of mismatches offer more specificity) and the locations of such mismatches (mismatches closer to 3’ end offer more specificity). Primer-BLAST is the only program among the three that will place primers on different exons (i.e., to span an intron) to avoid amplification of genomic DNA and also the only one to allow customization of the number of nucleotide matches on either side of an exon/exon junction. Furthermore, Primer-BLAST presents detailed alignments between the primers and targets found.

**Table 1:** Comparison of selected features among different primer design tools

	**Primer-BLAST**	**QuantPrime**	**PRIMEGENS**
**General**			
Scope of primer design task	General purpose	Real time PCR only	General purpose
Alignment algorithm for specificity checking	Local and global	Local only	Local only
**Specificity checking options**			
Specify a range for the number of nucleotide mismatches required between primer and unintended targets	Yes	No	No
Define a custom region at 3’ end where certain number of nucleotide mismatches must exist between primer and unintended target	Yes	No	No
Number of organisms covered by mRNA and genome databases	7546	333	18
**Exon/intron requirement settings**			
Place primers across exon/exon junction	Yes	Yes	No
Place primer pairs that span an intron	Yes	No	No
Set custom nucleotide match on either side of exon/exon junction	Yes	No	No
**Other primer design options**			
Allow custom PCR template sequence in FASTA format	Yes	No	Yes
Avoid SNP in primers	Yes	Yes	No
Allow specificity checking for pre-existing primers	Yes	No	No
**Result presentation**			
Graphic overview of primers found	Yes	No	No
Detailed nucleotide alignments between primers and targets	Yes	No	No

Another advantage of Primer-BLAST is its high detection sensitivity. As shown above, Primer-BLAST is, by default, capable of detecting potential amplification targets that have up to 5 mismatches to a primer. Primer-Blast achieves this result by using highly sensitive BLAST parameters as well as an additional NW global alignment algorithm to ensure a complete alignment between the primer and its target. However, one caveat is that the BLAST algorithm [6] requires a minimum stretch of nucleotide matches (word size) between the query and target and any tools using BLAST as search algorithm are subject to this limitation. Consequently, Primer-BLAST (with default parameters) will miss any targets that have 6 or fewer consecutive matches to a primer (since Primer-BLAST uses a word size of 7 by default). For example, if a target has mismatches to a primer of 20 bases at positions 7 and 14 (assuming the 5’ end is position one), the target will be missed by Primer-BLAST (with default parameters) even though it has only 2 mismatches. Assuming a random distribution of mismatch locations, it is possible to calculate the number of possible arrangements of 18 matches and 2 mismatches. There are 20*19 different ways to place the 2 mismatches among the 18 matches, but only 2 of these result in a word size shorter than 7, so the probability of missing a target with 2 mismatches to a primer of 20 bases is 2/(20*19) or about 0.5%.

We next compare the target detection sensitivity between Primer-BLAST and other primer design tools such as QuantPrime and PRIMEGENS. Ideally, a comparison of detection sensitivity would be to directly test the specificity checking modules across all tools using primers generated from a third party (for example, primers generated by Primer3). Unfortunately, this option is not available as Primer-BLAST is the only tool that offers specificity checking directly (i.e., specificity checking of pre-existing primers). As an alternative, we used QuantPrime and PRIMEGENS to design target-specific primers and then used Primer-BLAST to examine these primers for potential targets. If Primer-BLAST does not find targets other than the intended one (i.e., the input mRNA template itself), then one can conclude that QuantPrime and PRIMEGENS are at least as sensitive as Primer-BLAST. On the other hand, existence of unintended targets revealed by Primer-BLAST would indicate that these tools are not as sensitive as Primer-BLAST in target detection sensitivity (since these tools had already examined such targets but were not able to avoid them during their selection processes for target-specific primers).

The test templates are randomly selected from NCBI Refseq mRNA database and they include 52 human sequences for testing QuantPrime and 24 Arabidopsis thaliana sequences for testing PRIMEGENS (since PRIMEGENS does not support human sequences). As shown in Table 2, QuantPrime or PRIMEGENS generated primer pairs for most test cases that they deemed to be specific for input templates. However, Primer-BLAST revealed that many of these (13.4% of primer pairs from QuantPrime and 43.3% of primers pairs from PRIMEGENS) have potential unintended targets that show between one and five nucleotide mismatches. As a result, a large portion of test cases have at least one primer pair that has potential unintended targets (31.5% for QuantPrime and 93.3% for PRIMEGENS). Some targets have only one or two mismatches to primers generated from QuantPrime (18.5%) although this portion is much smaller for PRIMEGENS (3.4%).

**Table 2:** **Summary of potential unintended targets for primer pairs reported by QuantPrime and PRIMEGENES**^**a**^

	QuantPrime	PRIMEGENS ^b^
Total number of test cases	52	24
Number of cases where QuantPrime or PRIMEGENS was able to generate primers	38	15
Number of cases with potential unintended targets	12	14
Percentage of cases having at least one primer pair with potential unintended target	31.5% (12/38)	93.3% (14/15)
Number of total primer pairs generated	373	138
Number of primer pairs with potential unintended targets	50	60
Percentage of primer pairs with potential unintended targets	13.4% (50/373)	43.3% (60/138)
Number of potential unintended targets	162	116
Number of potential unintended targets with one or two mismatches to forward or reverse primer	30	4
Percentage of potential unintended targets with one or two mismatches to forward or reverse primer	18.5% (30/162)	3.4% (4/116)

^a^: Primer pairs generated by QuantPrime or PRIMEGENES from randomly selected templates (see Additional files 1 and 2 for details) were fed into Primer-BLAST for target specificity checking using the matching organism (i.e., Human for QuantPrime and Arabidopsis thaliana for PRIMEGENS) and matching database (RefSeq mRNA database for all cases). Default search parameters were used for Primer-BLAST searches which can detect targets with up to 5 mismatches to primers. Since QuantPrime and PRIMEGENES still use databases that are a few years old and Primer-BLAST uses the regularly updated NCBI database, we only counted those unintended targets found by Primer-BLAST that are also present in QuantPrime database (RefSeq 04/30/09 (reference assembly)(genome+) (splice variants)) or PRIMEGENS database (Arabidopsis TAIR9 cDNA) . The searches were performed between 2/3/2012 and 2/6/2012

^b:^ Since PRIMEGENS adds non-specific primer pairs (i.e., pairs having more than one hybridization targets, see Additional file 2) to the primer result if it does not find sufficient number of target-specific primer pairs (i.e., pairs having only one hybridization target), we excluded all non-specific primer pairs in our analysis. In essence, our analysis only includes primer pairs that are deemed to be specific for the test template sequences by PRIMEGENS.

**Figure 5:**
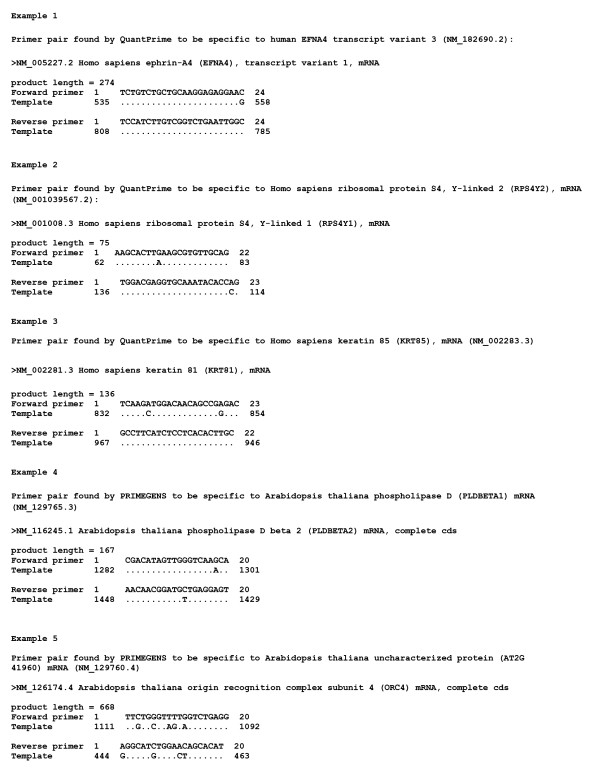
**Examples of potential unintended targets for primer pairs generated by QuantPrime and PRIMEGENS.** Example targets are extracted from Primer-BLAST specificity checking results for primer pairs generated by QuantPrime or PRIMEGENS (a total of 162 and 116 potential unintended targets were identified for QuantPrime and PRIMEGENS, respectively. See Table 2 for details). The example primers correspond to those underlined in Additional files 1 and 2.

Figure 5 shows details for 5 potential unintended targets. For example, QuantPrime generates two primer pairs (example 1 and 2) that are designed to be specific for Genbank accessions NM_182690.2 and NM_001039567.2, respectively (Figure 5). However, Primer-BLAST reveals that these two pairs have potential unintended targets, NM_005227.2 and NM_001008.3, respectively, that have only a single nucleotide mismatch to the forward or the reverse primers. A primer pair generated by PRIMEGENS (example 4) also shows potential unintended target with only a single mismatch. As reviewed earlier, a single nucleotide mismatch (even at the 3’ end) does not significantly affect target amplification and therefore these primer pairs are not likely to be specific to their intended targets. The failure to detect a single base mismatch at (the nucleotide base G in example 1) or near the 3’ end (the nucleotide base C in example 2) shows the drawback of using only a local alignment algorithm. A local alignment attempts to maximize the score it returns, so it will not include mismatches at or (possibly) near the end of an alignment as they would decrease the overall score [6]. Other cases of unintended targets include 2 mismatches (example 3), or 5 mismatches (example 5) to one of the primers.

Thus we conclude that Primer-BLAST is able to detect potential unintended targets that are missed by QuantPrime or PRIMERGENS during the specificity screening process.

## Conclusions

Primer-BLAST is a general purpose target-specific PCR primer design tool that offers a level of sensitivity and usability not found in other tools. It offers flexible options to meet various specificity stringency requirements and uses a BLAST search (with a local alignment) to quickly identify as many targets as possible, but follows that up with a global alignment to get an accurate count of mismatches over the entire primer if BLAST alignments only cover part of the primer length. Primer-BLAST is run on a farm of machines at the NCBI to provide better service for users. Input can be a Genbank accession, a FASTA file, or even primers from another source. It also takes advantage of rich information from NCBI sequence databases to support other requirements such as placing primers based on intron/exon boundaries and SNP locations. Finally, it displays alignments between primers and targets found, allowing the user to make a decision on whether or not to use the primer pairs when potentially unintended targets exist.

## Availability and requirements

**Project name:** Primer-BLAST

**Project home page:**http://www.ncbi.nlm.nih.gov/tools/primer-blast

**Operating system(s):** Platform independent

**Programming language:** C++

**Other requirements:** Web browser

**License:** Primer-BLAST web tool is freely available for all users. The source code for the blast search and primer specificity checking are in public domain [17] and are available in NCBI C++ toolkit. Primer3 is freely available in open source form under the GNU General Public License v2.

**Any restrictions to use by non-academics:** None

## Competing interests

The authors declare that they have no competing interests.

## Authors’ contributions

All authors participated in the design and coding of the software. JY drafted the manuscript and all authors read and approved the final version of the manuscript.

## Supplementary Material

Additional file 1 PrimerPairsFromQuantPrime.doc Primer pairs generated from QuantPrime. Fifty-two randomly selected human template sequences from NCBI Refseq mRNA database are used to generate target-specific primers by QuantPrime. The “SYBR Green real-time qPCR (no splice variant hits)” option was selected with the organism set to human and the database set to “RefSeq 04/30/09 (reference assembly)(genome+) (splice variants)”. Default values were used for all other options. The underlined pairs are used as example cases in Figure 5. Click here for file

Additional file 2 PrimerPairsFromPRIMEGENS.doc Primer pairs generated from PRIMEGENS. Twenty-four randomly selected template sequences from NCBI Refseq mRNA database are used to generate target-specific primers by PRIMEGENS (Arabidopsis thaliana sequences were chosen since PRIMEGENS does not support transcript database for human). The database “Arabidopsis TAIR9 cDNA” was selected. Default values were used for all other options. The underlined pairs are used as example cases in Figure 5. Click here for file
