# Supplementary material for: Primer-BLAST: A tool to design target-specific primers for polymerase chain reaction
Source: BMC Bioinformatics. 2012 Jun 18;13:134. doi: 10.1186/1471-2105-13-134 (PMC3412702; doi:10.1186/1471-2105-13-134)
Supplement: Additional file 1 — PrimerPairsFromQuantPrime.doc Primer pairs generated from QuantPrime. Fifty-two randomly selected human template sequences from NCBI Refseq mRNA database are used to generate target-specific primers by QuantPrime. The “SYBR Green real-time qPCR (no splice variant hits)” option was selected with the organism set to human and the database set to “RefSeq 04/30/09 (reference assembly)(genome+) (splice variants)”. Default values were used for all other options. The underlined pairs are used as example cases in Figure 5. [file 1471-2105-13-134-S1.doc]

**NM_000843.3**

**CTCCATCGACGGATTTGACCAG CCAGATGTTCCTGCGGTTGTTC 67 Yes 4.2249**

**AAAGGGCCTCCATCGACGGATTTG ACCAGATGTTCCTGCGGTTGTTC 75 Yes 4.8242**

**CTCCATCGACGGATTTGACCAG AGATGTTCCTGCGGTTGTTCTCC 65 Yes 4.2473**

**CTCCATCGACGGATTTGACCAG TGTTCCTGCGGTTGTTCTCCAG 62 Yes 3.225**

**CTCCATCGACGGATTTGACCAG AGATGTTCCTGCGGTTGTTCTC 65 Yes 5.3659**

**CTCCATCGACGGATTTGACCAG CAGATGTTCCTGCGGTTGTTCTC 66 Yes 5.2841**

**TGATCAGGAGACTCATGGAGACG CGCCTGATGTCATCCTCATTGG 73 Yes 5.6555**

**CTCCATCGACGGATTTGACCAG GATGTTCCTGCGGTTGTTCTCC 64 Yes 4.4794**

**GTGATCAGGAGACTCATGGAGACG GCCTGATGTCATCCTCATTGGC 73 Yes 5.3593**

**TGATCAGGAGACTCATGGAGACG GCCTGATGTCATCCTCATTGGC 72 Yes 5.3875**

**NM_000920.3**

**(no primer pairs matching criteria)**

**NM_001039567.2**

**TTCTGTTCCGTCGCGGGATTTC TGGACGAGGTGCAAATACACCAG 118 Yes 1.902**

**ACAGTGGAAGAGGCAAAGTACAAG CACCAGGTGTGGAATTCCCTTTG 78 Yes 7.4209**

**ACAGTGGAAGAGGCAAAGTACAAG ACCAGGTGTGGAATTCCCTTTG 77 Yes 7.5341**

**ACAGTGGAAGAGGCAAAGTACAAG CAGGTGTGGAATTCCCTTTGTCC 75 Yes 7.67**

**ACAGTGGAAGAGGCAAAGTACAAG CCAGGTGTGGAATTCCCTTTGTC 76 Yes 7.67**

**AAGCACTTGAAGCGTGTTGCAG TGGACGAGGTGCAAATACACCAG 75 Yes 2.6128**

**AAGCACTTGAAGCGTGTTGCAG GGACGAGGTGCAAATACACCAG 74 Yes 3.092**

**AGAAGCACTTGAAGCGTGTTGC TGGACGAGGTGCAAATACACCAG 77 Yes 2.8646**

**AGAAGCACTTGAAGCGTGTTGC GGACGAGGTGCAAATACACCAG 76 Yes 3.3438**

**CAAGAAGCACTTGAAGCGTGTTGC TGGACGAGGTGCAAATACACCAG 79 Yes 3.7921**

**NM_001040647.1**

**(no primer pairs matching criteria)**

**NM_001040702.1**

**(no primer pairs matching criteria)**

**NM_001040876.1**

**(no primer pairs matching criteria)**

**NM_001042361.3**

**(no primer pairs matching criteria)**

**NM_001042393.1**

**(no primer pairs matching criteria)**

**NM_001042404.1**

**(no primer pairs matching criteria)**

**NM_001042410.1**

**CGTTTGGTGCGTCTTCTAAGGG TATTTAGCAGCAGCTCCCATGC 80 Yes 4.4712**

**ACGTTTGGTGCGTCTTCTAAGGG TTATTTAGCAGCAGCTCCCATGC 82 Yes 4.5251**

**CGTTTGGTGCGTCTTCTAAGGG TTATTTAGCAGCAGCTCCCATGC 81 Yes 4.9868**

**ACGTTTGGTGCGTCTTCTAAGG TATTTAGCAGCAGCTCCCATGC 81 Yes 5.0967**

**ACGTTTGGTGCGTCTTCTAAGGG ATTTAGCAGCAGCTCCCATGC 80 Yes 4.7218**

**CGTTTGGTGCGTCTTCTAAGGG ATTTAGCAGCAGCTCCCATGC 79 Yes 5.1835**

**ACGTTTGGTGCGTCTTCTAAGG ATTTAGCAGCAGCTCCCATGC 80 Yes 5.809**

**CGTTTGGTGCGTCTTCTAAGGG TTTAGCAGCAGCTCCCATGC 78 Yes 6.514**

**ACGTTTGGTGCGTCTTCTAAGG TTTAGCAGCAGCTCCCATGC 79 Yes 7.1395**

**GACGTTTGGTGCGTCTTCTAAGG TTTAGCAGCAGCTCCCATGC 80 Yes 7.3229**

**NM_001042459.1**

**(no primer pairs matching criteria)**

**NM_001042466.1**

**AGGCCTTGGTGGAACATGTCAAG CTTGGGTTGCTGATCCATGTGC 134 Yes 2.7192**

**AGGCCTTGGTGGAACATGTCAAG TCCTTGGGTTGCTGATCCATGTG 136 Yes 3.041**

**AGGCCTTGGTGGAACATGTCAAG CCTTGGGTTGCTGATCCATGTG 135 Yes 3.2832**

**AGGCCTTGGTGGAACATGTCAAG CTCCTTGGGTTGCTGATCCATGTG 137 Yes 3.5232**

**GGTGGAACATGTCAAGGAGGAGTG CTTGGGTTGCTGATCCATGTGC 127 Yes 3.7923**

**GGTGGAACATGTCAAGGAGGAGTG TCCTTGGGTTGCTGATCCATGTG 129 Yes 4.114**

**GGTGGAACATGTCAAGGAGGAGTG CCTTGGGTTGCTGATCCATGTG 128 Yes 4.3562**

**AGGCCTTGGTGGAACATGTCAAG TTGGGTTGCTGATCCATGTGC 133 Yes 4.5696**

**GGTGGAACATGTCAAGGAGGAGTG CTCCTTGGGTTGCTGATCCATGTG 130 Yes 4.5962**

**GGAACATGTCAAGGAGGAGTGTG CTTGGGTTGCTGATCCATGTGC 124 Yes 4.82**

**NM_001042478.1**

**TTCTGGCCTCAGACCACCTTTG TGGAGCATAAAGGCCAGCTTCAC 72 No 2.4955**

**TTCTGGCCTCAGACCACCTTTG GGAGCATAAAGGCCAGCTTCAC 71 No 2.9757**

**TTCCAACACGTTCTGGCCTCAG TAAAGGCCAGCTTCACTTTGCC 75 No 2.6125**

**TTCCAACACGTTCTGGCCTCAG GCATAAAGGCCAGCTTCACTTTGC 78 No 2.8376**

**TCTGTTTCACGGCGGTTGTC AGGCCAGAACGTGTTGGAATC 96 Yes 8.2606**

**TCTGTTTCACGGCGGTTGTC GGCCAGAACGTGTTGGAATCTATC 95 Yes 8.4008**

**TCTGTTTCACGGCGGTTGTC CTTCACTTTGCCCAGAGCAGTC 142 Yes 6.441**

**TCTGTTTCACGGCGGTTGTC TTCACTTTGCCCAGAGCAGTC 141 Yes 8.3271**

**TCTGTTTCACGGCGGTTGTC AGCTTCACTTTGCCCAGAGC 144 Yes 9.251**

**TCTGTTTCACGGCGGTTGTC AAGGCCAGCTTCACTTTGCC 150 Yes 8.6755**

**NM_001042497.1**

**AAAGCGTGCCTGAAACAAGCC AGCTTTCTTATGTCCGTCGTCCTC 146 Yes+ 5.2866**

**AAAGCGTGCCTGAAACAAGCC GCTTTCTTATGTCCGTCGTCCTC 145 Yes+ 5.4721**

**GAAAGCGTGCCTGAAACAAGC AGCTTTCTTATGTCCGTCGTCCTC 147 Yes+ 6.6531**

**GAAAGCGTGCCTGAAACAAGC GCTTTCTTATGTCCGTCGTCCTC 146 Yes+ 6.8386**

**AACAAGCCGGAGTGAGCCTATG AGCTTTCTTATGTCCGTCGTCCTC 133 Yes+ 4.1936**

**AACAAGCCGGAGTGAGCCTATG GCTTTCTTATGTCCGTCGTCCTC 132 Yes+ 4.3791**

**AAACAAGCCGGAGTGAGCCTATG AGCTTTCTTATGTCCGTCGTCCTC 134 Yes+ 4.7768**

**AAACAAGCCGGAGTGAGCCTATG GCTTTCTTATGTCCGTCGTCCTC 133 Yes+ 4.9623**

**GAAACAAGCCGGAGTGAGCCTATG AGCTTTCTTATGTCCGTCGTCCTC 135 Yes+ 5.2882**

**CGGAGTGAGCCTATGAGTGAGATG AGCTTTCTTATGTCCGTCGTCCTC 126 Yes+ 6.4916**

**NM_001042531.1**

**GTTGCGGTTTCTCCGTTAGTGC TGGGCGAGATTCTGGATGAAACC 102 Yes 2.9272**

**GTTGCGGTTTCTCCGTTAGTGC GGTGGGCGAGATTCTGGATGAAAC 104 Yes 3.2278**

**GTTGCGGTTTCTCCGTTAGTGC GTGGGCGAGATTCTGGATGAAACC 103 Yes 3.2278**

**GTTGCGGTTTCTCCGTTAGTGC GGGCGAGATTCTGGATGAAACC 101 Yes 3.4253**

**GTTGCGGTTTCTCCGTTAGTGC GGGCGAGATTCTGGATGAAACCTC 101 Yes 3.4451**

**CGTTTGACCTTCCTCCGTTTCC TGGGCGAGATTCTGGATGAAACC 134 Yes 3.7772**

**GTTGCGGTTTCTCCGTTAGTGC GTGGGCGAGATTCTGGATGAAAC 103 Yes 3.9661**

**GTTGCGGTTTCTCCGTTAGTGC TGGGCGAGATTCTGGATGAAAC 102 Yes 4.0662**

**CTCCGTTTCCGTAGTTCCGAGTTG TGGGCGAGATTCTGGATGAAACC 122 Yes 4.0665**

**CGTTTGACCTTCCTCCGTTTCC GGGCGAGATTCTGGATGAAACC 133 Yes 4.2753**

**NM_001042536.1**

**(no primer pairs matching criteria)**

**NM_001042540.1**

**(no primer pairs matching criteria)**

**NM_001042548.1**

**TCAGCATGGAAGGGATCTCAAATG TCCACCTGAATTTCCAAAGGTGTG 68 No 8.8668**

**TCAGCATGGAAGGGATCTCAAATG TCTCCACCTGAATTTCCAAAGGTG 70 No 9.3477**

**ATGGCAGCTCTCAGAATGGGAAG TCCACCTGAATTTCCAAAGGTGTG 122 No 6.2153**

**ATGGCAGCTCTCAGAATGGGAAG TCTCCACCTGAATTTCCAAAGGTG 124 No 6.6962**

**TGGCAGCTCTCAGAATGGGAAG TCCACCTGAATTTCCAAAGGTGTG 121 No 5.4695**

**TGGCAGCTCTCAGAATGGGAAG TCTCCACCTGAATTTCCAAAGGTG 123 No 5.9504**

**AGCATGGAAGGGATCTCAAATGTC TCCACCTGAATTTCCAAAGGTGTG 66 No 8.8662**

**AGCATGGAAGGGATCTCAAATGTC TCTCCACCTGAATTTCCAAAGGTG 68 No 9.3471**

**ACACGCTTCAGCATGGAAGG TCCACCTGAATTTCCAAAGGTGTG 75 Yes 8.8528**

**ACACGCTTCAGCATGGAAGG TCTCCACCTGAATTTCCAAAGGTG 77 Yes 9.3337**

**NM_001763.2**

**ATCGTCCTCTACTGGGAGCATCAC AGGCACTATCACCGCCAAGATG 63 Yes 3.0049**

**ATCGTCCTCTACTGGGAGCATCAC AAGGCACTATCACCGCCAAGATG 64 Yes 3.5909**

**ATCGTCCTCTACTGGGAGCATCAC AAAGGCACTATCACCGCCAAGATG 65 Yes 4.2108**

**TCATCATCTTGGCGGTGATAGTGC ACAGCGTTTCCTGAACCAAAGC 71 Yes 4.0725**

**TCTTGGCGGTGATAGTGCCTTTAC ACAGCGTTTCCTGAACCAAAGC 65 Yes 4.1827**

**TTGGCGGTGATAGTGCCTTTAC ACAGCGTTTCCTGAACCAAAGC 63 Yes 4.199**

**CATCATCTTGGCGGTGATAGTGC ACAGCGTTTCCTGAACCAAAGC 70 Yes 4.2418**

**ATCATCTTGGCGGTGATAGTGC ACAGCGTTTCCTGAACCAAAGC 69 Yes 4.3287**

**CTTGGCGGTGATAGTGCCTTTAC ACAGCGTTTCCTGAACCAAAGC 64 Yes 4.3587**

**TCATCATCTTGGCGGTGATAGTGC ACAGCGTTTCCTGAACCAAAGCG 71 Yes 4.3875**

**NM_002283.3**

**AGGCTCAGTATGACGATGTTGCC ACTTGCTACGGTACCAGGACTCAG 66 Yes 3.8359**

**GTCAAGATGGACAACAGCCGAGAC TGGCCTTCATCTCCTCACACTTGC 139 Yes+ 5.3382**

**AAGGCTCAGTATGACGATGTTGCC TGGCCTTCATCTCCTCACACTTGC 85 Yes+ 5.7187**

**AGGCTCAGTATGACGATGTTGCC GCCTTCATCTCCTCACACTTGC 82 Yes+ 3.9258**

**AGGCTCAGTATGACGATGTTGCC TGGCCTTCATCTCCTCACACTTG 84 Yes+ 3.9804**

**AGGCTCAGTATGACGATGTTGCC GTGGCCTTCATCTCCTCACACTTG 85 Yes+ 4.0115**

**TCAAGATGGACAACAGCCGAGAC GCCTTCATCTCCTCACACTTGC 136 Yes+ 4.0527**

**TCAAGATGGACAACAGCCGAGAC TGGCCTTCATCTCCTCACACTTG 138 Yes+ 4.1073**

**TCAAGATGGACAACAGCCGAGAC GTGGCCTTCATCTCCTCACACTTG 139 Yes+ 4.1385**

**GGCTCAGTATGACGATGTTGCC GCCTTCATCTCCTCACACTTGC 81 Yes+ 4.1563**

**NM_003116.1**

**TGCTGAGCCTCTTTCTGTCAGC TCCAAAGGAGAGACCAGGTACAG 61 Yes+ 4.388**

**TGCTGAGCCTCTTTCTGTCAGC CTCCAAAGGAGAGACCAGGTACAG 62 Yes+ 4.5708**

**TCGTGTTCCAGAGGCTGAATGAG GCTCCCACAGAGCTCAAAGCATAG 67 Yes 4.277**

**CTCGTGTTCCAGAGGCTGAATGAG GCTCCCACAGAGCTCAAAGCATAG 68 Yes 4.5487**

**TCGTGTTCCAGAGGCTGAATGAGG GCTCCCACAGAGCTCAAAGCATAG 67 Yes 4.9328**

**TCGTGTTCCAGAGGCTGAATGAG TCCCACAGAGCTCAAAGCATAGTC 65 Yes 5.3695**

**TCAGCATGTACAGGGAGGTCTG CTGACAGAAAGAGGCTCAGCAG 75 Yes 4.8765**

**AGAGCTTTCTGAGCCTGCTCTTC ACGTCTCCTGCCAGGGATAACAAC 61 Yes 4.9796**

**TGTACAGGGAGGTCTGTTCCATCC AGCCAGAATGCTGACAGAAAGAGG 79 Yes 4.9158**

**TGTACAGGGAGGTCTGTTCCATCC GCCAGAATGCTGACAGAAAGAGG 78 Yes 5.1068**

**NM_003488.3**

**TACAGCACTGCTTGCTCAGGTGAC CCAACCACACTCCACAGCTGAATC 75 Yes+ 5.7295**

**GTCCAGATCTGCCACATAGAAGGC TTCCCAATCAAGTTCAGCGCTTTG 65 Yes+ 5.64**

**CCAGATCTGCCACATAGAAGGC CCCAATCAAGTTCAGCGCTTTG 61 Yes+ 5.5605**

**GTCCAGATCTGCCACATAGAAGGC TCCCAATCAAGTTCAGCGCTTTG 64 Yes+ 5.0557**

**TCCAGATCTGCCACATAGAAGGC CCCAATCAAGTTCAGCGCTTTG 62 Yes+ 5.2888**

**TCCAGATCTGCCACATAGAAGGC TTCCCAATCAAGTTCAGCGCTTTG 64 Yes+ 5.6309**

**CCAGATCTGCCACATAGAAGGC TCCCAATCAAGTTCAGCGCTTTG 62 Yes+ 5.3184**

**TCCAGATCTGCCACATAGAAGGC TCCCAATCAAGTTCAGCGCTTTG 63 Yes+ 5.0467**

**GTCCAGATCTGCCACATAGAAGGC CCCAATCAAGTTCAGCGCTTTGTC 63 Yes+ 5.0883**

**TCCAGATCTGCCACATAGAAGGC CCCAATCAAGTTCAGCGCTTTGTC 62 Yes+ 5.0793**

**NM_004173.2**

**CCTGAGCATCGTCCTCAACATCTG ACACTGCAAGTCCCATCAGCAG 95 Yes 2.9226**

**CTGAGCATCGTCCTCAACATCTG ACACTGCAAGTCCCATCAGCAG 94 Yes 3.9142**

**CCTGAGCATCGTCCTCAACATCTG TACACTGCAAGTCCCATCAGCAG 96 Yes 4.2692**

**TCAACATCTGCCTCATGCTGAAAC ACACTGCAAGTCCCATCAGCAG 81 Yes 4.3199**

**CCTGAGCATCGTCCTCAACATC TACACTGCAAGTCCCATCAGCAG 96 Yes 4.488**

**ATCTGGCTGCTGATGGGACTTG CACGTAGTGTGTGGAGTTCAGC 102 Yes 3.2041**

**ATCTGGCTGCTGATGGGACTTG GTTCTCCTTGCTATGCCGGATG 63 Yes 3.2439**

**ATCTGGCTGCTGATGGGACTTG ACCACGTAGTGTGTGGAGTTCAG 104 Yes 3.2702**

**ATCTGGCTGCTGATGGGACTTG CCACGTAGTGTGTGGAGTTCAG 103 Yes 3.7662**

**ATCTGGCTGCTGATGGGACTTG TACCACGTAGTGTGTGGAGTTCAG 105 Yes 4.5718**

**NM_004211.3**

**TGAGAATGTGGCAGACCAAGGG AGAGAGAGGCAGCCTGGTTAAG 76 Yes+ 3.2605**

**TGAGAATGTGGCAGACCAAGGG CCTGGTTAAGGCTTCCGGGTAAAC 64 Yes+ 3.3065**

**TTGAGAATGTGGCAGACCAAGGG AGAGAGAGGCAGCCTGGTTAAG 77 Yes+ 3.8346**

**TTGAGAATGTGGCAGACCAAGGG CCTGGTTAAGGCTTCCGGGTAAAC 65 Yes+ 3.8805**

**TGAGAATGTGGCAGACCAAGGG CTGGTTAAGGCTTCCGGGTAAAC 63 Yes+ 4.3213**

**ATTGAGAATGTGGCAGACCAAGGG AGAGAGAGGCAGCCTGGTTAAG 78 Yes+ 4.6146**

**TGAGAATGTGGCAGACCAAGGG GGTTAAGGCTTCCGGGTAAACCAC 61 Yes+ 3.1316**

**TTGAGAATGTGGCAGACCAAGGG GGTTAAGGCTTCCGGGTAAACCAC 62 Yes+ 3.7057**

**ATTGAGAATGTGGCAGACCAAGGG GGTTAAGGCTTCCGGGTAAACCAC 63 Yes+ 4.4856**

**TGAGAATGTGGCAGACCAAGGG GTTAAGGCTTCCGGGTAAACCAC 60 Yes+ 4.0772**

**NM_004238.1**

**TCAGTACTTCTACCCGGAGGAAC GTCTTTGCATCCCAAGTGTCTGC 72 Yes+ 5.9514**

**TCAGTACTTCTACCCGGAGGAAC TCTTTGCATCCCAAGTGTCTGC 71 Yes+ 6.0093**

**TTCAGTACTTCTACCCGGAGGAAC GTCTTTGCATCCCAAGTGTCTGC 73 Yes+ 6.4743**

**TTCAGTACTTCTACCCGGAGGAAC TCTTTGCATCCCAAGTGTCTGC 72 Yes+ 6.5323**

**TCAGTACTTCTACCCGGAGGAAC TGCATCCCAAGTGTCTGCTTTAC 67 Yes+ 7.0367**

**TCAGTACTTCTACCCGGAGGAAC TCCCAAGTGTCTGCTTTACTGC 63 Yes+ 6.5944**

**AGTACTTCTACCCGGAGGAACTG GTCTTTGCATCCCAAGTGTCTGC 70 Yes+ 5.949**

**AGTACTTCTACCCGGAGGAACTG TCTTTGCATCCCAAGTGTCTGC 69 Yes+ 6.0069**

**AGTACTTCTACCCGGAGGAACTG TGCATCCCAAGTGTCTGCTTTAC 65 Yes+ 7.0343**

**AGTACTTCTACCCGGAGGAACTG TCCCAAGTGTCTGCTTTACTGC 61 Yes+ 6.592**

**NM_004514.3**

**GGAGACCACAGGGAAGTCAAAGTG TCTGTGCCGTCTGAATGATCCG 97 Yes+ 3.5421**

**GAGACCACAGGGAAGTCAAAGTG TCTGTGCCGTCTGAATGATCCG 96 Yes+ 4.5718**

**GGAGACCACAGGGAAGTCAAAGTG GTCTGTGCCGTCTGAATGATCC 98 Yes+ 4.8981**

**GGAGACCACAGGGAAGTCAAAGTG TGGTCTGTGCCGTCTGAATGATCC 100 Yes+ 5.664**

**GAGACCACAGGGAAGTCAAAGTG GTCTGTGCCGTCTGAATGATCC 97 Yes+ 5.9279**

**GGAGACCACAGGGAAGTCAAAGTG AGAGGTGCCTGTTGTACTATGGTC 140 Yes+ 6.2982**

**GAGACCACAGGGAAGTCAAAGTG AGAGGTGCCTGTTGTACTATGGTC 139 Yes+ 7.328**

**GAGACCACAGGGAAGTCAAAGTG GAGGTGCCTGTTGTACTATGGTC 138 Yes+ 7.5537**

**GCCCGAAGGATGATTCAAAGCC TTGTCGGGAGCCATCGTAATCG 76 Yes 2.4162**

**AGCCCGAAGGATGATTCAAAGCC TTGTCGGGAGCCATCGTAATCG 77 Yes 2.1882**

**NM_004808.2**

**TTGACACGTTTGTAGTGGAGAGC GCTTTGAGGCTCTTGTGAGCAG 109 Yes+ 5.2362**

**TTGACACGTTTGTAGTGGAGAGC AGAATGAGTAGGCGGCTTTGAGG 123 Yes+ 5.6826**

**ATTGACACGTTTGTAGTGGAGAGC GCTTTGAGGCTCTTGTGAGCAG 110 Yes+ 5.9492**

**ATTGACACGTTTGTAGTGGAGAGC AGAATGAGTAGGCGGCTTTGAGG 124 Yes+ 6.3955**

**TTGACACGTTTGTAGTGGAGAGC GCGTCTCTGTGTGGATGTTGTAG 144 Yes+ 6.7899**

**TTGACACGTTTGTAGTGGAGAGC TAGAATGAGTAGGCGGCTTTGAGG 124 Yes+ 6.9951**

**ATTGACACGTTTGTAGTGGAGAGC GCGTCTCTGTGTGGATGTTGTAG 145 Yes+ 7.5029**

**TTGACACGTTTGTAGTGGAGAGC TGTAGAATGAGTAGGCGGCTTTG 126 Yes+ 7.5664**

**TTGACACGTTTGTAGTGGAGAGC GAATGAGTAGGCGGCTTTGAGG 122 Yes+ 5.9316**

**ATTGACACGTTTGTAGTGGAGAGC GAATGAGTAGGCGGCTTTGAGG 123 Yes+ 6.6445**

**NM_004844.3**

**ACGGGAGACTGAACTTGAGGATG AGTTTCACCGTTGCTTCAACCAG 69 Yes+ 5.3103**

**ACGGGAGACTGAACTTGAGGATG CAGTTTCACCGTTGCTTCAACCAG 70 Yes+ 5.3384**

**GACGGGAGACTGAACTTGAGGATG AGTTTCACCGTTGCTTCAACCAG 70 Yes+ 5.5543**

**ACGGGAGACTGAACTTGAGGATG GTTTCACCGTTGCTTCAACCAG 68 Yes+ 5.558**

**GACGGGAGACTGAACTTGAGGATG CAGTTTCACCGTTGCTTCAACCAG 71 Yes+ 5.5824**

**GACGGGAGACTGAACTTGAGGATG GTTTCACCGTTGCTTCAACCAG 69 Yes+ 5.802**

**ACGGGAGACTGAACTTGAGGATGC CAGTTTCACCGTTGCTTCAACCAG 70 Yes 5.4556**

**GGGAGACTGAACTTGAGGATGC AGTTTCACCGTTGCTTCAACCAG 67 Yes 5.5735**

**GGGAGACTGAACTTGAGGATGC GTTTCACCGTTGCTTCAACCAG 66 Yes 5.8211**

**ACGGGAGACTGAACTTGAGGATG CGTTGCTTCAACCAGAACAGAGC 61 Yes+ 4.6828**

**NM_005120.2**

**ACTGCTGGACAATGAGGATGGG TCCACTGGTCCAAGTTCTGGAG 77 Yes+ 3.3144**

**GCAAAGGGACAGCAGAAACTGAC TGTCAGGTCTCCAGGATTCAGAGG 65 Yes+ 4.689**

**TCAAGGCTGTGTTTGTACTTGGG TTCCTCCTGTCACAGTGAAGCC 66 Yes+ 4.8391**

**TGCTATGGTGGTAGCCAAGCTC TTCTGATTCTCCACAACGCTCAG 73 Yes+ 5.0343**

**CGACTGCTGGACAATGAGGATG TCCACTGGTCCAAGTTCTGGAG 79 Yes+ 4.3567**

**AGCCAGTCTGGATGTCTATGCC ACGTTCTCCTACCCATTCCTGTTG 70 Yes+ 5.0265**

**TGGCCATGTTCATGTTTCAGGATG AACACTCAAGCACCCAGGTCAG 69 Yes+ 4.673**

**TCGACTGCTGGACAATGAGGATG TCCACTGGTCCAAGTTCTGGAG 80 Yes+ 4.1185**

**GACTGCTGGACAATGAGGATGG TCCACTGGTCCAAGTTCTGGAG 78 Yes+ 4.6608**

**CAGCAACAGACAGCAGCTTTGG TACTGGGCTGTGGCTGGGTATTAG 70 Yes+ 3.0467**

**NM_005552.4**

**ATGAGCGTAGAGTGGAACGGGATG ACGCTGAGCAAGTCAGTTAACCAG 66 Yes+ 4.9765**

**ATGAGCGTAGAGTGGAACGGGATG CATGCAGGAGACAGTGTGAGAGTC 110 Yes+ 5.3897**

**ATGAGCGTAGAGTGGAACGGGATG GTCATGCAGGAGACAGTGTGAGAG 112 Yes+ 5.3897**

**NM_005759.4**

**ACGCCCTCCTTCCATTACTTCAC AGGAGGAGGAGCAAGAGATACTGG 97 Yes+ 4.9873**

**GACGCCCTCCTTCCATTACTTCAC AGGAGGAGGAGCAAGAGATACTGG 98 Yes+ 5.2609**

**CGCCCTCCTTCCATTACTTCAC AGGAGGAGGAGCAAGAGATACTGG 96 Yes+ 5.4972**

**AGAACAGTGGAAGTGGTAGTGTGG AGGATGACCTGGAAAGACACTGG 80 Yes 6.1167**

**ACAGTCAGCAGATAAGCAGAGAGC AGGCAACACTTGCTAAGGATTGGG 74 Yes 5.3947**

**ACAGTCAGCAGATAAGCAGAGAGC GGCAACACTTGCTAAGGATTGGG 73 Yes 5.5783**

**ACAGTCAGCAGATAAGCAGAGAGC ACACTTGCTAAGGATTGGGTGGTG 69 Yes 5.697**

**ACAGTCAGCAGATAAGCAGAGAGC TGCTAAGGATTGGGTGGTGTAGG 64 Yes 5.841**

**ACAGTCAGCAGATAAGCAGAGAGC CACTTGCTAAGGATTGGGTGGTG 68 Yes 6.1333**

**ACAGTCAGCAGATAAGCAGAGAGC AAGGATTGGGTGGTGTAGGCTTTG 60 Yes 5.679**

**NM_006300.3**

**AAGGAAATTCAGGCGGCAAGAC AGATTTGCTGGCAAGGGCAGTC 69 Yes 1.9727**

**AGAAGGAAATTCAGGCGGCAAGAC AGATTTGCTGGCAAGGGCAGTC 71 Yes 2.0771**

**GGCACAATTCCACCTTCCTTTGTG CTTGAAGGTCACTGCCTCCTTG 102 Yes+ 5.328**

**AGGCACAATTCCACCTTCCTTTG CTTGAAGGTCACTGCCTCCTTG 103 Yes+ 5.5769**

**CACAATTCCACCTTCCTTTGTGC CTTGAAGGTCACTGCCTCCTTG 100 Yes+ 6.3559**

**GCACAATTCCACCTTCCTTTGTG CTTGAAGGTCACTGCCTCCTTG 101 Yes+ 6.3559**

**ACAATTCCACCTTCCTTTGTGCTC CTTGAAGGTCACTGCCTCCTTG 99 Yes+ 6.3795**

**ACCTTCCTTTGTGCTCCATTACTC CTTGAAGGTCACTGCCTCCTTG 91 Yes+ 7.3398**

**GGCACAATTCCACCTTCCTTTGTG TCCTTGAAGGTCACTGCCTCCTTG 104 Yes+ 5.5676**

**CCACCTTCCTTTGTGCTCCATTAC CTTGAAGGTCACTGCCTCCTTG 93 Yes+ 6.2726**

**NM_006956.2**

**(no primer pairs matching criteria)**

**NM_016474.4**

**TTAGAGATGCTGCGTGAACAGAC TTGCCTCTAAGATAGCCTTTCGC 85 Yes+ 7.629**

**TTAGAGATGCTGCGTGAACAGAC CTTGCCTCTAAGATAGCCTTTCGC 86 Yes+ 7.8119**

**CTTAGAGATGCTGCGTGAACAGAC TTGCCTCTAAGATAGCCTTTCGC 86 Yes+ 7.83**

**AGAGATGCTGCGTGAACAGAC TTGCCTCTAAGATAGCCTTTCGC 83 Yes+ 7.8801**

**CTTAGAGATGCTGCGTGAACAGAC CTTGCCTCTAAGATAGCCTTTCGC 87 Yes+ 8.0129**

**AGAGATGCTGCGTGAACAGAC CTTGCCTCTAAGATAGCCTTTCGC 84 Yes+ 8.063**

**ATGCTGCGTGAACAGACAACAG TTGCCTCTAAGATAGCCTTTCGC 79 Yes 5.6056**

**ATGCTGCGTGAACAGACAACAG CTTGCCTCTAAGATAGCCTTTCGC 80 Yes 5.7885**

**GATGCTGCGTGAACAGACAACAG TTGCCTCTAAGATAGCCTTTCGC 80 Yes 5.8376**

**TGGGTGGATTACGTGGACTCTTTG CCAGCAGATCTGGCAAATCCTTTC 70 Yes 6.4388**

**NM_016564.3**

**AGCCAGTGCATGCAGAGTTCAC AGGTGTGTAATGAATGTGCGTGTG 61 No 3.5556**

**AAGCCAGTGCATGCAGAGTTCAC AGGTGTGTAATGAATGTGCGTGTG 62 No 4.9261**

**ACTTCCTGTACAGACCCGAGGAAG TGCGTGTGTACGAATAGGTAAGGG 67 No 5.4128**

**TTCCTGTACAGACCCGAGGAAG TGCGTGTGTACGAATAGGTAAGGG 65 No 5.6779**

**TTCCTGTACAGACCCGAGGAAG GCGTGTGTACGAATAGGTAAGGG 64 No 6.1043**

**TTCCTGTACAGACCCGAGGAAG GGTGTGTAATGAATGTGCGTGTG 80 No 6.236**

**ACTTCCTGTACAGACCCGAGGAAG GTGCGTGTGTACGAATAGGTAAGG 68 No 6.391**

**TTCCTGTACAGACCCGAGGAAG GTGCGTGTGTACGAATAGGTAAGG 66 No 6.6562**

**ACTTCCTGTACAGACCCGAGGAAG TGAATGTGCGTGTGTACGAATAGG 73 No 6.4396**

**TTCCTGTACAGACCCGAGGAAG TGAATGTGCGTGTGTACGAATAGG 71 No 6.7048**

**NM_017856.2**

**AGGGCATCTAAATCTGCAGCAAC GTCCAAATGGGTGCTGAAACTCTC 66 Yes+ 6.6794**

**AGGGCATCTAAATCTGCAGCAAC TCCAAATGGGTGCTGAAACTCTC 65 Yes+ 6.7213**

**AAGGGCATCTAAATCTGCAGCAAC GTCCAAATGGGTGCTGAAACTCTC 67 Yes+ 7.2315**

**AAGGGCATCTAAATCTGCAGCAAC TCCAAATGGGTGCTGAAACTCTC 66 Yes+ 7.2734**

**AGACCAAAGGGCATCTAAATCTGC GTCCAAATGGGTGCTGAAACTCTC 73 Yes+ 8.0001**

**AGGGCATCTAAATCTGCAGCAAC AAGTCCAAATGGGTGCTGAAACTC 68 Yes+ 7.0128**

**AAGGGCATCTAAATCTGCAGCAAC AAGTCCAAATGGGTGCTGAAACTC 69 Yes+ 7.5649**

**AGGGCATCTAAATCTGCAGCAAC AGTCCAAATGGGTGCTGAAACTC 67 Yes+ 6.4701**

**AAGGGCATCTAAATCTGCAGCAAC AGTCCAAATGGGTGCTGAAACTC 68 Yes+ 7.0222**

**AGACCAAAGGGCATCTAAATCTGC AGTCCAAATGGGTGCTGAAACTC 74 Yes+ 7.7908**

**NM_018225.2**

**TGCTGTCCTCTGCATGTGTTTC CCACTCTGAATCTTCCACACCTTG 93 Yes+ 6.5579**

**ATGATGCTGTCCTCTGCATGTG CCACTCTGAATCTTCCACACCTTG 97 Yes+ 6.9389**

**ATGATGGATGATGCTGTCCTCTGC CCACTCTGAATCTTCCACACCTTG 104 Yes+ 7.0825**

**GATGGATGATGCTGTCCTCTGC CCACTCTGAATCTTCCACACCTTG 102 Yes+ 6.8172**

**GCTGTCCTCTGCATGTGTTTCAG CCACTCTGAATCTTCCACACCTTG 92 Yes+ 6.7439**

**TGATGGATGATGCTGTCCTCTGC CCACTCTGAATCTTCCACACCTTG 103 Yes+ 6.3139**

**CTGTCCTCTGCATGTGTTTCAGC CCACTCTGAATCTTCCACACCTTG 91 Yes+ 6.7439**

**GTCCTCTGCATGTGTTTCAGCAG CCACTCTGAATCTTCCACACCTTG 89 Yes+ 6.7439**

**TCCTCTGCATGTGTTTCAGCAG CCACTCTGAATCTTCCACACCTTG 88 Yes+ 6.8134**

**TGTCCTCTGCATGTGTTTCAGC CCACTCTGAATCTTCCACACCTTG 90 Yes+ 6.5579**

**NM_018246.2**

**TTCATGTGGACAAACTCTCTTCGG ACGTTGTTCATCTTGCAGCCTTG 145 Yes+ 6.856**

**TTCATGTGGACAAACTCTCTTCGG CGTTGTTCATCTTGCAGCCTTG 144 Yes+ 7.3366**

**TTCATGTGGACAAACTCTCTTCGG AACGTTGTTCATCTTGCAGCCTTG 146 Yes+ 7.452**

**TCAAGGCTGCAAGATGAACAACG ACATCCTTCTGCCTGTGAAAGCC 111 Yes 3.8783**

**TCAAGGCTGCAAGATGAACAACG CATCCTTCTGCCTGTGAAAGCC 110 Yes 4.3646**

**TCAAGGCTGCAAGATGAACAACG TCTGCCTGTGAAAGCCTATCTGC 104 Yes 4.3304**

**CAAGGCTGCAAGATGAACAACG TCTGCCTGTGAAAGCCTATCTGC 103 Yes 4.5659**

**TCACCAGCAGCAGCGTTAATTC TGAGCCGAAGAGAGTTTGTCCAC 133 Yes 3.8682**

**TCACCAGCAGCAGCGTTAATTC GAGCCGAAGAGAGTTTGTCCAC 132 Yes 4.3535**

**TTCACCAGCAGCAGCGTTAATTC TGAGCCGAAGAGAGTTTGTCCAC 134 Yes 4.421**

**NM_020637.1**

**CGTCGTGGTCATCAAAGCAGTG TTGTGGCCGTTCTCTTCGATGC 129 No 1.3806**

**TCGTGGTCATCAAAGCAGTGTCC TTGTGGCCGTTCTCTTCGATGC 127 No 1.455**

**CGTGGTCATCAAAGCAGTGTCC TTGTGGCCGTTCTCTTCGATGC 126 No 1.6649**

**TCAAAGCAGTGTCCTCAGGCTTC TTGTGGCCGTTCTCTTCGATGC 118 No 1.7325**

**AGCAGTGTCCTCAGGCTTCTAC TTGTGGCCGTTCTCTTCGATGC 114 No 1.9295**

**AGTGTCCTCAGGCTTCTACGTG TTGTGGCCGTTCTCTTCGATGC 111 No 1.9452**

**CAAAGCAGTGTCCTCAGGCTTC TTGTGGCCGTTCTCTTCGATGC 117 No 1.9638**

**CGTCGTGGTCATCAAAGCAGTG AGGTGTTGTGGCCGTTCTCTTC 134 No 1.9892**

**TCGTGGTCATCAAAGCAGTGTCC AGGTGTTGTGGCCGTTCTCTTC 132 No 2.0637**

**CGTGGTCATCAAAGCAGTGTCC AGGTGTTGTGGCCGTTCTCTTC 131 No 2.2736**

**NM_021010.1**

**AGGCTACAACCCAGAAGCAGTC TCCTGCAAAGGAGATAGCAAGGTC 62 No 4.5741**

**AGGCTGAGTCACTCCAGGAAAGAG TAGCAAGGTCCTGGTTGTCTTCC 78 No 4.5213**

**AGGCTGAGTCACTCCAGGAAAG TAGCAAGGTCCTGGTTGTCTTCC 78 No 4.4545**

**AGGCTGAGTCACTCCAGGAAAG AGCAAGGTCCTGGTTGTCTTCC 77 No 3.1293**

**AGGCTGAGTCACTCCAGGAAAGAG AGCAAGGTCCTGGTTGTCTTCC 77 No 3.1961**

**CTGAGTCACTCCAGGAAAGAGCTG AGCAAGGTCCTGGTTGTCTTCC 74 No 4.1633**

**TGAGTCACTCCAGGAAAGAGCTG AGCAAGGTCCTGGTTGTCTTCC 73 No 3.9296**

**CACTCCAGGAAAGAGCTGATGAGG AGCAAGGTCCTGGTTGTCTTCC 68 No 4.0875**

**ACTCCAGGAAAGAGCTGATGAGG AGCAAGGTCCTGGTTGTCTTCC 67 No 4.1011**

**CCAGGAAAGAGCTGATGAGGCTAC AGCAAGGTCCTGGTTGTCTTCC 64 No 4.0318**

**NM_023110.2**

**(no primer pairs matching criteria)**

**NM_024101.5**

**ACCTCTGATGAGGAAAGCATCCG TGTGTTCTCCAGGTAGGTCAGCAG 147 No 4.5727**

**CACCTCTGATGAGGAAAGCATCCG TGTGTTCTCCAGGTAGGTCAGCAG 148 No 4.602**

**ACACCTCTGATGAGGAAAGCATCC TGTGTTCTCCAGGTAGGTCAGCAG 149 No 5.4382**

**ACCTCTGATGAGGAAAGCATCCG TGTTCTCCAGGTAGGTCAGCAG 145 No 4.5519**

**CACCTCTGATGAGGAAAGCATCC TGTTCTCCAGGTAGGTCAGCAG 146 No 5.8757**

**CACCTCTGATGAGGAAAGCATCCG TGTTCTCCAGGTAGGTCAGCAG 146 No 4.5812**

**CCTCTGATGAGGAAAGCATCCG TGTTCTCCAGGTAGGTCAGCAG 144 No 5.0669**

**ACACCTCTGATGAGGAAAGCATCC TGTTCTCCAGGTAGGTCAGCAG 147 No 5.4174**

**GACACCTCTGATGAGGAAAGCATC TGTTCTCCAGGTAGGTCAGCAG 148 No 6.654**

**CCTCTGATGAGGAAAGCATCCG AACTGTGTTCTCCAGGTAGGTCAG 149 No 6.6958**

**NM_024112.3**

**GCAGAATACGCTGCCATCAACTC CGTGGAGGTGGTCATTCTTCTC 85 Yes 5.2504**

**AGCAGAATACGCTGCCATCAAC AGGTGGTCATTCTTCTCCTCCAG 81 Yes 5.3135**

**GCAGAATACGCTGCCATCAACTC AGGTGGTCATTCTTCTCCTCCAG 80 Yes 5.5195**

**AGCAGAATACGCTGCCATCAAC GTCATTCTTCTCCTCCAGGTGGTC 76 Yes 5.2815**

**AAGCAGAATACGCTGCCATCAAC GTCATTCTTCTCCTCCAGGTGGTC 77 Yes 5.825**

**GCAGAATACGCTGCCATCAACTCC GTCATTCTTCTCCTCCAGGTGGTC 75 Yes 5.8665**

**GAAGCAGAATACGCTGCCATCAAC GTCATTCTTCTCCTCCAGGTGGTC 78 Yes 6.0866**

**AGCAGAATACGCTGCCATCAAC TCATTCTTCTCCTCCAGGTGGTC 75 Yes 5.3171**

**AAGCAGAATACGCTGCCATCAAC TCATTCTTCTCCTCCAGGTGGTC 76 Yes 5.8605**

**GAAGCAGAATACGCTGCCATCAAC TCATTCTTCTCCTCCAGGTGGTC 77 Yes 6.1221**

**NM_032307.3**

**CCTCGACGAAGGGATACCAATAAC AAGGCATGGAAACGTCTGTGTC 104 Yes 7.1074**

**CCTCGACGAAGGGATACCAATAAC AAAGGCATGGAAACGTCTGTGTC 105 Yes 7.6409**

**CCTCGACGAAGGGATACCAATAAC CGGTTTCATTGAGAATCCGATGCC 140 Yes 7.945**

**CCTCGACGAAGGGATACCAATAAC ACTAAAGGCATGGAAACGTCTGTG 108 Yes 8.9184**

**CCTCGACGAAGGGATACCAATAAC TAAAGGCATGGAAACGTCTGTGTC 106 Yes 8.9221**

**CCTCGACGAAGGGATACCAATAAC GCCTCTCTTCTACTAAAGGCATGG 119 Yes 9.7457**

**CCTCGACGAAGGGATACCAATAAC TTCATTGAGAATCCGATGCCTCTC 136 Yes 9.7462**

**CCTCGACGAAGGGATACCAATAAC AGGCATGGAAACGTCTGTGTC 103 Yes 8.6171**

**CCTCGACGAAGGGATACCAATAAC AACCTGATCCAGGGTCACTGTC 64 Yes 6.8019**

**TGAGGAGGGTGGATTGCTTGAG AGAATGCAGTGGCGTGACTGTG 70 No 1.6522**

**NM_033396.2**

**ACCTCCCAAGCCAGAGAAATCC AGGTGGGAAGAACCTCAGTGAC 113 Yes 3.6297**

**ATCCTCAGGGTCAGAAGGATCG AGGTGGGAAGAACCTCAGTGAC 95 Yes 4.638**

**TACCTCCCAAGCCAGAGAAATCC AGGTGGGAAGAACCTCAGTGAC 114 Yes 4.9395**

**AATCCTCAGGGTCAGAAGGATCG AGGTGGGAAGAACCTCAGTGAC 96 Yes 5.1448**

**TTACCTCCCAAGCCAGAGAAATCC AGGTGGGAAGAACCTCAGTGAC 115 Yes 5.4924**

**AAATCCTCAGGGTCAGAAGGATCG AGGTGGGAAGAACCTCAGTGAC 97 Yes 5.6926**

**AGAGAAATCCTCAGGGTCAGAAGG AGGTGGGAAGAACCTCAGTGAC 101 Yes 6.2841**

**GTTACCTCCCAAGCCAGAGAAATC AGGTGGGAAGAACCTCAGTGAC 116 Yes 6.4949**

**ATCCTCAGGGTCAGAAGGATCGTC ACCTCAGTGACTTCTCAGACCTTC 84 Yes 6.4406**

**ATCCTCAGGGTCAGAAGGATCGTC AGAACCTCAGTGACTTCTCAGACC 87 Yes 6.4406**

**NM_057088.2**

**TCAGGCCAAAGTGGATGCCTTG TGAGATAGCTCAGCGTCGTAGAGG 72 Yes 2.5647**

**AGCTTCAGGCCAAAGTGGATGC TGAGATAGCTCAGCGTCGTAGAGG 76 Yes 2.3521**

**TGCCTCCTTCATTGACAAGGTG GTGCCTGAGATGGAACTTGTGC 105 Yes+ 4.4511**

**TGCCTCCTTCATTGACAAGGTG GCTCAAGGTTGTTTGTGCCTGAG 119 Yes+ 4.8326**

**TGCCTCCTTCATTGACAAGGTG AGAGGCTCAAGGTTGTTTGTGC 123 Yes+ 4.8777**

**TGCCTCCTTCATTGACAAGGTG TCCACTTGGTCTCCAGGACTTTG 65 Yes+ 5.3788**

**TGCCTCCTTCATTGACAAGGTG AAGAGGCTCAAGGTTGTTTGTGC 124 Yes+ 5.4168**

**TGCCTCCTTCATTGACAAGGTG CCACTTGGTCTCCAGGACTTTG 64 Yes+ 5.6547**

**TGCCTCCTTCATTGACAAGGTG TGTGCCTGAGATGGAACTTGTG 106 Yes+ 5.6553**

**TGCCTCCTTCATTGACAAGGTG GGTTGTTTGTGCCTGAGATGGAAC 113 Yes+ 5.8925**

**NM_130854.2**

**(no primer pairs matching criteria)**

**NM_133334.2**

**ATCTGACTGCGGCTCATACAGG GTGAACACATTCCAGCCAACTGC 71 No 3.1764**

**ATCTGACTGCGGCTCATACAGG TGAACACATTCCAGCCAACTGC 70 No 3.2024**

**TGGGACCGTACAGGTGAATGTG TGACGTGACTCACTCGGAGAAG 60 No 3.342**

**TTGGGACCGTACAGGTGAATGTG TGACGTGACTCACTCGGAGAAG 61 No 3.9076**

**TGTTGGGACCGTACAGGTGAATG TGACGTGACTCACTCGGAGAAG 63 No 3.9076**

**ATCTGACTGCGGCTCATACAGG AGTGAACACATTCCAGCCAACTGC 72 No 3.9085**

**GTTGGGACCGTACAGGTGAATGTG TGACGTGACTCACTCGGAGAAG 62 No 3.9577**

**AAGAGTAGGTGAGCGCTTTGCAG ACCTGTATGAGCCGCAGTCAGATG 60 No 4.2953**

**ATCTGACTGCGGCTCATACAGGTC GTGAACACATTCCAGCCAACTGC 71 No 4.3875**

**TGAAGAGTAGGTGAGCGCTTTGC ACCTGTATGAGCCGCAGTCAGATG 62 No 4.3029**

**NM_138716.2**

**(no primer pairs matching criteria)**

**NM_172367.2**

**TGTCTCGAAGCAGCATGCAACAG GGTCACGGCCACCATGATAATGAC 122 Yes 3.5543**

**TTACCCTCATCATCATGGGCATCG AACTGTGAAGTTGACGGTCACG 62 Yes 5.363**

**TTACCCTCATCATCATGGGCATCG GAACTGTGAAGTTGACGGTCACG 63 Yes 5.5615**

**TTACCCTCATCATCATGGGCATCG ACTGTGAAGTTGACGGTCACG 61 Yes 6.8692**

**TCTCGAAGCAGCATGCAACAGG GAACTGTGAAGTTGACGGTCACG 136 Yes 2.5796**

**GTCTCGAAGCAGCATGCAACAG AACTGTGAAGTTGACGGTCACG 136 Yes 3.3843**

**TACCCTCATCATCATGGGCATCG AACTGTGAAGTTGACGGTCACG 61 Yes 4.7846**

**TACCCTCATCATCATGGGCATCG GAACTGTGAAGTTGACGGTCACG 62 Yes 4.983**

**TACCCTCATCATCATGGGCATCG ACTGTGAAGTTGACGGTCACG 60 Yes 6.2907**

**ACCCTCATCATCATGGGCATCG AACTGTGAAGTTGACGGTCACG 60 Yes 3.4583**

**NM_182690.2**

**TCTGTCTGCTGCAAGGAGAGGAAC TCCATCTTGTCGGTCTGAATTGGC 128 Yes+ 5.2112**

**TCTGTCTGCTGCAAGGAGAGGAAC TCAATGCTCCATCTTGTCGGTCTG 135 Yes+ 5.4596**

**TGTCTGCTGCAAGGAGAGGAAC AATGCTCCATCTTGTCGGTCTG 131 Yes+ 4.0989**

**TGTCTGCTGCAAGGAGAGGAAC TCCATCTTGTCGGTCTGAATTGGC 126 Yes+ 3.5807**

**TGTCTGCTGCAAGGAGAGGAAC CCATCTTGTCGGTCTGAATTGGC 125 Yes+ 3.7511**

**TGTCTGCTGCAAGGAGAGGAAC TCAATGCTCCATCTTGTCGGTCTG 133 Yes+ 3.8291**

**TGTCTGCTGCAAGGAGAGGAAC CCCATCAATGCTCCATCTTGTCG 137 Yes+ 3.9303**

**TGTCTGCTGCAAGGAGAGGAAC CAATGCTCCATCTTGTCGGTCTG 132 Yes+ 4.0033**

**TGTCTGCTGCAAGGAGAGGAAC CTCCATCTTGTCGGTCTGAATTGG 127 Yes+ 5.3268**

**TGTCTGCTGCAAGGAGAGGAAC GCTCCATCTTGTCGGTCTGAATTG 128 Yes+ 4.8087**

**NM_198535.1**

**ACTAGGGTATCCGCTACACACACC TCTGCAGGTCCTCCTCTTGTTC 65 Yes 4.0846**

**ACTAGGGTATCCGCTACACACACC TCTCTTCACTGTCTGCAGGTCCTC 76 Yes 4.122**

**TAGGGTATCCGCTACACACACC TCTGCAGGTCCTCCTCTTGTTC 63 Yes 4.3318**

**ACTAGGGTATCCGCTACACACACC TGTCTGCAGGTCCTCCTCTTGTTC 67 Yes 4.6085**

**CACTAGGGTATCCGCTACACACAC TCTGCAGGTCCTCCTCTTGTTC 66 Yes 5.0966**

**ACTAGGGTATCCGCTACACACACC TCTTCACTGTCTGCAGGTCCTC 74 Yes 4.0955**

**TAGGGTATCCGCTACACACACC TCTTCACTGTCTGCAGGTCCTC 72 Yes 4.3427**

**AGAACCTGGCCTCACTAGGGTATC TCTGCAGGTCCTCCTCTTGTTC 78 Yes 4.5698**

**AGAACCTGGCCTCACTAGGGTATC TGTCTGCAGGTCCTCCTCTTGTTC 80 Yes 5.0937**

**AACCTGGCCTCACTAGGGTATC TCTGCAGGTCCTCCTCTTGTTC 76 Yes 4.638**
