# Supplementary material for: Primer-BLAST: A tool to design target-specific primers for polymerase chain reaction
Source: BMC Bioinformatics. 2012 Jun 18;13:134. doi: 10.1186/1471-2105-13-134 (PMC3412702; doi:10.1186/1471-2105-13-134)
Supplement: Additional file 2 — PrimerPairsFromPRIMEGENS.doc Primer pairs generated from PRIMEGENS. Twenty-four randomly selected template sequences from NCBI Refseq mRNA database are used to generate target-specific primers by PRIMEGENS (Arabidopsis thaliana sequences were chosen since PRIMEGENS does not support transcript database for human). The database “Arabidopsis TAIR9 cDNA” was selected. Default values were used for all other options. The underlined pairs are used as example cases in Figure 5. [file 1471-2105-13-134-S2.doc]

>gi|186507004|ref|NM_129656.3| Arabidopsis thaliana ubiquitin carboxyl-terminal hydrolase 5 (UBP5) mRNA, complete cds

Arabidopsis thaliana ubiquitin carboxyl-terminal hydrolase 5 (UBP5) mRNA, complete cds

TCTGTTTCAGAGAGAAAAAAAAGAGGTTCCGACTTTTTGTTAGCGAGCGGCTCACTCTACTTTGTCTCCCCCAAAAAAGCCGTTAGGGTTTATCATATCCACCGCCTTGCTTTACTCTCGCCGGAGTCGTACGGCGAAGCACGGTTGTTCGGCGTTGTCTCAGCTTTAGATTCTTCTCTTCATAGCTAGAATTCGCGAAAGCTGACTTTAGCTGAAACGAATTTCTCCTAATTTTCGAGATATGGCGGAGGTATCGATGGGAAGCAGCAGTAGCAGCACGGATCTGTCTCCCGAGGAAGAGCGAGTTTTCATCAGAGACATTGCTATTGCTGCTGAGGCTAATAGTAAAGAAGGCGATACATTTTATCTCATCACTCAGAGATGGTGGCAAGAATGGATTGAGTATGTGAATCAAGACCAACCATGTAACACAAATGATGGGTCTTCACTGTCAGAGCATTGCGACTCTCCTGGTTCAAGCACTCTAAAGAAGCCTTCTAGGATTGATAACTCTGATTTGATCTATGATTCTTCGCTGGAGGACCCGAGTAATACCAGTGAGATTATTGAAACACTTCAGGAAGGTCGTGATTATGTTTTGCTCCCTCAAGAAGTGTGGAACCAATTGCGTTCATGGTACGGGGGTGGTCCAACTCTGGCACGGAGAGTCATTAGCTCAGGTCTCTCTCAAACAGAATTGGCTGTAGAGGTTTACCCTTTACGTCTTCAGCTACTTCTGATGCCAAAAAGTGACCATAGTGCCATAAGAATTAGCAAAAAGGAAACTATCAGAGAGCTCCATCGAAGAGCCTGTGAAATTTTTGACCTCGACTCAGAGCATGTACGCATTTGGGATTACTACGGCCACCAAAAGTATTCTTTGATGAATGACTTAGATAAGACACTGGACGATGCTAACCTACAAATGGATCAGGATATTTTGGTGGAGGTGCTTGACATCAATGGTACATTATCAAGTGCTCACATTCAATCTGCTCAAGAGAACGGATTGGTGGATGGAGACTCTACTTCTATTCTCATTGAGCCTTCTAAATCGAGCTTGGCTGCCGCAGGAGGGTTCTCTTCAAGCAGGAATGCATTCAGAACTGGTAGTGTAGAAGTTTCACAATCTTTTGATAACACATACAGTAGCACTGGCGTCACCACTAGGGGATCCACAGCTGGCTTGACAGGGCTGCTAAATCTTGGGAATACTTGTTTCATGAATAGTGCGATTCAGTGTCTGGTTCATACACCTGAGTTTGCTAGTTACTTTCAAGAAGATTATCATCAAGAAATAAATTGGCAAAACCCTCTTGGAATGGTGGGGGAATTAGCTCTCGCATTTGGTGACTTGCTCAGGAAGTTATGGGCTCCTGGGCGGACACCAATTGCACCTCGTCCATTCAAAGCTAAACTTGCTCGCTTTGCTCCTCAATTTAGTGGGTACAATCAACATGATTCGCAGGAGCTTTTAGCGTTTCTGCTGGATGGTCTTCACGAGGACCTAAATCGTGTTAAACACAAACCATATATAAATTCTCGAGACGCAGATGGGCGCCCTGATGAAGAAGTTGCTGATGAGTTCTGGAAAAATCATATTGCTCGTAATGACTCAATAATAGTTGATGTTTGTCAGGGCCAATATAAGTCAACATTGGTGTGTCCAATCTGCAACAAGGTTTCTGTAACGTTTGATCCCTTCATGTACCTGTCTTTGCCGCTGCAGTTCAACACAACACGAGCAATAACAGTCACAGTTTTTTCTTGTGATAAAACTGCCTTACCTTCCACTATCACAGTCAATGTTTCAAAGCAGGGACGTTGCAGAGACTTGATCCAGGCATTAACAAATGCTTGTTCCTTGAAGCAAAGTGAAGAGCTGAAACTTGCAGAGATTCGGAATAATTTTATCCATAGATTATTTGAAGACCCTTTAATTCCGTTGTCAAGTATCAAAGATGATGACCACCTTGCTGCATACAAACTTTCAAAGTCTTCGGAGAATACGACTTTGCTTAGACTAGTACTCCGCCGCAGAGATCAGAAAGCTGGGGAACGTGAAAGTACAGTACAATTGAAACCCTGTGGAACACCTCTTCTCTCATCAGCTTCATGCGGAGATGCACTCACCAAAGGAAAAATCCATTGCCTCGTCCAGAATATGCTTTCACCTTTTCGGAGGGAAGAATCTGTGGGCAAAAAAGGAAATTCTGATTCTAGCATCCCCGAGAGAAGGTCAGCTCGATTTAATAATACTGAGGAAGAAGATAAAGTTGGTGGACTGAAAAAAGCCAAGAAAAGCAACTCCTCCGACTTGGGTGCTTCTAAATTATCCCTGCAACTCATTGATGAAGACAACAAAACGATTAATCTGCCAGACAATGAAGCAGAAGCTATGAAATTACCGTCATCAGCTACTGTAACTATATATTTGGATTGGACACCAGAACTCTCTGGTATGTATGATATAACTTGCCTGGAGAGTTTGCCTGAAGTACTCAAATACGGACCTACTACCAAGAAGGCTCGTTCAGAACCTCTTTCTTTATATGCATGTTTGGAAGCGTTTCTACGCGAAGAGCCTTTGGTACCTGACGAAATGTGGTTCTGTCCACAATGCAATGAAAGAAGGCAGGCAAGCAAAAAGCTTGATCTGTGGAGGCTTCCTGAGGTCTTAGTCATACATCTAAAGAGATTCTCATACAGCAGGTCCATGAAGCATAAGTTGGAAACATTTGTTAACTTTCCCATTCATGACTTAGATCTAACAAAGTATGTAGCCAACAAAAACCTATCTCAACCTCAACTATATGAGCTCTATGCCTTGACCAACCATTATGGTGGTATGGGCAGCGGACACTACACTGCACATATCAAGCTTTTGGACGACAGTAGGTGGTATAACTTCGATGATAGCCATATATCACACATAAACGAAGATGATGTGAAGTCAGGTGCTGCATATGTGCTCTTCTACAGGAGGAAGTCTGATGCAGGCGGAAAAATGACATAAGTCTGCTCAAAACGACAATCTCCGAGGTTCCAGTAGTAATCTCTACACAGCTGTCTGCAACTTGGAACCCATAAGAGTGACAATTAGGTTTAGATCTATTGTAAATTAGTGGAGTTGGGTTCTGAGTAGTTTAATTAACAGAGATGAATATTGTCTCTCCTTCTCTGTCAGTTTCTGTATAGATTGTGTTTGGCATAATAGCAGTACTGTTATTGTCACTGACACATAATCACAAGACTCAGTCTAAATATGTAGTAGACAAGGTTTTTTATCTAGTTTGCCCAAAAACAAAAAGACAAGTTTCT

1) CTGCCAGACAATGAAGCAGA [ 2374] TCTGAACGAGCCTTCTTGGT [ 2537] psize 164 hbrdn 1

2) TCTGCCAGACAATGAAGCAG [ 2373] TCTGAACGAGCCTTCTTGGT [ 2537] psize 165 hbrdn 1

3) AGCTCTCGCATTTGGTGACT [ 1335] CCAGCAGAAACGCTAAAAGC [ 1489] psize 155 hbrdn 1

4) ACCAAGAAGGCTCGTTCAGA [ 2518] AGGAAGCCTCCACAGATCAA [ 2670] psize 153 hbrdn 1

5) GGATTACTACGGCCACCAAA [ 852] CATCCACCAATCCGTTCTCT [ 1018] psize 167 hbrdn 1

6) CCTTTTCGGAGGGAAGAATC [ 2173] TTTAGAAGCACCCAAGTCGG [ 2331] psize 159 hbrdn 1

7) AGCTCTCGCATTTGGTGACT [ 1335] AAGACCATCCAGCAGAAACG [ 1497] psize 163 hbrdn 1

8) CGTTTCTGCTGGATGGTCTT [ 1478] GGCCCTGACAAACATCAACT [ 1642] psize 165 hbrdn 1

9) AATGGTGGGGGAATTAGCTC [ 1320] CCAGCAGAAACGCTAAAAGC [ 1489] psize 170 hbrdn 1

10) ATGGTGGGGGAATTAGCTCT [ 1321] CCAGCAGAAACGCTAAAAGC [ 1489] psize 169 hbrdn 1

>gi|186507018|ref|NM_129665.4| Arabidopsis thaliana chaperone protein dnaJ 72 (AT2G41000) mRNA, complete cds

Arabidopsis thaliana chaperone protein dnaJ 72 (AT2G41000) mRNA, complete cds

GAGGAAGCGGCCATTGAAGCGCCACGAAGTCTCATTCTTCTTTCTCCAATAATAACAGAGAGCGATTCTTTTTACTATTCCAATTTCATTTTCAATGTTACCAACACGAGCTCAGATTTTCATACACCAATTTTTCTATATGCATCGTTATACACAGAAGCAGTCGCGGATCTCAATTTCAGGTGTTTGCGATTCCGATTTAGGTTTGATCGGTTGAAATTTTTTCCGATGGTGGATCATTACCAAGTTCTAGGCGTTACGAGAAACGCGACGAAGAAGGAGGTTAAAGATGCGTTTAGGAGATTGGCGATTAAGTATCATCCGGATAAGCACGCTCAGTCTCCGGAGCATGTTCGTCATAACGCCACCGTGCGGTTTAAGCTTGTATCGGAGGCGTACGAAGTTTTGAATGATGATCTGAAACGCGCGTCTTATAACGCTGGTAGCGATTCTGATTGCTTTCGCCGTACGAGCGGTTCGTATAGTAATCCGTATGGAAATCGTGGTGGCAGAGCGCAAGGTTCTGGTTACGGTTATGGTTATGGTTACTCTACGAGGAATCGTCAGGCCTCTAGCTTCAGCAGCGGGTTTGATTCGACGTTTCGTTATTTGACTACGCGAGCGTTTCTATTGAATCTCGCATTAGCTGGTGGTCTGTACTTTGCGTTTACTGCGATTGATACAAGCGGAGAAACACTATGGAAAATGCGTAACTCAGGGAAATCATTCGAAGAAGCAATGGAATCAATCGAGAAATCAAAGTCACATAAAGATGAAGGGTGAAAGAGAAGAATCAAAGGTACTTCTAAAGACTTTGACTTTTTAAGCTGCTTCTGTGGGCCTGGTGGGTTTGTGTGGGTCATTGCTAGGGACTTTTATAGATTTCACTCTCTTAGACAGGAGATGTGAAAATGTAAGTAAATTTATCAAAAATGAACCATGAGAAGTTGAAAACCCTAATTTATCATGAAGTGTAATAGATAGGTTTTTATGTTCTTGTAACAAATATTCATCTCTCCAGATAATAATCGATTATGAGAGAAGTCCTAGTACACGCAATTGTATTTGAATGCATAATGGGCAAGCAGAGATGGGATGTTGTAATAAAACAAAAACTTATTTTGGCTTTGTATCAGTAATGGAGACATTATTGTATTAGTGGTCGTAACGTA

1) GGAAAATGCGTAACTCAGGG [ 700] CCTAGCAATGACCCACACAA [ 869] psize 170 hbrdn 1

2) GGAAAATGCGTAACTCAGGG [ 700] AATGACCCACACAAACCCAC [ 863] psize 164 hbrdn 1

3) GGAAAATGCGTAACTCAGGG [ 700] AGCAATGACCCACACAAACC [ 866] psize 167 hbrdn 1

4) GCGATTGATACAAGCGGAGA [ 672] GCCCACAGAAGCAGCTTAAA [ 840] psize 169 hbrdn 1

5) TAAGCTTGTATCGGAGGCGT [ 377] TAACCAGAACCTTGCGCTCT [ 529] psize 153 hbrdn 2

6) AAGCTTGTATCGGAGGCGTA [ 378] TAACCAGAACCTTGCGCTCT [ 529] psize 152 hbrdn 2

7) TTAAGCTTGTATCGGAGGCG [ 376] TAACCAGAACCTTGCGCTCT [ 529] psize 154 hbrdn 2

8) GATGCGTTTAGGAGATTGGC [ 288] ATCAGAATCGCTACCAGCGT [ 455] psize 168 hbrdn 2

9) AGAGCGCAAGGTTCTGGTTA [ 510] ACGCAAAGTACAGACCACCA [ 666] psize 157 hbrdn 2

10) AAGCTTGTATCGGAGGCGTA [ 378] AACCGTAACCAGAACCTTGC [ 534] psize 157 hbrdn 2

>gi|186507036|ref|NM_129671.3| Arabidopsis thaliana RNA recognition motif-containing protein (AT2G41060) mRNA, complete cds

Arabidopsis thaliana RNA recognition motif-containing protein (AT2G41060) mRNA, complete cds

AAGAATTGTCTTCACGCTTCCGCGAATCGTTAAGCTTCACAATTTCAAGCGAAGAGGATCAAAAAATCAATTTTTTTGGAGAAGAACCAGAAATCACAAAACCCTAGAGGGCTCTCTTCAAATCAACCATTAAAACCGCCAATTCCGTTATGACAAAGAAGAGAAAGCTCGAATCTGAATCCAACGAAACGTCAGAGCCGACGGAGAAGCAGCAGCAGCAATGTGAAAAAGAGGATCCGGAAATCAGAAATGTTGATAATCAAAGAGACGACGACGAACAAGTAGTAGAGCAAGACACACTAAAGGAGATGCACGAAGAGGAAGCTAAAGGTGAAGATAACATAGAAGCGGAGACGTCGTCCGGATCTGGGAATCAAGGAAATGAGGATGATGACGAAGAAGAACCTATTGAGGATCTATTGGAACCGTTTTCAAAGGATCAACTTTTGATTCTTCTCAAGGAAGCTGCAGAGAGACATCGCGATGTAGCTAATCGAATCCGGATTGTGGCGGATGAAGATCTTGTTCATCGTAAGATCTTCGTTCACGGGCTTGGATGGGATACTAAAGCTGATTCACTTATCGACGCTTTTAAACAGTACGGAGAGATTGAAGATTGCAAATGTGTGGTTGATAAGGTATCTGGGCAATCTAAAGGTTATGGCTTTATCCTCTTTAAGTCAAGGTCTGGTGCTCGTAACGCTCTTAAGCAGCCTCAGAAGAAGATTGGAACTCGTATGACTGCGTGCCAGCTGGCGTCTATAGGACCTGTTCAGGGGAACCCTGTTGTGGCTCCTGCTCAGCATTTCAATCCTGAGAATGTTCAGAGGAAGATTTATGTCAGTAACGTTAGTGCAGACATTGATCCGCAGAAGTTGCTGGAGTTTTTCTCAAGGTTTGGGGAGATAGAAGAAGGTCCTTTGGGGCTTGATAAAGCTACTGGGAGACCTAAAGGTTTCGCTTTGTTTGTCTATAGATCCCTAGAAAGTGCCAAGAAGGCATTGGAGGAGCCACACAAGACTTTTGAAGGCCATGTCTTGCATTGCCACAAAGCAAATGATGGGCCAAAACAGGTTAAGCAACATCAACATAACCATAACTCTCACAATCAAAATTCCCGTTACCAAAGGAACGACAACAATGGTTATGGTGCCCCTGGAGGCCATGGACATTTCATAGCTGGTAATAACCAAGCTGTGCAGGCGTTTAATCCGGCCATTGGCCAGGCCCTCACAGCTTTGCTGGCATCTCAGGGTGCTGGGTTGGGTTTAAACCAAGCATTTGGGCAGGCTTTGTTGGGGACATTAGGGACAGCTAGCCCAGGAGCTGTAGGTGGAATGCCAAGTGGCTATGGTACTCAAGCAAATATCTCACCTGGGGTCTATCCTGGGTACGGTGCTCAAGCCGGGTACCAGGGCGGTTATCAGACTCAGCAACCTGGTCAGGGCGGTGCGGGAAGAGGGCAGCATGGTGCTGGGTATGGTGGTCCTTACATGGGTCGTTAGATTAGCCACTCAGGTAATTTAAGAGCAAGCTGAACGGTCAACACTTACGCCCAACAAAAATCTATGGGGTACCAAATATTTGAATGCCACCTATCTGCTTTTTCTTTTATATATACTGAAAAGTGAAAAGGGATGAATCTATGAATTGGTAGCTTTATAACTAAAGAACGAATTAAGCAAAAGTTGTTTTCTTGTTTAACTTAGCTAGGCATCTACCTGAATCCAAAAGAGCAACTTGTTTTGTTTTGTACTAATAAGTATCAACAAGTCTTGACCTGCACA

1) TTGGAACTCGTATGACTGCG [ 726] AAACTCCAGCAACTTCTGCG [ 886] psize 161 hbrdn 2

2) AATAACCAAGCTGTGCAGGC [ 1184] CACTTGGCATTCCACCTACA [ 1346] psize 163 hbrdn 2

3) GCTCGAATCTGAATCCAACG [ 166] TTCCTCTTCGTGCATCTCCT [ 322] psize 157 hbrdn 2

4) GCTCGAATCTGAATCCAACG [ 166] TCCTCTTCGTGCATCTCCTT [ 321] psize 156 hbrdn 2

5) CGCAGAAGTTGCTGGAGTTT [ 867] TCTTGTGTGGCTCCTCCAAT [ 1019] psize 153 hbrdn 2

6) TGGAACTCGTATGACTGCGT [ 727] AAACTCCAGCAACTTCTGCG [ 886] psize 160 hbrdn 2

7) CAGACATTGATCCGCAGAAG [ 855] TCTTGTGTGGCTCCTCCAAT [ 1019] psize 165 hbrdn 2

8) GCTCGAATCTGAATCCAACG [ 166] CCTCTTCGTGCATCTCCTTT [ 320] psize 155 hbrdn 2

9) GGGCTTGGATGGGATACTAA [ 548] TAAGAGCGTTACGAGCACCA [ 707] psize 160 hbrdn 2

10) GCTCGAATCTGAATCCAACG [ 166] AGCTTCCTCTTCGTGCATCT [ 325] psize 160 hbrdn 2

>gi|186507055|ref|NM_129675.4| Arabidopsis thaliana calmodulin-like protein 12 (TCH3) mRNA, complete cds

Arabidopsis thaliana calmodulin-like protein 12 (TCH3) mRNA, complete cds

GTAGTACAAATTGTCTCACAAACTCTTCTTTCAGTCATCACAGAAAACAAAAAAAACAATGGCGGATAAGCTCACTGACGATCAGATTACAGAATACAGGGAATCTTTCAGGTTATTCGACAAGAATGGTGATGGTTCCATTACGAAAAAGGAGCTCGGTACCATGATGCGTTCAATCGGTGAAAAACCGACAAAAGCTGATCTTCAGGACTTGATGAACGAAGCGGATTTAGATGGTGATGGAACCATCGATTTCCCTGAGTTCTTGTGCGTAATGGCTAAGAATCAAGGTCATGATCAAGCGCCGCGTCACACTAAAAAAACAATGGCGGATAAGCTCACTGACGATCAGATTACAGAGTACAGGGAATCTTTCAGGTTATTCGACAAGAATGGTGATGGTTCCATTACGAAAAAGGAGCTCCGTACCGTGATGTTTTCCCTCGGTAAAAACCGGACAAAAGCTGATCTTCAGGACATGATGAACGAAGTGGATTTAGATGGTGATGGAACCATCGATTTCCCTGAGTTCTTGTACCTAATGGCTAAGAATCAAGGTCATGATCAAGCGCCGCGTCACACTAAAAAAACAATGGTGGATTATCAGCTCACTGACGATCAGATCTTAGAATTCAGGGAAGCCTTCCGCGTATTCGACAAGAATGGTGATGGTTACATTACCGTGAATGAGCTCCGTACTACTATGCGCTCCCTTGGTGAAACCCAAACAAAAGCTGAGCTCCAGGACATGATCAACGAAGCGGATGCAGATGGTGACGGAACCATCAGTTTCTCTGAGTTTGTGTGTGTAATGACTGGTAAAATGATTGACACTCAGTCTAAGAAAGAAACGTACAGAGTTGTGAATCAAGGTCAGGGTCAAGTGCAGCGTCACACTAGAAATGACAGAGCTGGTGGCACCAATTGGGAGAGGGACATAGCGGTCGGGGTTGCCAGCAATATCATCGCTTCGCCAATTTCCGACTTCATGAAAGATAGGTTTAAAGATTTGTTCGAAGCGCTGTTATCTTGAAATGACACGTCAGTAACTTTATGCCAATAGGGTCTGACAATTATGTTAGATCTCTCTCAAAAGGCGTTTCATCTAATGACAATAATTTCTATGTAATAAATTTATTGCATGTGTTAGTGTGTTACCATCTTCAGTTGTGTGCAATTTATTAAAATGAGTTTGTAATATGGTTTCAGTATCGAATATAATTGGGGTATTAATGTA

1) CAATGGCGGATAAGCTCACT [ 56] CGCTTCGTTCATCAAGTCCT [ 225] psize 170 hbrdn 2

2) AATGGCGGATAAGCTCACTG [ 57] CGCTTCGTTCATCAAGTCCT [ 225] psize 169 hbrdn 2

3) ACAATGGCGGATAAGCTCAC [ 55] GCTTCGTTCATCAAGTCCTG [ 224] psize 170 hbrdn 2

4) CAATGGCGGATAAGCTCACT [ 56] GCTTCGTTCATCAAGTCCTG [ 224] psize 169 hbrdn 2

5) AATGGCGGATAAGCTCACTG [ 57] GCTTCGTTCATCAAGTCCTG [ 224] psize 168 hbrdn 2

6) CGGATAAGCTCACTGACGAT [ 62] CGCTTCGTTCATCAAGTCCT [ 225] psize 164 hbrdn 2

7) AAAAGCTGAGCTCCAGGACA [ 729] TAGTGTGACGCTGCACTTGA [ 898] psize 170 hbrdn 3

8) CTCCAGGACATGATCAACGA [ 739] TAGTGTGACGCTGCACTTGA [ 898] psize 160 hbrdn 3

9) CCAGGACATGATCAACGAAG [ 741] TAGTGTGACGCTGCACTTGA [ 898] psize 158 hbrdn 3

10) AAAGCTGAGCTCCAGGACAT [ 730] TAGTGTGACGCTGCACTTGA [ 898] psize 169 hbrdn 3

>gi|186507058|ref|NM_180012.2| Arabidopsis thaliana calmodulin-like protein 12 (TCH3) mRNA, complete cds

Arabidopsis thaliana calmodulin-like protein 12 (TCH3) mRNA, complete cds

GGGAATCTTTCAGGTTATTCGACAAGAATGGTGATGGTTCCATTACGAAAAAGGAGCTCCGTACCGTGATGTTTTCCCTCGGTAAAAACCGGACAAAAGCTGATCTTCAGGACATGATGAACGAAGTGGATTTAGATGGTGATGGAACCATCGATTTCCCTGAGTTCTTGTACCTAATGGCTAAGAATCAAGGTCATGATCAAGCGCCGCGTCACACTAAAAAAACAATGGTGGATTATCAGCTCACTGACGATCAGATCTTAGAATTCAGGGAAGCCTTCCGCGTATTCGACAAGAATGGTGATGGTTACATTACCGTGAATGAGCTCCGTACTACTATGCGCTCCCTTGGTGAAACCCAAACAAAAGCTGAGCTCCAGGACATGATCAACGAAGCGGATGCAGATGGTGACGGAACCATCAGTTTCTCTGAGTTTGTGTGTGTAATGACTGGTAAAATGATTGACACTCAGTCTAAGAAAGAAACGTACAGAGTTGTGAATCAAGGTCAGGGTCAAGTGCAGCGTCACACTAGAAATGACAGAGCTGGTGGCACCAATTGGGAGAGGGACATAGCGGTCGGGGTTGCCAGCAATATCATCGCTTCGCCAATTTCCGACTTCATGAAAGATAGGTTTAAAGATTTGTTCGAAGCGCTGTTATCTTGAAATGACACGTCAGTAACTTTATGCCAATAGGGTCTGACAATTATGTTAGATCTCTCTCAAAAGGCGTTTCATCTAATGACAATAATTTCTATGTAATAAATTTATTGCATGTGTTAGTGTGTTACCATCTTCAGTTGTGTGCAATTTATTAAAATGAGTTTGTAATATGGTTTCAGTATCGAATATAATTGGGGTATTAATG

1) AAAAGCTGAGCTCCAGGACA [ 364] TAGTGTGACGCTGCACTTGA [ 533] psize 170 hbrdn 3

2) CTCCAGGACATGATCAACGA [ 374] TAGTGTGACGCTGCACTTGA [ 533] psize 160 hbrdn 3

3) CCAGGACATGATCAACGAAG [ 376] TAGTGTGACGCTGCACTTGA [ 533] psize 158 hbrdn 3

4) AAAGCTGAGCTCCAGGACAT [ 365] TAGTGTGACGCTGCACTTGA [ 533] psize 169 hbrdn 3

5) ATGGTGACGGAACCATCAGT [ 405] TGCCACCAGCTCTGTCATTT [ 554] psize 150 hbrdn 3

6) CTCCAGGACATGATCAACGA [ 374] GTCATTTCTAGTGTGACGCTGC [ 541] psize 168 hbrdn 3

7) AAAGCTGAGCTCCAGGACAT [ 365] GACCCTGACCTTGATTCACA [ 515] psize 151 hbrdn 3

8) AAAGCTGAGCTCCAGGACAT [ 365] TGACCCTGACCTTGATTCAC [ 516] psize 152 hbrdn 3

9) CAGGACATGATCAACGAAGC [ 377] GTCATTTCTAGTGTGACGCTGC [ 541] psize 165 hbrdn 3

10) CCAGGACATGATCAACGAAG [ 376] GTCATTTCTAGTGTGACGCTGC [ 541] psize 166 hbrdn 3

>gi|186507073|ref|NM_129680.3| Arabidopsis thaliana uncharacterized protein (AT2G41150) mRNA, complete cds

Arabidopsis thaliana uncharacterized protein (AT2G41150) mRNA, complete cds

ATTAATGGCGACAATGAGCAAACCCCATAAGTTAAAAGCCACTCCAGGATCTCAAAGACTTGTTCTGTTATGTATAGTCGCAGTTGCATTTCTCCTCCTTTTCACTTCGGTGATCTCCACCGGCGGATTGGCTTTACCGTATCGGACAACCCTAATTGGTTATTTTGTGAGGTCAACTCGAAACAAGACACAGCATAGTTTGTCGGACAAGTACTTGTACTGGGGAAACAGAATCGATTGTCCTGGTAAGAACTGTGAGACCTGTGCCGGTTTGGGTCACCAAGAATCTAGCCTTAGATGTGCCCTTGAAGAAGCCATGTTTCTGAACAGGACTTTTGTAATGCCATCTCGGATGTGCATCAATCCAATACATAACAAGAAGGGTATACTTAATCGATCCAACAATGAAACTAGAGAGGAAAGTTGGGAAGTGAGCTCTTGTGCAATGGAATCATTGTATGATATTGATCTCATCTCTGAGAAAATACCTGTGATCTTGGATGACTCGGAAACATGGCACATAATGCTATCGACGAGTATGAAATTGAAAGAACGTGGGAGTGCGCATGTATATGGGGCAAACAGGCATGAGCTAAATGACTCTAGCGACTTTACAAATCTTTTGCTCATTAACCGAACCGCAAGCCCCCTTGCATGGTTTGTTGAATGCAAGGATCGAGGTAATCGTAGCGACGTCATGCTTCCTTATTCATTTCTCCAGACTATGGCAGCATCAAGATTGAGAGATGCTGCAGAAAAGATAAAAGCAAAACTTGGTGATTACGATGCAATCCATGTTCGTCGAGGTGACAAACTGAAAACAAGAAAAGACAGATTTCGCGTGGAAAGAAGCCAGTTTCCACATTTAGATAGAGACACACGGCCAGAATTCATCATTGGCAGAATTCAGAAACAAATCCCACCAGGACGGACTCTTTTTATCGGTTCTAATGAAAGAACCCCTGATTTCTTTTCACCTCTAGCTATCAGATACAAAGTGGCGTATTCATCGAATTTTAGTGAGATTTTGGATCCGATCATCGAGAACAATTACCAGTTGTTCATGGTGGAGAGGTTGATAATGATGGGTGCAAAGACATTCTTCAAAACATTTAGAGAGTACGAAACCGATCTCACTTTGACTGATGATCCGAAAAAGAACAAGAACTGGGAAATACCAGTTTACACCATGGATGAAGGCAAAGAAGCAGCAAGCTAAACTATCTATGATCTCACTAGGCACTAGCAAAGTCTACACAACGATTCAGACAAGAGTCTCATTACTCTTCAAATACTTGGAGTTAAAAGCCTAATCTTATCTGAAGATTCTATTGCATAGGAACTCTGTGTATGTGTAACCAGAGTCTCTAACAGAGACTAACAGACTAGATTTGCTACTTGGCTGACTCATTTTTGTGTAAATCAATCTGTTTATCTTCACTTCAATGTTCATTGCACCAGAAATGGTTTTTTAAAAACATTCGAGTTACATGGTATGGTCGTTCGTCTTTTAAGCAAGTTTCTGCAGCCACTTTCGATATCCTAATCCAATATAAGTGAAATTTCAT

1) GCCTTAGATGTGCCCTTGAA [ 290] TCCATTGCACAAGAGCTCAC [ 449] psize 160 hbrdn 2

2) TGTTCATGGTGGAGAGGTTG [ 1058] CTGCTTCTTTGCCTTCATCC [ 1210] psize 153 hbrdn 2

3) CCAGTTGTTCATGGTGGAGA [ 1053] CTGCTTCTTTGCCTTCATCC [ 1210] psize 158 hbrdn 2

4) AGATGTGCCCTTGAAGAAGC [ 295] TCCATTGCACAAGAGCTCAC [ 449] psize 155 hbrdn 2

5) TGTTCATGGTGGAGAGGTTG [ 1058] GCTGCTTCTTTGCCTTCATC [ 1211] psize 154 hbrdn 2

6) CCAGTTGTTCATGGTGGAGA [ 1053] GCTGCTTCTTTGCCTTCATC [ 1211] psize 159 hbrdn 2

7) GTGAGCTCTTGTGCAATGGA [ 430] TGCCTGTTTGCCCCATATAC [ 587] psize 158 hbrdn 2

8) GTGAGCTCTTGTGCAATGGA [ 430] GCCTGTTTGCCCCATATACA [ 586] psize 157 hbrdn 2

9) TGTTCATGGTGGAGAGGTTG [ 1058] CTTGCTGCTTCTTTGCCTTC [ 1214] psize 157 hbrdn 2

10) CCAGTTGTTCATGGTGGAGA [ 1053] CTTGCTGCTTCTTTGCCTTC [ 1214] psize 162 hbrdn 2

>gi|186507108|ref|NM_129693.3| Arabidopsis thaliana strictosidine synthase-like 2 (SSL2) mRNA, complete cds

Arabidopsis thaliana strictosidine synthase-like 2 (SSL2) mRNA, complete cds

AAAACAAAAAAAAAGAGGAAGCAATTACTATAGGGGAAAAAAAAATCTGAACAAAATGATGAAACTCCTCTTGGTGGTGGCAACTTCTGTTGCCCTTATCTTTAGCGTCACTGATTTATCCGGTGAGGGACCAAAACATGGAGGAGAATCCATGTTGACAGTCCAAATCCCTGATTTCCGTCTGATTCCTACCACCGGAGCTTTGGGACCGGAGAGTTTTGTCTTTGATTTTTTCGGCGATGGTCCTTACACCGGTTTATCTGACGGTCGAATTGTTAAGTGGCTAGCTAATGAGAGCCGTTGGATCGATTTCGCCGTCACCACCTCCGCAAGAGAAGGTTGTGAGGGCCCGCACGAGCACCAACGAACAGAACACGTGTGTGGACGACCATTGGGCCTTGCCTTTGACAAATCCACTGGTGACCTTTATATAGCCGATGCTTATATGGGCCTTCTTAAAGTTGGCCCAACAGGTGGTGTCGCCACTCAGGTGTTACCACGTGAGCTAAATGAGGCCCTTCGATTTACCAATTCTTTGGATATCAATCCACGGACCGGAGTTGTCTACTTTACCGACAGTAGTTCGGTTTATCAACGGAGGAACTATATAGGGGCGATGATGAGTGGGGACAAAACTGGTAGACTGATGAAGTACGACAACACGAAACAAGTGACCACTCTTCTAAGCAACTTAGCGTTTGTAAACGGTGTCGCTTTAAGCCAAAACGGTGATTACCTTCTCGTCGTCGAGACCGCCATGTGTCGTATCTTACGTTACTGGCTCAACGAAACGTCGGTAAAGTCTCAATCTCACGATAACTACGAGATTTTCGCGGAGGGGCTCCCTGGGTTTCCAGACAACATAAAGAGAAGTCCACGTGGCGGATTCTGGGTAGGCTTGAACACAAAACACTCAAAGCTGACAAAGTTCGCCATGTCGAACGCGTGGCTTGGACGTGCCGCTTTGGGCCTGCCGGTGGATTGGATGAAGGTCCATTCGGTTTGGGCCAGGTACAATGGGAACGGCATGGCCGTGAGGTTGAGCGAGGATAGTGGCGTTATATTAGAGGTATTCGAAGGTAAAAATGAAAATAAGTGGATATCTATAAGTGAGGTTGAAGAAAAAGATGGAACTCTTTGGGTTGGATCGGTGAATACTCCTTTCGCTGGCATGTATAAAATCTAAAATTGAGTAATACAAATATGTTTTTCCAACAATATGATTCATATATAGATAATTAGACGATACATCCCCCCTCCCCCTATTCTTATTTTTTAGGATAACAACTAATAAGTCCGTTTAGTACCACTTTATATTGTGCTAGGTAGCTACATGCATGCCTTTTGATAATTTAAA

1) AGCTAATGAGAGCCGTTGGA [ 285] GCCCATATAAGCATCGGCTA [ 450] psize 166 hbrdn 1

2) AGCTAATGAGAGCCGTTGGA [ 285] GAAGGCCCATATAAGCATCG [ 454] psize 170 hbrdn 1

3) ATGAGTGGGGACAAAACTGG [ 619] AAGATACGACACATGGCGGT [ 770] psize 152 hbrdn 1

4) GGACCAAAACATGGAGGAGA [ 127] GCCACTTAACAATTCGACCG [ 283] psize 157 hbrdn 1

5) ATGAGTGGGGACAAAACTGG [ 619] TAAGATACGACACATGGCGG [ 771] psize 153 hbrdn 1

6) GAGCACCAACGAACAGAACA [ 355] ATCGAAGGGCCTCATTTAGC [ 523] psize 169 hbrdn 1

7) TGAGTGGGGACAAAACTGGT [ 620] AAGATACGACACATGGCGGT [ 770] psize 151 hbrdn 1

8) TGAGTGGGGACAAAACTGGT [ 620] TAAGATACGACACATGGCGG [ 771] psize 152 hbrdn 1

9) CCTGGGTTTCCAGACAACAT [ 844] TGGACCTTCATCCAATCCAC [ 995] psize 152 hbrdn 1

10) ACCGCCATGTGTCGTATCTT [ 751] TGTGTTCAAGCCTACCCAGA [ 906] psize 156 hbrdn 1

>gi|186507156|ref|NM_129707.2| Arabidopsis thaliana uncharacterized protein (AT2G41440) mRNA, complete cds

Arabidopsis thaliana uncharacterized protein (AT2G41440) mRNA, complete cds

ATTATTTGAAATCCCGCTCTGAATTATTACAATGGCGCCAAAGAGAGCATCGCCGATGCAGTCTTCTCTGTACTCTATCGGTGATTACGAGGTTGCAGTGGATGGTAAGAATTTCACCTGTGAATCCGGACCCAAAAGCATTCAAATCAAATTTCCCAGAAACTACACAGTCAAAATCTCAGGTTCGTCTAGAATTTGGGTTCTTGCGGTTCTGTTTTTTTTCCACTCTAATTTGGTTGTTAGTCATTTTGGGCTCTATGATTTGATTATCTAATTCAATTTCATCTATTGATGTTATGCGGCTACTAGCAAAATGTGTAACAAACTCAATGTCCATAATTTTTCTTTTTGTGGTCAAACAACTCAATTTCCCTAAATTACTTTAGGAAATTTTCCATAATATTGGACCAAAAAAACTCCGTCTGTACTTTTTTTTTTTTTTTGGCAAAAAACTCCGTCTATACATGTAACAACAATCCTCGCAATTTCCAAAGTGGAAAAAAAGAAAAATTGGGTTTCGGTCGATCCGATTCGATGGGGAGAGAGGGCGATCAGATGAAGAGGAAGCGAAGAGATCTAACGAACAAGACGAGAGGGCCCTGCAATTTCTGCGAATTCTCCAGCAGCAGATATGGATGCAGCTCTGAAGATAAGCTGCAGGAGCAAGACAATGAAGCAGTGCTGCGGAAAGAAGTCGAGAGATTACGGCTTTTGATCAAGAGAATGACTGGGAAGGAAGATATTGTCAGCTTCACCGAGCTGCTAGCTCTTCAAAGTCACCTTATGGATGTCCGATCCATTGTGCTGGAGCAAAAGAAGGAGGTAGAGCTGGAAGAAGCTGAAAGGCCGCACAAACAGAAGAAGGAAGCAGAGCATGATGCTGACGGTATGAGGAGAGGGGTTTTGGTTCCTTGCAAATTCTCTGCCAGCAGCTTGGGAGAAAGACAGGAAGATCCAAATGGTGAAGCACCACTGCTGCAAATGCAGAGGGAATTCGAGCAACGCAGCTCTGAATCCTTGCAGAGCGAGTTCGACAAATTATGGCTTTTTAACGAGAGAATGAATGGTAGAGAGCTCGAGGGTATGACTTCCTTCGACCTGTCCATACTTCACATTCAGATATTACGAGCTCTACGGGCTTTGATTGACCAAAAGTTGGGACCAAGACAAGAACATAGGGCATGGCAGCGCGGAGAGCTTGTTTGCCACACCGACAAGGAATCAGTCTCTGGGTTAGCTGAGCCCGGGAGATGTTCGTCGGATAATGACAAGGATGCTGACTCTTCCTTGCAATGTAGACTTCTTCTCGTTTCAAGAAAGCTCAGAAGATTCCATAATTCTTATCAGCGGAAGAGCATGCCGGAGACCAGGTCGGTCTGCCAGAGTCGAGTGACTTCCGATTGACCTTATCTGCCCTGGTTGCTGCTTTCTCTGTTTCTATGTTGACATATCAACTTGCCACTTTATTTGTCTTAGTCTTGAAGTCTACGCATCTTGGATTGTAAACTTAGAAATACCAATTAGAGTTTAATTTATTACCAAGGTAAGATGTTGTTTTAAGACTCGAGGGGTTTCATATCTCACCATGTTAGGACTTGTGGGTTTCATAGAGCATCAATGAGCTTCTTTTATTTGTAAGAGATTCTCTAACACATGACGATCGATTACTCTATAAACTAGATAAATTAATCCGTATGTTATTCTAGTTA

1) TAAGCTGCAGGAGCAAGACA [ 650] AGCACAATGGATCGGACATC [ 805] psize 156 hbrdn 1

2) CTGCAGGAGCAAGACAATGA [ 654] AGCACAATGGATCGGACATC [ 805] psize 152 hbrdn 1

3) AGCTGCAGGAGCAAGACAAT [ 652] AGCACAATGGATCGGACATC [ 805] psize 154 hbrdn 1

4) TATTGTCAGCTTCACCGAGC [ 740] ACCAAAACCCCTCTCCTCAT [ 907] psize 168 hbrdn 1

5) TATTGTCAGCTTCACCGAGC [ 740] TCCTCATACCGTCAGCATCA [ 894] psize 155 hbrdn 1

6) AGAGGGCGATCAGATGAAGA [ 542] TCTCTCGACTTCTTTCCGCA [ 701] psize 160 hbrdn 1

7) AGAGAGGGCGATCAGATGAA [ 540] TCTCTCGACTTCTTTCCGCA [ 701] psize 162 hbrdn 1

8) AGAGGGCGATCAGATGAAGA [ 542] ATCTCTCGACTTCTTTCCGC [ 702] psize 161 hbrdn 1

9) AGAGAGGGCGATCAGATGAA [ 540] ATCTCTCGACTTCTTTCCGC [ 702] psize 163 hbrdn 1

10) GGGAGATGTTCGTCGGATAA [ 1245] GGCAGATAAGGTCAATCGGA [ 1414] psize 170 hbrdn 2

>gi|186507167|ref|NM_129708.2| Arabidopsis thaliana N-acetyltransferase (AT2G41450) mRNA, complete cds

Arabidopsis thaliana N-acetyltransferase (AT2G41450) mRNA, complete cds

AATTATTTGAAATCCCGCTCTGAATTTTTACAATGGCGCCAAAGAGAGCATCGCCGATGCAGTCTTCTCTGTACTCTATCGGTGATTACGAGGTTGCAGTGGATGGTAAGAATTTCACCTGTGAATCTGGACCCAAAAGCATTCAAATTAAATTTCCCAGAAACTACAGAGTAAAAATCTCAGAGAAGGATAAAATCGTTTCTGATTATCCGAAAGAAGACATGTTTCTTCTTCTTAATCCTCGGGATGAAGATGATTTTACCAAATCTCATCTCCAAGAAGTTTTGAAGTTATATAGCAAAGAGTTACCTGACATGAGATATGCTTCAAACACTGGGAAGCAATCTGCATTTCTTGAGAGATGTGTCTCTAAAGGAAAGTATTGTTCATTGGTTTTGAAGTCCACGCTTGGTGGAGACTCAGATGAGATCTTAGCTGCAATCACTTATCAAATAGTCCCTGCTGATACACAGTATGCTGAGATACCTCTTGCTGCTGTAACATATACACACCAGAAAAAGGGATTTGGTAAGCTCGTCTATGAAGAATTAATGAAGAGGCTTCATAGTGTTGGCATTCGAACTATATATTGTTGGGCAGACAAAGAATCTGAAGGATTCTGGCTTAAACAGGGGTTTATAAAATTAGCAGAGGTAGACCACAAAGGAAAAGCAAAGGGGTTGCACATTAAGTCTAATATTCGAAAAGCCTTGTGTTTTCCTGGCGGTTCAACTCTCATGCTTTCTCATCTGAACAAGGAATTTATTTCAAACCTTGAGATTTCCTCTTCATGGAAATATCAGTGTGAGGGATCTCCACTCTCGGCAAGAAATAATAGCACAGGTCCTGTGACTGGAGATTCAGTTAAACTGAGAGAGAGCTTTGGTGAAAGCGTATATCTTGACTGTATCTCTGGTATCAGAAGTCCAATGGACTCGATCACAGGAAAGGAAAACAACAACGTGATCTCTGATCAAGCTACCACAGCTGACAGTGAGACCAAATGTTCAACACCAGGCTTAAAGAGATCTTGGGAAGCTTCATTGTCCTCTCTACAATCTAAAAGGATTCGGGCAAACAACAATAATAACTCTGAGATAGCAAAAACAGATTTGGCTCAGAGCTCTGCAAAGCAATCGAAAGATGGTAATTCCTCCCAAGTAGATATAACCAAGGATTCCCTGCCCACAATCTGCAAAAGAAACGACGTTGAACAATGTAGAATGGCAACTGGGATAGACATGGAAGCTCGTCCAAATGGACAACACTATAGAATCTTGTTGATGGACATTTGTGATGAAAACAAGAGAGCTTGGTTAACAGAGGTAATAAGAAAACTTGGTGGTACTGTGACCTTGGATGGCACAACGAGCACGCATATTGTCACCGGAAAAGTCAGGAAAACTCTGAATCTCTGCACTGCTCTTTGCTCTGGGGCGTGGATAGTATCACCAAGTTGGTTAAAAGAAAGTGTCCGAGAAGGAAGATTTGCTAATGAAGCTTCACACATACTGCATGACGAAGACTACCAGTTGAAATATGATACTGATCTAAAAAGCACAGTTCTTAGAGCAAAAGCTAGACCAAACTCGTTGCTAAAAGGATATGACATATGCGTTGGACCTAACATTGAACTGCCTATCAAAACTTCATCTGCTATCATCAAATCTGCTGGTGGAAATGTGATAAGTGGAGTGAATAAGGTAAAGGAGGCGTCAAAAGCGATATACATAGGGTGCGAAGAAGACACAGTAGGGGCATTATTTGCAGCAAAGAAAGGAATTTGGACATTCAGCAGCGAATGGTTCATGAACTGTGTCATGAAGCAACAACTTGATCTTCAAGTTCCTCAGTTTGTTGAGTCCTTATGATGATGACTCGAACTCGAAACCTCCTATGATCTTTAGCTTGGAAAATCTCACTTATTATCTTTTTATGTTTAGAGACATTGTCGGAAGTTGAATGGATAGGCTAAGAAGCAAACTCAAGTCGCCGGTCTATTTATGTATAAAAATAAAAATGTGTTACTTGAAGGTTGGTTTAAGTTACATTTAAAAGTTTGAG

1) TGGGAAGCTTCATTGTCCTC [ 1031] GGCAGGGAATCCTTGGTTAT [ 1185] psize 155 hbrdn 2

2) CTCGTCCAAATGGACAACAC [ 1248] TTCCTGACTTTTCCGGTGAC [ 1401] psize 154 hbrdn 2

3) GACATGGAAGCTCGTCCAAA [ 1238] TTCCTGACTTTTCCGGTGAC [ 1401] psize 164 hbrdn 2

4) CATGGAAGCTCGTCCAAATG [ 1240] TTCCTGACTTTTCCGGTGAC [ 1401] psize 162 hbrdn 2

5) GCAACTGGGATAGACATGGA [ 1226] GTGACAATATGCGTGCTCGT [ 1386] psize 161 hbrdn 2

6) GCAACTGGGATAGACATGGA [ 1226] GACAATATGCGTGCTCGTTG [ 1384] psize 159 hbrdn 2

7) GGGAAGCTTCATTGTCCTCT [ 1032] GGCAGGGAATCCTTGGTTAT [ 1185] psize 154 hbrdn 2

8) TGGGAAGCTTCATTGTCCTC [ 1031] CTTTTGCAGATTGTGGGCAG [ 1200] psize 170 hbrdn 2

9) GAAAAGCAAAGGGGTTGCAC [ 666] TTCTTGCCGAGAGTGGAGAT [ 830] psize 165 hbrdn 2

10) GTCACCGGAAAAGTCAGGAA [ 1382] CAACTGGTAGTCTTCGTCATGC [ 1534] psize 153 hbrdn 2

>gi|186507201|ref|NM_129714.3| Arabidopsis thaliana cytokinin dehydrogenase 1 (CKX1) mRNA, complete cds

Arabidopsis thaliana cytokinin dehydrogenase 1 (CKX1) mRNA, complete cds

CCCTCAGAGTAGTACTCTAAACCTCAAGTTTACCTCTACTTCTCTTATACCATTCCGCCTCTTATTCTTTGCAATTTCTCTCAACAAAGTAGAAATGGGATTGACCTCATCCTTACGGTTCCATAGACAAAACAACAAGACTTTCCTCGGAATCTTCATGATCTTGGTTCTAAGCTGTATACCAGGTAGAACCAATCTTTGTTCCAATCATTCTGTTAGTACCCCAAAAGAATTACCTTCTTCAAATCCTTCAGATATTCGTTCCTCATTAGTTTCACTAGATTTGGAGGGTTATATAAGCTTCGACGATGTCCACAATGTGGCCAAGGACTTTGGCAACAGATACCAGTTACCACCTTTGGCAATTCTACATCCAAGGTCAGTTTTTGATATTTCATCGATGATGAAGCATATAGTACATCTGGGCTCCACCTCAAATCTTACAGTAGCAGCTAGAGGCCATGGTCACTCGCTTCAAGGACAAGCTCTAGCTCATCAAGGTGTTGTCATCAAAATGGAGTCACTTCGAAGTCCTGATATCAGGATTTATAAGGGGAAGCAACCATATGTTGATGTCTCAGGTGGTGAAATATGGATAAACATTCTACGCGAGACTCTAAAATACGGTCTTTCACCAAAGTCCTGGACAGACTACCTTCATTTGACCGTTGGAGGTACACTATCTAATGCTGGAATCAGCGGTCAAGCATTCAAGCATGGACCCCAAATCAACAACGTCTACCAGCTAGAGATTGTTACAGGGAAAGGAGAAGTCGTAACCTGTTCTGAGAAGCGGAATTCTGAACTTTTCTTCAGTGTTCTTGGCGGGCTTGGACAGTTTGGCATAATCACCCGGGCACGGATCTCTCTTGAACCAGCACCGCATATGGTTAAATGGATCAGGGTACTCTACTCTGACTTTTCTGCATTTTCAAGGGACCAAGAATATCTGATTTCGAAGGAGAAAACTTTTGATTACGTTGAAGGATTTGTGATAATCAATAGAACAGACCTTCTCAATAATTGGCGATCGTCATTCAGTCCCAACGATTCCACACAGGCAAGCAGATTCAAGTCAGATGGGAAAACTCTTTATTGCCTAGAAGTGGTCAAATATTTCAACCCAGAAGAAGCTAGCTCTATGGATCAGGAAACTGGCAAGTTACTTTCAGAGTTAAATTATATTCCATCCACTTTGTTTTCATCTGAAGTGCCATATATCGAGTTTCTGGATCGCGTGCATATCGCAGAGAGAAAACTAAGAGCAAAGGGTTTATGGGAGGTTCCACATCCCTGGCTGAATCTCCTGATTCCTAAGAGCAGCATATACCAATTTGCTACAGAAGTTTTCAACAACATTCTCACAAGCAACAACAACGGTCCTATCCTTATTTATCCAGTCAATCAATCCAAGTGGAAGAAACATACATCTTTGATAACTCCAAATGAAGATATATTCTATCTCGTAGCCTTTCTCCCCTCTGCAGTGCCAAATTCCTCAGGGAAAAACGATCTAGAGTACCTTTTGAAACAAAACCAAAGAGTTATGAACTTCTGCGCAGCAGCAAACCTCAACGTGAAGCAGTATTTGCCCCATTATGAAACTCAAAAAGAGTGGAAATCACACTTTGGCAAAAGATGGGAAACATTTGCACAGAGGAAACAAGCCTACGACCCTCTAGCGATTCTAGCACCTGGCCAAAGAATATTCCAAAAGACAACAGGAAAATTATCTCCCATCCAACTCGCAAAGTCAAAGGCAACAGGAAGTCCTCAAAGGTACCATTACGCATCAATACTGCCGAAACCTAGAACTGTATAAAAGTTTCCTGTGTCCGTCCTTGTAACCGCTCAGGCTAGGCAGCAAGAAAGTGAAAAATTCTTTCTTTTTTGTTTCTTTTGGTGAACAACAAATTTTACAGTCTGAAACAGATTGGGACAATTGTATAGGATCTCCATAAAAA

1) GATTCTAGCACCTGGCCAAA [ 1683] ACAAGGACGGACACAGGAAA [ 1844] psize 162 hbrdn 1

2) GGGAAACATTTGCACAGAGG [ 1640] GGTTTCGGCAGTATTGATGC [ 1808] psize 169 hbrdn 1

3) GATTCTAGCACCTGGCCAAA [ 1683] TACAAGGACGGACACAGGAA [ 1845] psize 163 hbrdn 1

4) GATTCTAGCACCTGGCCAAA [ 1683] TTACAAGGACGGACACAGGA [ 1846] psize 164 hbrdn 1

5) ATTCTAGCACCTGGCCAAAG [ 1684] ACAAGGACGGACACAGGAAA [ 1844] psize 161 hbrdn 1

6) CTAGCACCTGGCCAAAGAAT [ 1687] ACAAGGACGGACACAGGAAA [ 1844] psize 158 hbrdn 1

7) CTAATGCTGGAATCAGCGGT [ 683] TATGCCAAACTGTCCAAGCC [ 846] psize 164 hbrdn 1

8) GACCCCAAATCAACAACGTC [ 719] ATATGCGGTGCTGGTTCAAG [ 887] psize 169 hbrdn 1

9) GACCCCAAATCAACAACGTC [ 719] TGGTTCAAGAGAGATCCGTG [ 876] psize 158 hbrdn 1

10) TCTAGCACCTGGCCAAAGAA [ 1686] ACAAGGACGGACACAGGAAA [ 1844] psize 159 hbrdn 1

>gi|186507204|ref|NM_180020.3| Arabidopsis thaliana DNAJ heat shock N-terminal domain-containing protein (AT2G41520) mRNA, complete cds

Arabidopsis thaliana DNAJ heat shock N-terminal domain-containing protein (AT2G41520) mRNA, complete cds

GTTGGTAATAATAATAAGCAAATCCCAAAAACCCTAACGGTACTGTGTAGAAGCTATATACATTCCTCTTACTTCACCTCAACACGCAAGACCAGATTCGTTTCCCCGAGAGATTCCTTTCCTTAAGCCCTAAAGTTTCGATTTTTGATTTTTATTTTGAAAGAATCCAATTTTTGGATGTCTCCTGCGGCGGTGGAGATTGGGTCTCCGGTTGAAAAAGTCTCTTCTGTTCTAAACCCTATGGAAAATTTCTCTGCATTTTTTCCGGAGAGTGTTCACTCTCACGATCAACGAACCATGGGCTCTTCGTTCTCCTTCGGATTTGGAGCTGGTTCAGGACAAACCACGAAGACTCGACACAAGCCGAGGCTCGTGAAATTGAGGAAGAACGGAAGAGAGGTGAAAAAACCTTCTTTTTCCGGCGAGATCCTCTCAGGTTTCAATCCGTTTGCGCCGCCGGGTAAAGGTAGTCTTCATATGAACAGTGGGAATGATAGAGATGCTGGCCAATCACCTGGGGATAAATGTTTTGTCTTTGGAGCTAGTGGGAACAGTTCTGGTGCAAAATCTAGTCCAGGCAATGCCACAATAGCTTCTTTGTCTGATGAAGAGGAGTTTAGTACACCTATAGGGGACTCTGAGTTGGATTCGGAATCCATTAAAGAACATTCAAATGGAGTTAGGGGAAAGGTTGATAATGATCGGGAAAACATCAGACCGTGTAGTGAGAGCACAATTTTTGATGCCACAAATGGATTTAGGTTTGATGGTGGTGTTAACGGAAGCTGGAAAAGTGTTGAGAATCCAGATGCTGTCAAGGAGTTTTTTCGAAGACAGAATGGCATGGACAAGGCATCAGCGAGTAAATCTTGTAGCAAGTCTAACAGTGTACAATATGCTCATGGTAAAAATAGGGATGATCCTGAATTGAACCTGCTTGCTGACATGGAGAAGCTTAATATCAGTGATTCCAGAGTTCATGGTGGCAGCGACTATGAGGAAGCAAGCAAATCCAATAAGACACCTGTATTTACTTTTAGTAGCTTTGGAAAGGTTGATCCTATATGTAAAGAAGGTGCGGCGACATCAGAGCCATACTCGTTCAGTTCCAATGGTTTTCAGCAAAGTAATAATGCGTCGGGTGAAAACCCCACTTTTCATTCCCAGACCACCTACGGAAACAACTTAACAAACACTAGCTTTGCTACAAAAACATTCTTTGATGATTTCAAAGTACCTGAATGGGATCCTTCGTTGCTTAAGGACAGTCTGTTTCCAGAAGTAGACAGAAATCCAGTACATGCGCGAAGTAATCGTTCGTCCAAGGACAAGAGATCAAAGAAAGTTAAGGAAAAAATGAAACAGGGTGAGCCAGATCGATGCAATGGTCAAACTGCTGAGGGAATTGAGGCTCAAGAAAAACTCAATTCCCCTGGATATTGCTCACCCATGGATTATTCTCCTTATCAAGGTGACAAAACGAGCAATCAATTTCCAACAGAAACTCCCCTGGCACCATCACATTCAAGAGAACATATTGATTCCCGCTCTAGCAATGACTTTAAGGTTGCGTCAGCCAGGGATTCATCTCTCTTCACGGCAGAGGATCATGGGAGTACTTGTATTCCGAATTTTTCTTTCTCAGCGTCCACCTCTCAGGAAACAATACGACATAAAAAGCTTCAAGCTGTAAAGAAATATAGGAGGAAAGTTAACAACAGTTTGCCGAAAAGCAATCTTAATGCCACCATGCGAAACAATCAAGAGAATCAACCTGTGAATACAGGTCAAGCCAAACAGGACTCGGGCTCCACATCGATGATGCCTGATGTCTGTGAGGTTTGGCGACTAAGGGGAAATCAAGCCTACAAAAATGGTTATATGTCTAAAGCTGAGGAATGTTACACACATGGTATTAATTCTTCTCCGTCAAAAGATAATTCAGAATACTCTGTGAAGCCTCTTGCGCTTTGCTATGGTAACCGCGCCGCAGCACGGATTTCTCTTGGAAGATTGAGGGAGGCTATAAGCGACTGTGAGATGGCTGCCTCACTTGATCCTAGCTACATTAAAGCGTATATGAGAGCTGCAAATTGTCATCTTGTGCTGGGGGAACTTGGATCAGCAGTGCAGTATTTTAATAAATGCATGAAATCCACCTCTAGTGTCTGCTTGGATCGACGAACTACTATAGAAGCAGCTGAAGGTTTACAACAGGCTCAAAGGGTAGCTGACTTTACCAGCTGTGCATCCATATTTTTGGAAAAGAGAACACCTGATGGCGCATCTGACGCATTGGTTCCAATTGCCAATGCCTTATCAATTAGTTCATGCTCAGATAAATTGCTTCAAATGAAGGCCGAGGCCCTATTTATGATCCGACGATATAAAGAAGTTATTGAGCTTTGTGAGAATACCCTTCAGACTGCTGAGAGGAATTTTGTTTCAGCAGGGATTGGTGGGACGACAAATGTTAATGGATTAGGGTCTACGTATCACTCGCTAATAGTTTGGAGATGGAACAAAATTTCCAAGTCGCACTTCTACTTGGGAAACCTTGAGAAGGCCCTTGATATATTAGAAAAGTTACAGCAAGTGGAATATACCTGCAATGAAAATCAGGAAGAGTGTCGTGAGTCACCAGCTTCTTTAGTGGCCACCATTTCTGAACTCTTACGTTACAAGAACGCAGGTAATGAAGCTGTTCGGGACAGAAAGTATATGGAAGCAGTAGAGCAGTATACTGCTGCACTATCAAGAAATGTTGACTCACGCCCTTTTGCAGCAATTTGCTTCTGCAATCGTGCGGCTGCTAATCAGGCCCTAGTTCAAATTGCTGATGCAATTGCCGACTGTAGTCTTGCCATGGCTCTTGATGAAAACTACACAAAGGCAGTTTCCAGGAGAGCCACATTACATGAGATGATCAGAGACTATGATCAAGCAGCTAGTGATCTCCAGAGACTTATCAGCATTCTCGTAAAGCAAAGTGATAAAACAAAAACGCCAGAGACATCTGTCGATCGTGCAAGTAGCAGGAAAGAACTAAAGCAGGCCCGTCAACGGTTGTCTGTGATGGAAGAAAAATCTAAAGAGGGAATTCATCTGGATTTCTTCCTAATCATGGGAGTGAAGACATCTGACTCTGCTGCTGATATCAAAAAGGCATACCGCAAAGCAGCTCTTAGACATCACCCAGACAAAGCTGCACAGATTCTTGTCAGAAGCGAAAGTGAAGGACCATGGTTGAAGGAGATATTAGAAGAGGTTCACAAGGGTGCAGATAGGCTCTTCAAAATGATTGGAGAGGCATATTCAGTTCTTTCTGACCCAACTAAGAGGTCGGATTATGAACTTGAGGAAGAAATTAGAAAAGCCAGGGCATCTAGAGAAAGCTACAGGAGCAGAAAGGCTGCAGAAGCAAGTAGCCCTCCATATCAGACAAGCAGGCGATACTGGAAGGACAGTTGGAGGACAAACCAAAACACGCCTTCTTGGTGGTAGAAGGCTAAAATTCTTGGTGGCGTAGCAAAAGGAACGCAGTACTTTTTGTGGCATAAGCAAAAGGGATCCACTGGGTTCCTCCCAGGTGTTATTCCAGAGGTTGTGAAGTGTAAAGGTCCGCTACACATCAATACAGCAAATGATACCAGCTTACATTGTTTGCTTCAATGGTGAAAAGGGGAATTACTGCGAGGCTGTGACCCAACTACACAGTAACTGAAAAAAAGTTTGTTTGCTTTTACTGACTATAGTGTTTGTAAGTTGTAATACATTCAAAAGATCAAATAAATGAGAAACCTGAATTGGTCTGAACATA

1) ACCTTGAGAAGGCCCTTGAT [ 2554] TCCCGAACAGCTTCATTACC [ 2710] psize 157 hbrdn 1

2) ACCTTGAGAAGGCCCTTGAT [ 2554] CTGTCCCGAACAGCTTCATT [ 2713] psize 160 hbrdn 1

3) ACCTTGAGAAGGCCCTTGAT [ 2554] TTTCTGTCCCGAACAGCTTC [ 2716] psize 163 hbrdn 1

4) AGAAATGTTGACTCACGCCC [ 2757] TCTCCTGGAAACTGCCTTTG [ 2906] psize 150 hbrdn 1

5) ACCTTGAGAAGGCCCTTGAT [ 2554] CTTTCTGTCCCGAACAGCTT [ 2717] psize 164 hbrdn 1

6) TTCCAACAGAAACTCCCCTG [ 1492] AGGTGGACGCTGAGAAAGAA [ 1653] psize 162 hbrdn 2

7) AATGAAACAGGGTGAGCCAG [ 1355] CAGGGGAGTTTCTGTTGGAA [ 1511] psize 157 hbrdn 2

8) TACCTGAATGGGATCCTTCG [ 1234] CCCTCAGCAGTTTGACCATT [ 1402] psize 169 hbrdn 2

9) CATATCAGACAAGCAGGCGA [ 3433] TGGAATAACACCTGGGAGGA [ 3599] psize 167 hbrdn 2

10) TTCCCCTGGATATTGCTCAC [ 1427] AGAGATGAATCCCTGGCTGA [ 1591] psize 165 hbrdn 2

>gi|186507209|ref|NM_129716.3| Arabidopsis thaliana S-formylglutathione hydrolase (SFGH) mRNA, complete cds

Arabidopsis thaliana S-formylglutathione hydrolase (SFGH) mRNA, complete cds

GCCAGAAGTTTTAAGGAGATATGTTGTTGAATTATTTCTATGGGCTCTCTCGGATCCACTGTAGAAACCGATAGACCTCTCTCATTCGATCATTTCTTCTTCTTCTTCGTCTTCGCTGCAGCCTGCATCTGTTGTTTCTTCTAGACTTTGCAATCACAGAGATAGTGAGTGAGATAGAGAGAGTGATAATGGCGAGTGGACTAAGCGAGATCGGGAGCACGAAGATGTTCGATGGCTACAACAAAAGATACAAACACTTCAGTGAGACACTTGGATGTTCCATGACCTTTTCCATCTACTTCCCTCCTTCTGCTTCTTCTTCCCATAAATCTCCTGTGCTTTACTGGCTTTCTGGCCTCACCTGCACGGACGAGAACTTCATTATCAAATCAGGAGCTCAACGTGCTGCTTCTACTCACGGCATTGCTCTTGTTGCTCCAGACACTTCTCCAAGAGGACTAAATGTTGAAGGGGAGGCAGACAGTTACGACTTTGGTGTAGGAGCCGGATTCTACCTCAATGCTACTCAGGAAAAGTGGAAGAACTGGCGTATGTATGACTATGTTGTCAAAGAGTTGCCAAAACTCCTGAGTGAAAACTTTTCCCAGCTTGACACAACAAAAGCATCTATATCTGGACACTCCATGGGTGGACATGGAGCTCTTACTATATACCTGAGGAACCTCGATAAATACAAGTCTGTATCTGCGTTTGCACCAATCACGAATCCCATAAATTGTGCATGGGGACAGAAGGCATTCACCAATTATCTAGGTGACAACAAAGCTGCTTGGGAGGAATACGATGCCACTTGTCTTATTTCAAAGTACAACAATCTTTCTGCAACAATTCTAATTGATCAGGGAGAAAACGACCAGTTCTACCCTGATCAGTTATTGCCCAGCAAGTTTGAGGAGGCGTGCAAGAAAGTGAATGCACCGCTCTTATTGCGCCTCCATCCAGGATACGACCACTCCTACTATTTCATTGCCACCTTCATCGAAGACCACATTAGTCACCATGCTCAAGCCCTTGAGCTATAGCTCACTTCATCTGCTTGGAAACCGGCTTTGGGTTTGTCCAAGTATTAGTATCTCAATAAAGCAAGTGGACTTGTAATGTTTTATGTTCAATAACTCCCCTGTGTGCTCTTTTGTCTACGATAATAATAAGAAAATCATCTTTGCTTCATATCATACCTCAT

1) AATCAGGAGCTCAACGTGCT [ 387] CGCCAGTTCTTCCACTTTTC [ 549] psize 163 hbrdn 1

2) GATCAGGGAGAAAACGACCA [ 857] TGTGGTCTTCGATGAAGGTG [ 1010] psize 154 hbrdn 1

3) TGATCAGGGAGAAAACGACC [ 856] TGTGGTCTTCGATGAAGGTG [ 1010] psize 155 hbrdn 1

4) AAATCAGGAGCTCAACGTGC [ 386] CGCCAGTTCTTCCACTTTTC [ 549] psize 164 hbrdn 1

5) TCAGGGAGAAAACGACCAGT [ 859] TGTGGTCTTCGATGAAGGTG [ 1010] psize 152 hbrdn 1

6) CAAATCAGGAGCTCAACGTG [ 385] CGCCAGTTCTTCCACTTTTC [ 549] psize 165 hbrdn 1

7) CACCTTCATCGAAGACCACA [ 991] ACAAAAGAGCACACAGGGGA [ 1156] psize 166 hbrdn 1

8) CAGGGAGAAAACGACCAGTT [ 860] TGTGGTCTTCGATGAAGGTG [ 1010] psize 151 hbrdn 1

9) ATCAGGGAGAAAACGACCAG [ 858] TGTGGTCTTCGATGAAGGTG [ 1010] psize 153 hbrdn 1

10) CACTTGGATGTTCCATGACC [ 267] CAATGCCGTGAGTAGAAGCA [ 425] psize 159 hbrdn 1

>gi|186507215|ref|NM_180021.4| Arabidopsis thaliana 6-phosphogluconate dehydrogenase-like protein (GPDHC1) mRNA, complete cds

Arabidopsis thaliana 6-phosphogluconate dehydrogenase-like protein (GPDHC1) mRNA, complete cds

CTATGTCCATTCCTCTCCCTTGCACATAAGCTTTGCTCTGTCTATTTAATTTAACTCTTCTTCTGCATGAAAGTTTTATGCTTTCGATGATTCCCAAAATCTTTCTCATGCGTTTTTTACTTTCTTGATTTGGCAATCCTCATGAGCTGATTTTGATTTCTTCTTTATGGTTGCCGACACGAAAGAAGAAGCTAGGACACAAAACAACTCAAATTTTGTTTGGTGAGAAAAATCAACCAAGAAAATATTGATTCTTTTGGAGCAAGGAGTTGGTTTGATTGGTAATGCTCAAGTTGGTCTCTCTTTTTTCTTTTCAAATCCAACTTGATTTCTTTGTTCTATATGATCCAAACCCATGTAGTATAAATCTGTAGTGGATTACATAACCACCAGAAAAAATCATTTCTAGTCATTGTTCATTATTGTCTTTGGTTTGTTTGAACTTTCTTGTGTGTGTGGACCATAAGAAGAAGGAATGGTGGGAAGCATTGAGGCAAAGAGTCTGCAATCAAACGGGTCTGTTCATCATATTGGTCTTAATTTGGAGGAGAAACTTGATGAATTTCGTCGTCTTTTGGGGAAATCAGAAAAAGATCCGTTAAGGATTGTAAGTGTTGGTGCTGGTGCTTGGGGAAGTGTTTTTGCAGCACTTCTTCAAGAAAGCTATGGAGGTTTCAGGGATAAGTTTCAGATCAGGATATGGAGAAGAGCTGGGAGAGCTGTTGATAGAGAAACTGCAGAACATTTGTTTGAAGTGATCAATTCAAGGGAAGATATCTTGAGGAGATTGATAAGACGCTGTGCTTATCTGAAATATGTCGAGGCAAGGCTTGGTGATAGGACACTCTATGCTGATGAGATATTGAAAGACGGGTTTTGTCTTAACATGGTTGATACGCCGCTTTGTCCTCTTAAGGTTGTGACAAATCTGCAAGAAGCTGTGTGGGATGCTGATATTGTTGTTAATGGATTGCCTTCAACTGAAACACGTGAAGTGTTTGAAGAGATTAGTAAGTATTGGAAAGAGAGAATAACGGTTCCGATTATTATCTCTCTGTCAAAGGGTATTGAAACTGCTCTTGAACCAGTTCCACATATCATAACTCCAACAAAGATGATTCATCAAGCAACTGGTGTGCCGATTGACAATGTCCTGTATCTTGGTGGACCAAACATTGCTGCTGAAATTTACAACAAGGAATATGCCAATGCTAGAATCTGTGGAGCTGCTAAATGGAGGAAGCCACTAGCTAAGTTCTTAAGACAACCTCATTTCATTGTTTGGGACAATAGTGATCTTGTGACACATGAAGTAATGGGAGGTCTCAAGAATGTCTACGCCATTGGAGCTGGTATGGTAGCGGCGCTCACTAACGAGAGCGCTACAAGCAAGTCGGTGTATTTTGCTCATTGTACATCTGAGATGATATTTATAACTCATTTACTAGCAGAAGAGCCTGAGAAACTTGCAGGGCCTTTGCTAGCTGACACTTATGTGACCTTATTAAAAGGACGTAATGCATGGTACGGTCAAATGCTGGCAAAGGGTGAAATAAATAGAGACATGGGTGATAGCATAAGCGGCAAGGGAATGATTCAGGGTGTTTCTGCAGTGGGAGCATTTTACCAATTGCTTAGTCAATCAAGCTTAAGTATATTGCCCTCTGAAGAGAAGAAACCTGTAGCTCCGGTCGAATCATGTCCTATTTTAAAGACACTTTACAAGATACTCATCACAAGAGAACAATCAACTCAGGCCATTCTGCAAGCATTAAGGGATGAGACATTGAACGATCCCAGAGACCGTATTGAGATTGCACAGAGCCATGCATTCTACAGGCCTTCCCTTCTTGGTCAGCCTTGATTAGTCAGTCATGGCTTTTATAAGAGATTCAACATAAATTACTTCTGCAAGCTGCAGATGCTTCATTCAAAACATTATAGCTTTAATGGGAAGGGGTAGTTTCACATCTGTCATAACTTAAAATGGATTCTTGTTAAGTCTCTGAGTCAATGCAATCTTTGTACTGTATCATTTTCTTTATGATCAATGAGAGCTTTATTTGCTCCAACAATTTTAGTAAAAGAAAGAAAACACAGATTTTAGTTACT

1) CCGGTCGAATCATGTCCTAT [ 1687] AAGGCCTGTAGAATGCATGG [ 1843] psize 157 hbrdn 3

2) GCACAGAGCCATGCATTCTA [ 1816] TGTGAAACTACCCCTTCCCA [ 1969] psize 154 hbrdn 3

3) TAACGAGAGCGCTACAAGCA [ 1371] TGACCGTACCATGCATTACG [ 1532] psize 162 hbrdn 3

4) GTTTCTGCAGTGGGAGCATT [ 1603] GCAGAATGGCCTGAGTTGAT [ 1765] psize 163 hbrdn 3

5) TAGCTCCGGTCGAATCATGT [ 1682] TAGAATGCATGGCTCTGTGC [ 1835] psize 154 hbrdn 3

6) TGTAGCTCCGGTCGAATCAT [ 1680] TAGAATGCATGGCTCTGTGC [ 1835] psize 156 hbrdn 3

7) GTTTCTGCAGTGGGAGCATT [ 1603] CTTGCAGAATGGCCTGAGTT [ 1768] psize 166 hbrdn 3

8) GGAGCTGCTAAATGGAGGAA [ 1222] TGCTTGTAGCGCTCTCGTTA [ 1390] psize 169 hbrdn 3

9) TAGCTCCGGTCGAATCATGT [ 1682] AAGGCCTGTAGAATGCATGG [ 1843] psize 162 hbrdn 3

10) TGTAGCTCCGGTCGAATCAT [ 1680] AAGGCCTGTAGAATGCATGG [ 1843] psize 164 hbrdn 3

>gi|186507236|ref|NM_129725.4| Arabidopsis thaliana Nup93/Nic96 nucleoporin interacting component-containing protein (AT2G41620) mRNA, complete cds

Arabidopsis thaliana Nup93/Nic96 nucleoporin interacting component-containing protein (AT2G41620) mRNA, complete cds

AAATAAATCAAAAAGGTTTGAATCCCGACGGAAACCCTCTCTTTGCTGCTTCCCCGACTGCGTAGTAGTAGCTCGAATCCAAAATCAATGGCGAACGACCAAGAGATGAGTGGTTGGACTGATCTTCTCCATTCTTCGTCGAAGCTTCTGGAGCAAGCTGCTCCTTCTTCTCAGTTTCCTCCTCTTCAGAGAAATTTAGATCAGTTGGAAGCGTTATCGAAGAAGCTCAAGGCTAAAACCCTGAGGAACGAAGCTCCTTCTCAGTCTATTGCTGCCACTAGACTACTCGCACGGGAGGGAATCAATGCAGAGCAACTAGCTCGTGATCTTAAATCTTTCGAGCTCAAGACAACATTTGAGGATGTGTTCCCTGCTGAGGCGACGAGCGTTGAAGAATACCTGCAACAGGTTCATGAAATGGCTATGGTATCAGCTATTCAGGAAGCCCAAAAGGATAATGTTCGAAGCTTTAATGACTACATGATGAAAGTGTTGGAGGAGGATTGGAGAAAAGAAAAACGTGACTTCCTTCAGAGCCTCAGCAGAATATCAATGCTACCTAAAACAAATATGATTGATACAAGCAGAGAGGCTCATGCTGGTCAACTAGTGCCGGTGGGTTCGAGTCCTCGAGTATCCTCAACTCCAGGCAAAGAACTCGTGGCTCTGGCTAACATACCCATTCATGAGAAAAAAGCATATGTCTATGGGGAGGTTGTGAAGAAACTTAACACTTCAAGAGAACGAGGCATGCCATTTAGACCTGCTATGTGTTTTAAGGACGCTTATGATACTCTTGGTGCTGAGGTAACTCGTGGAAAATCAGTCAACATGCAGAAAATATGGCAGCTTGTTCAGGCAATAACAGGTGAAGATTCAGCAGTTCGACAGGGTGTTTCGAAGAGGATGGCCCTCGCGATAGGTGCAAGACATCATTTACAACATGGGCATGAGAAATTTATAATGGACACAATTCAAACTCACCCTACACAGGCTGCTCTTGGTGGATCTGTTGGAAATTTGCAAAGAATTCGTGCTTTCCTTCGGATTCGTTTGAGAGACTATGGAGTTTTAGATTTTGATTCAACTGATGCTCGTAGGCAGCCTCCTGTTGATACTACTTGGCAACAGATATATTTTTGCTTGCGAACTGGGTATTATGAGGAGGCTAGGGAAATTGCTCGGTCAACACGGTCATCTCAGCAATTTGCACCACTGCTTACAGAGTGGATTACCACAGATGGTATGGTGGCTGCAGAGTCTGCAGCTATTGCATCAGAAGAATGTGAGAAAATGCTAAGGATGGGCGATCGGTTGGGTCGAACAGCTTATGACAAGAAAAAACTCCTACTCTACACTATCATTTCTGGTTCCCGTAGGCAAATTGAGCGCATACTGAGGGATCTATCTACACTCTTTAACACAATTGAAGATTTCTTATGGTTCAAATTGTCATGCATAAGAGACGTCACTGGTGGATCCTCATCTGTGGTCCTGAATGATGGCCTTGCGCCATACAGTTTGGATGATCTTCAGGCTTATCTCAATAAATTTGAGCCTTCATACTACACAAAGAATGGAAAAGACCCCTTGGTATATCCATATGTTTTGCTTCTCAGTGTTCAGTTGCTACCGGCCATCATGCACTTATCCAAAGAAGCAGGAGACGGAGGGTATAATATTGATGCTGTTCATATAGCTATTTCTTTGGTGGATCATTCCGTTTTATCTGAAGGCTCTGGGACTGGCCACAAACTAAGTGTGATGGATTCTAATGCTGAGGCATCTAGCATGATTAGGCAATATGGGTCAATGTTTTTGCATCACGGAGATCTCCAAATGACGGTAGAGTATTATGCACAAGCTGCTGCTACTGTAGGTGGGGGACAATTAGCTTGGTCTGGAAGAAGTAATGTGGATCAACAAAGGCAGAGAAACCTAATGCTGAAGCAGCTACTGACTGAGATTCTGCTTCGAGAAAGAGGTATTTACTTTTTGCTTGGAGCAAGAGGTTCTGGTGAAGAAGGTCAGCTTGGAAGATTTTTCCCTGATTCTAGATTGAGACAACAGTTCTTGGTCGAAGCTGCACATCAATGTCAAGAAGCTGGATTATATGACAAATCCATAGAGTTACAGAAGAGGGTGGGTGCATTTTCCGCTGCCTTGGAGACTATAAACAAGTGTCTATCCGAAGCCATCTGTTCTCTTGCCCGTGGAAGGTTGGATGGTGAAAGTAGAACATCAGGGCTTATTCTCGCAGGGAACGATATTCTTGAGACTTACAAATATTACCCTGAAGTTAGTCTCCAGGAAAGAGAGCGAGTGATGGAACAAGAAACCATACTAAGAGAGCTTGAGGCAATACTATCGATCCACAAGTTGGGTAGATTAGGCAATCATCTGGATGCTTTGAGAGAAATTGCAAAACTTCCATTTCTTCATCTTGATCCAAGAATGCCCGATGCAACCGCTGATGTGTTCCAGAGCGCATCTCCTTACTTCCAGACTTGTGTCCCGGACCTGCTCAAAGTTGCTCTAACGTGTCTGGACAATGTACCTGACACTGATGGGTCGATCCGTGCCATGAGATCCAAGATTGCTGGGTTTCTTGCAAGCAACACGCACCGGAACTGGCCTCGTGACTTGTACGAGAAGGTGGCTCGAAGCTTTTGAGTATTTTTGGGCTTATCGTCAAGTCAAGTTCTCGAATCTAGTGATTCTGTTGACTTTTTGACGATTCATCTCTGGCGCATAAAAATGGGATACATTAATATACAAATTTCATGCAGCTTTTTATTAGGCCGGACACAATATCTAGGTGCCATATGTAATATTGCAGATATTGTTTCTATAGAATTTGATGTTTTGTGTGAGTTATATGATGTAGTGGATTCTGATAAGAAATTGTTATTAATTTTGGTATATCTAAGATGATATAACTTCAACTAAAATTAACTAGTTTTGTCATTTTTATTGAAAGTTTTTAATGCAAAATCGAATTTAAACAGATCCGAAGCAATTTTCATTGACGAAGATATGGTGAAACAGAGCTTTAAAAGTAGGATGGAGAAAAGGGCATGAGAAAAGAGATGAAGAAGAGAGAGATGTTCAATTCCTGCGAAATTTCCAGAAGAAGAAAACCAAACACAAAAACCCTAAACAAGATAGGCTCTTCAACAGGAGACATAACGGCCAAGTACACACAATCTCTTATAAGAACAAGCAATGTCAGTCCTTGCACAACATTTTTAACCACCAAAACTCTTTTATTTATTCCTTCTTCTTTGCATAACTTGCATATAATCCTCAAAAACAAAACACCAACCACCAAGAACGTTGTACTAGACCATCCTCGGTAAGAGACAATCTTCCCTCCCACTATTAATGGCTCTTTCCTGCATATACAAGACTTGCAGACTCCAGAGAAAGTAGATGTGACCAGAGCATCGAATCCTGTATAATAGGGGAGAATCACAGCGTCTAAGTAAAGACTAGCGTGGAGAATACCAGAGATAGTAGAGGCTTCGAAGAAGACAAAACTCGCTTCTTTTTCGATATTGTTCTCAAATAAAAGCACTGAGAGCTGAACAAGCTGTAGTATCGCAGGTCCAAAGATGGAAATTGGGAAAAGGGTATTGATCCTTTGACCTTTTGCAAGAATCAAGATGATCAAAGGAAGAGAGATTGGACCAGAAGGAAGTAAGTACCTTCTTGCTTTGTCATCATCACCATCATCATCATTTTCTCTTCTTCTTCTTGATATTACCTTCCTTACGACAACAATTAGCGTCGTCAAATAAGTAACCGTAACGAAGAAGAAAACTAGAACGTCGGTGATTTGAGGGTAGTCGCCGGCATA

1) AGGCATGCCATTTAGACCTG [ 746] AAACACCCTGTCGAACTGCT [ 897] psize 152 hbrdn 1

2) ACGAGGCATGCCATTTAGAC [ 743] AAACACCCTGTCGAACTGCT [ 897] psize 155 hbrdn 1

3) ACGAGGCATGCCATTTAGAC [ 743] ACCCTGTCGAACTGCTGAAT [ 893] psize 151 hbrdn 1

4) AGGCATGCCATTTAGACCTG [ 746] TCCTCTTCGAAACACCCTGT [ 906] psize 161 hbrdn 1

5) ACGAGGCATGCCATTTAGAC [ 743] TCCTCTTCGAAACACCCTGT [ 906] psize 164 hbrdn 1

6) GCATGCCATTTAGACCTGCT [ 748] AAACACCCTGTCGAACTGCT [ 897] psize 150 hbrdn 1

7) GCATGCCATTTAGACCTGCT [ 748] TCCTCTTCGAAACACCCTGT [ 906] psize 159 hbrdn 1

8) CCTTCGGATTCGTTTGAGAG [ 1040] GTGTTGACCGAGCAATTTCC [ 1191] psize 152 hbrdn 1

9) AGGCATGCCATTTAGACCTG [ 746] TCGAAACACCCTGTCGAACT [ 900] psize 155 hbrdn 1

10) GAACGAGGCATGCCATTTAG [ 741] AAACACCCTGTCGAACTGCT [ 897] psize 157 hbrdn 1

>gi|186507250|ref|NM_129731.5| Arabidopsis thaliana NADPH-dependent thioredoxin reductase 3 (NTRC) mRNA, complete cds

Arabidopsis thaliana NADPH-dependent thioredoxin reductase 3 (NTRC) mRNA, complete cds

GGTAAAGGTAAGGCAATCAAAAAAAATATGGCTGCGTCTCCCAAGATAGGCATCGGTATTGCCTCCGTCTCATCGCCTCACCGTGTCTCCGCCGCCTCATCCGCTCTTTCTCCTCCTCCTCATCTCTTCTTTCTCACTACTACTACTACCACACGTCACGGCGGCTCCTATCTCCTCCGTCAACCAACCCGAACTCGCTCTTCTGATTCCCTCCGCCTCAGAGTCTCCGCCACCGCCAATTCTCCGTCTTCTTCTTCTTCAGGAGGCGAGATTATCGAGAATGTAGTGATTATAGGCTCCGGTCCCGCCGGTTATACGGCGGCGATATATGCAGCGCGCGCCAATTTGAAGCCGGTGGTTTTTGAAGGGTATCAGATGGGCGGAGTTCCCGGTGGACAGTTGATGACAACTACTGAAGTTGAGAATTTTCCGGGATTCCCAGACGGTATCACGGGTCCCGATTTAATGGAGAAAATGAGAAAGCAAGCAGAGAGGTGGGGAGCAGAGTTGTATCCAGAAGATGTTGAATCTCTTAGTGTCACAACTGCTCCTTTTACTGTGCAAACTAGTGAACGTAAGGTCAAGTGCCATAGTATTATATATGCCACTGGCGCTACAGCAAGGAGGTTAAGGTTACCTCGAGAGGAAGAATTCTGGAGTAGGGGGATAAGTGCTTGTGCTATCTGTGATGGAGCTTCGCCTTTATTTAAGGGGCAAGTACTTGCCGTGGTTGGAGGAGGAGATACGGCTACAGAGGAAGCCTTGTATCTCACGAAATATGCCCGTCATGTTCATTTGCTTGTTCGCAGAGATCAGTTGAGAGCTTCCAAGGCTATGCAAGATAGAGTGATCAACAATCCAAACATCACAGTGCATTACAACACGGAAACCGTGGACGTATTGAGCAACACCAAGGGACAGATGTCTGGCATTCTACTCAGAAGACTTGATACGGGTGAAGAAACTGAGCTGGAGGCAAAAGGATTGTTTTATGGAATAGGGCATTCGCCAAACAGTCAGTTATTGGAAGGCCAAGTCGAACTCGACAGCTCCGGGTACGTCTTGGTTCGGGAAGGAACATCAAATACATCAGTTGAAGGTGTATTTGCTGCAGGAGATGTGCAGGACCACGAATGGAGACAAGCTGTAACTGCTGCTGGATCAGGATGCATAGCTGCTTTGTCAGCCGAGAGATACCTCACAAGTAACAATCTTCTTGTTGAATTTCACCAGCCTCAAACTGAAGAGGCCAAGAAAGAATTCACACAACGGGATGTCCAAGAAAAGTTTGACATCACTCTTACAAAGCACAAGGGACAGTATGCTCTTAGAAAACTATACCATGAGAGTCCAAGAGTTATATTGGTACTATACACTTCACCAACATGTGGCCCCTGTAGGACTCTGAAGCCTATTTTGAACAAGGTGGTCGATGAGTATAACCATGATGTGCATTTTGTTGAGATTGACATCGAGGAAGATCAAGAAATTGCTGAAGCAGCTGGAATTATGGGAACCCCATGTGTGCAGTTCTTCAAGAACAAGGAAATGCTCAGGACTATATCGGGTGTGAAGATGAAGAAAGAGTACCGAGAATTCATTGAGGCCAATAAATGAGGACACCAGATAGATATTGTTTCTGGCAGATGCCAAATCTTATCATGATTCAATTTTGTTCATTCAAATACTTTGGAGTTACTGGAGCAACGCCATTGAATCTCAAATGTAAACAAATAGAATCATCATCTTAAGCTGCACGCATAATGTGGTACAATTTGCTTTAAGATTTTACAAAACAACTACCCGTTTTG

1) AAACCGTGGACGTATTGAGC [ 886] CGACTTGGCCTTCCAATAAC [ 1038] psize 153 hbrdn 1

2) TTCTTCTTCAGGAGGCGAGA [ 251] TTGTCATCAACTGTCCACCG [ 408] psize 158 hbrdn 1

3) GTGATGGAGCTTCGCCTTTA [ 685] TTGCATAGCCTTGGAAGCTC [ 839] psize 155 hbrdn 1

4) TCCCAAGATAGGCATCGGTA [ 38] AATCAGAAGAGCGAGTTCGG [ 207] psize 170 hbrdn 1

5) TTCTTCTTCTTCAGGAGGCG [ 248] TTGTCATCAACTGTCCACCG [ 408] psize 161 hbrdn 1

6) CCTCAAACTGAAGAGGCCAA [ 1233] GCCACATGTTGGTGAAGTGT [ 1391] psize 159 hbrdn 1

7) TCTCACGAAATATGCCCGTC [ 767] GTCCCTTGGTGTTGCTCAAT [ 918] psize 152 hbrdn 1

8) GGAAACCGTGGACGTATTGA [ 884] CGACTTGGCCTTCCAATAAC [ 1038] psize 155 hbrdn 1

9) AGATGTGCAGGACCACGAAT [ 1115] GGACATCCCGTTGTGTGAAT [ 1278] psize 164 hbrdn 1

10) GAGCTGGAGGCAAAAGGATT [ 966] ATTCGTGGTCCTGCACATCT [ 1134] psize 169 hbrdn 1

>gi|186507259|ref|NM_129734.4| Arabidopsis thaliana camphor resistance CrcB-like protein (AT2G41705) mRNA, complete cds

Arabidopsis thaliana camphor resistance CrcB-like protein (AT2G41705) mRNA, complete cds

TTCCCGACTAAACCAACTCTACTTGTCTGTACATGTGTGTTCGACTAAACAAAGTTACAATCGTCTTCTGCTTTGTTGTTCGTCCATCTTCTACAATCGAACCTACTTCCCTTAACATCCAATAAACCAGAGAATAAATTGATGGTTTTCGATTGTCTCCTGTTAGCTCGATTTCTCCGCCGGCGACTTTTGGGATCAAATTTGATTCTTCACGATTAAGATTGAATTCGCCATTAGGTGGAGCATCATTTAAGAATCTTATGCTTTGGCTACAACGCCTATGGATACGGGTCAAAGCAGCATAGAACCTTATCAAGCCAAGTCTTTTAGTCGGGAGAGCAGTGTAGCCTCTTCTTTAAGCTTATCACGTAGTTTGCCTCACCTAATTGACAATGACGTCGATAGTGAGAGTGTCTCAGAGGCAGGGGATATTGGGGACCGCTCACTTCGGAGAAGGCATAGTGCTGGTAGAAGCAGCCGTTTGTCTGCTGATGATTTTATAGAACAAGGGACTCATGATACTTCTCGTCAAGAACAAGATATATTACATGACCTTCGAGCTTTCAACACTGCTTCCGTAAATAAAACTTTGCCTGAGGACATAACAGCATCTCCTTTACCGACCAAGTCACTCTTGTCACCTGAAATAAACAACTCCGGAAAGGAGGAAGAGCGAGTGTTACCAAAGTCCTTGGAGTACATATCATGCCTAATTCATTTGGCTGTTTTTGGGATTTTTGGGGCCATTACGAGATATTTGCTGCAAAAATTGTTTGGGCCAACTGGTGCTCGAGTAACAAGTGATGGGAGCATCTTGTACCTTGATCTTCCCTCCAACATGGTAGGATCATTCTTGATGGGTTGGTTTGGCGTTGTATTCAAAGCAGATATAGCAAGAGTTTCTGAATTTGTGGCGATAGGATTATCGACTGGTTATTTGGGGAGTCTGACAACATTCAGCGGTTGGAACCAGAAAATGCTGGATCTTAGTGCTGATGGTCAATGGGTGTATGCTGTGCTTGGCTTTTTATTAGGATTGTTTCTCACGTCATACTCCATAATTCTGGGAGTGGAAACGGCCAAAGGGTTTAAATGGCTTCTTCATAGAAGAGCTTCTTCTGAGGATAAATTCCATTGTCTTAAGGTTAACACCTTCCAGAGCCATATTGTGTCTCTGACCCTGATGCTTCTGTTGCTTGTGGCTTTACTCACTGCCAGTTCCATACTGCTTGTGAAAGAGTTTGACAAAGGAACAAGCGAGGCTCAGCTATGGTTTGGTTGCTTAGTTGCAGCCCCTGGTGTCTGGCTCAGATGGTTCTTAGCCCGACTCAATGGACGTGGGCTGGGAAAGGATAGTCAAAATTTGAGATGGGTCCCATTTGGCACCCTCATTGCAAATGTAGTTGCAGCTTGCGTTATGGCAGCATTGGCTACCTTGAAGAAATCGGTGAACACGAGAACATGTAACACGGTTGCTTCGAGCATACAGTTTGGTCTGTTGGGATGTCTGAGCACAGTTTCGACCTTCATGGCTGAGTTCAATGCGATGAGAGAAAGTGATTACCCATGGAGAGCCTATGCGTATGCATCTTTTACCATTGTGGTTTCTTTTGCGATTGGAACTATTATATACTCAGTCCCTGTCTGGGTAATAGGATTCAGTTAGTTGTATCAAAGCTAGATAAGTCTTGTGCAGATGATGTGATAATACATAGTCGTAACATTTGGTAGAGATGAATAATTAGATCGGTCACAATCAAATTATATTATACCTTGTGCAGAAATATTAAGAGCAAACTTCTATACTATAATGATAACTTTTTTCTGTTTTACACAA

1) ACCCTGATGCTTCTGTTGCT [ 1177] CCATTGAGTCGGGCTAAGAA [ 1334] psize 158 hbrdn 2

2) TGACCCTGATGCTTCTGTTG [ 1175] CCATTGAGTCGGGCTAAGAA [ 1334] psize 160 hbrdn 2

3) CCCTGATGCTTCTGTTGCTT [ 1178] CCATTGAGTCGGGCTAAGAA [ 1334] psize 157 hbrdn 2

4) TTCTTAGCCCGACTCAATGG [ 1315] ACATGTTCTCGTGTTCACCG [ 1464] psize 150 hbrdn 2

5) CCTGATGCTTCTGTTGCTTG [ 1179] CCATTGAGTCGGGCTAAGAA [ 1334] psize 156 hbrdn 2

6) ATGGTTCTTAGCCCGACTCA [ 1311] ACATGTTCTCGTGTTCACCG [ 1464] psize 154 hbrdn 2

7) GCCAAGTCTTTTAGTCGGGA [ 316] AGCACTATGCCTTCTCCGAA [ 465] psize 150 hbrdn 2

8) GATGCTTCTGTTGCTTGTGG [ 1182] CCATTGAGTCGGGCTAAGAA [ 1334] psize 153 hbrdn 2

9) GCTTCTGTTGCTTGTGGCTT [ 1185] CCATTGAGTCGGGCTAAGAA [ 1334] psize 150 hbrdn 2

10) TAGAAGCAGCCGTTTGTCTG [ 468] TTGGTCGGTAAAGGAGATGC [ 626] psize 159 hbrdn 2

>gi|186507319|ref|NM_180029.2| Arabidopsis thaliana protein kinase domain-containing protein (AT2G41930) mRNA, complete cds

Arabidopsis thaliana protein kinase domain-containing protein (AT2G41930) mRNA, complete cds

ATGTCGCCGGAGATGGAATTCGTGAAGGTTTTGGGAAAGGGTACTTACGGCTCCGTCGAGCTCTTCAGCCACAAACAAAACGACGGATCGTTGCTCTACAACGCAGTGAAGATCATGGACTCTGAGAACTACGGTTCTATAGACCAAGAGTTTCGAATTCTGTCGGAACTCAGAGGATGTCCTTGTATCGTGCAATTGTGTGGGAACTCTCTTGTTCAAGGAATTGATTGCAATGGAAAAAAAGTTTACATGATGTCAATGGAGTATGCAGCTGCTGGTACTCTAACTAATTTCATCAAAAGAAACCGAACGAAGTTGTCGGATTCTGTTATTAAAGACTTCACTCGTATGATTCTACAAGGATTGGTCTCGATTCATAATCATGGTTATGTTCATTGTGATCTTAAACCGGACAATATCCTTCTTTTTCCTCTTTACGATAAAGATACGTGGAATTGTTCTTACGAGTTGAAGATTTCGGATTTCGGAATATCGACAAGGGCCGGAGACAAATCTGGTTGTTGGAGAGTCGATGAACCGTGGGTGGGAACGTCTATCTACATGTCTCCTGAGTCAGTCAGCGACGGCACCACCGTTGAGAAAACCCTAGATTTGTGGTCACTTGGTTGCATTGTGTTGAAGATGTATACCGGTAAGCGGCCATGGTTAGGGTTTGAGAAAGATGTTAAGTCTCTTCTTTTGAATCAAAAGGCACCAGAGATTCCAGAGACTCTGCCGTGTGATGCAAGGCTGTTTTTGGAGAAGTGTTTCTCAAGAAAACCTGAGGAGAGAGGATCAGCTTCGGAGTTGCTGTTGCATCCGTTTTTGACCGGAGATGAGAAGAAGGGTTCTTCCGTCGCCGGTGGAGAGAGGACGGGGATGGTGTTGAGGCTCAGAAAACCTCCGCCGATATCGAAAGATATTCCAACGAAGCCACGGAAATTGAAGGTTATTTCACAAAAGCCCCAACAGTTAAAGAAAGTCTCGAATAAACCCCTAAAGGTGAAGATTGTTCCTCCGAGACCCCCAAGATCCGATTTTGTCCCCGTTCTATAA

1) GCCATGGTTAGGGTTTGAGA [ 659] AACGGATGCAACAGCAACTC [ 823] psize 165 hbrdn 1

2) GAGCTCTTCAGCCACAAACA [ 57] GAGTTCCCACACAATTGCAC [ 208] psize 152 hbrdn 1

3) ATCAGCTTCGGAGTTGCTGT [ 794] CCTTCAATTTCCGTGGCTTC [ 948] psize 155 hbrdn 1

4) TTCTGTCGGAACTCAGAGGA [ 157] GAATCCGACAACTTCGTTCG [ 325] psize 169 hbrdn 1

5) CCGGAGACAAATCTGGTTGT [ 502] CCGCTTACCGGTATACATCTTC [ 659] psize 158 hbrdn 1

6) TTCTGTCGGAACTCAGAGGA [ 157] CGACAACTTCGTTCGGTTTC [ 320] psize 164 hbrdn 1

7) TTCGGAATATCGACAAGGGC [ 483] CACAATGCAACCAAGTGACC [ 635] psize 153 hbrdn 1

8) CCACCGTTGAGAAAACCCTA [ 589] AAACAGCCTTGCATCACACG [ 755] psize 167 hbrdn 1

9) GCCATGGTTAGGGTTTGAGA [ 659] GATGCAACAGCAACTCCGAA [ 819] psize 161 hbrdn 1

10) TGTCGGAACTCAGAGGATGT [ 160] GAATCCGACAACTTCGTTCG [ 325] psize 166 hbrdn 1

>gi|186507330|ref|NM_129760.4| Arabidopsis thaliana uncharacterized protein (AT2G41960) mRNA, complete cds

Arabidopsis thaliana uncharacterized protein (AT2G41960) mRNA, complete cds

ATACTTAGCTGCGAATCTCGTCGTCATCGCCCCGACGAACCCTGTTTCGCGTCTCTTTCCCAATCCCAATCCAATTTCGATTCCTTCATTTCAGGATTCGTATAGCCGTAAATCCGTTATTGGGTTCTTCCGATTATCCACCGATAGATCTCGATCGAACGTTTTTCTCCGATTGGATTTTTGGGTGATTTCGCCGGAATTCATAATCAGACGGGAGGAGTCGCTTCTCTTCTCTTGAGTAATTTAGTCCCTTTTTGTGTTATGGAATCAATTGGTTGATGAACAAATACAAGTGTAAAACGATGCCGGGTTTAACAACACACATGAATGAGCATTACTCTTCTTCTGGGTTTTGGTCTGAGGACGACGATGGACTTACCTACGATCAGTTGGACCAGTTCTGGAGTGAGCTGTCGTCGAAAGCTAGGCATGAGCTTCTTAGGATTGACAAGCAGACACTGTTTGAGCAAGCTCGCAAGAACATGTGCTGTTCCAGATGCCTTGGACTGTTGCTTGAAGGCTTCGCCCAGATTCTTTCTGCTGGGAGGGCCGCGTACGAAAAAAGAATGATGGGACCCTCCAAAGATAACTGTAAATCTAATGGCACTAGGAAATGTACGGTTGCGTACCAGAGCCCTCCTGTTCATCGATGGGGAGGTCTCACCACAACACGCTCGGGATGTATCACTCTTCTTGACTGCTTTTTGACTGCAAAAACTTTCAAAGGGCTTCAAAATGTCTTTGAAAGTAACCGTGCTCGAGAGAGGGAACGTGAGTTGCTTTATCCTGATGCTTGTGGTGGCGGTGGTCGGGTATGGCTTAGTCAAGGGATTGCTGGTTTTGGTAAAGGTCATGGAACAAGAGAAACATGTAATTTGCATACCACGAGGCTCTCTTGTGATACATTAGTGGATTTTTGGTCTGCACTTGAAGAGCATAGTCGACAGTCACTTTTGAGAATGAAAGAAGAGGATTTTGTGGAAAGACTAACTTACAGATTCGACTGCAAGAAGTTTTGTAGAGACTGCCGAAGAAATGTCATTCGGGAGTTTAAAGAACTTAAGGAACTTAAACGAATACAGAGAGATCCAAGATGTACCGACTGGTTTTGTGTTGCCGATACAGCCTTTCAGTATGAGGTGGACATTGATTCTGTCCGAGCAGATTGGAGTCAATATTTTACAGAGAATGCTGGATATCATCATTTCGAGTGGGCTATTGGAACAGGAGAAGGCGAATCTGATATCCTCGAGTTCAAATATGTAGGCAATGATCGGAGTGCCCGAGTCAATGGCCTAGACCTTCGTGGATTACATGAATGCTACATTACTCTTAGGGCATTTAAAAAAAATGGCCGCCCCTCAGAAATATCTGTCAAAGCCCATGCATTGAGAGGCCAACAGTGCGTTCACTCCAGGCTTGTGGTTGGGGATGGCTTTGTTTCAATTAAAAGAGGGGAATGCATTCGAATGTTTTTTGAGCATGCTGAAGAGGCTGAGGAAGAAGAGGATGAAGTCCTGATAGACAAAGATGGAAATGAACTTGATGGTGAGTGCTTGCGTCCACAAAAGCATGCTAAGAGTCCAGAACTTGCTCGAGAGTTTCTTCTAGATGCTGCAACGGTTATTTTCAAGGAACAGGTTGAAAAGGCATTTAGGGATGGTACAGCCCGTCAAAATGCACACAGTATATTTGTCTGCCTTTCATCGGAACTGTTAGAGCAACGTGTTCATATTGCCTGCAAAGAAATTGTTACCTTGGAGAAGCAGAACAAACTTCTCGAGGAAGAAGAGAAAGAAAAGCGTGAAGAAGAGGAGCGAAAAGAAAGGAAACGAATTAAAGAAAGGGAGAAAAAACTTCGTAGAAAGGAGAGATTGAAGGAAAAAGAACGCGAAAAAGAGCAGAAGAATCCAAAATTTAGTGATAAAGCAATACTGCCAATTATGTCAAGGGAAGAAGAAGGCTCTCGAAATCTTGATGAGGACACTAACAATACCATAAGATGCGAGGAGTCCGGAATTGAAAATGGAGATGTAGATTTGTCTTCACCTGGTTCTCCTGATGATCAAGATGAAGAGTGTTTGGATGGTTGCATTTCTCCAAGAGTTGAAACCCACTCTTGTGATAGCACTGATAAGGAGATTATAGATCATGAAGATGAAAACGGCTGCTTTACACCAAGACCTGCTCATAAGACGGCGAGATTATGGAAAGAGGTTCAGACTGATCATTCTTTGAGGTTGTCAGAAAAACGCAGATTTACAGAGAAAACTTCTTTTGTCAGTAGTTCTGAGGCAGGATACTGTAATGATAGATTAGAAATGTCTTCGGGACACTTCAATGGCTCAGATAAAAATGTGAGAGTGAAAGCTTCGAAAGCTGGTGGTAGCCCAAATAGCAGTAGGTCTCATGAGGAGTTTCAGTGTTCAGATGGCAGGACTGGTGAGAGGTATGATTACCATTCTTGCAGTTGCAAACCCATTAATGGTTACCGGGAAAAGGTAGAGTCTAATACATCTGCAACCAGAGGCATGCGAGAACCGAAATCAGTATTCAAATCTGACTCTGATCTGGATGTCTCCAAGCTTAACCGAGCCAATAGGTATACTCAATCAGGCTATAGACGAGAGATAAGAAGCAAAATGAATAATAGTCGAAACGCTTGTAAGATGGATCCAGTAAATGTGAGAAAGGTCTTGGACTCTGTGGAGCCGAAGCATTCACGAAACAGCTCAACCTCTGACGTATTATCACTTACAACATACAAAGCAGAAGAGATTAAAGATGTAAGTCCCACTGTGAAGCCAGCTGGCACACCGTCTCTATGCAAGGCTACAGATAAGTTGGGAAATGGGAGTTTTAATAATTCGACGGAGGTAGACAAGAAGATGGAAGTTCACATAACACTGAAGAATGATTACCTGTACTCGAAAGATCCTATGATGAGTAGAAGTTCTAGTTCAAACAATGGAAACATCGAATCCTCCTCGATGTCTGATTCCGAAGTTGCCAGTCAGCAATCTGAAGGAAGAGAGAATCTAGTGGATACTCAGAACGATATGCCCGACTGTCACGAGAAGATGGTAGAGAAAGTGACAGAGATGAGTATGGATGAAAGAGATGTTTTAAAGATTAAGAATATATCTAACCTTCCAGCAGACAACGGGGAAAGTAAATTATCAGGAACTCCTTTTATGGTTCCTAGCCAAAACATGGAAAATATGGTACCAGGTCTTAATACTGGTTCCTACCTGTCCCAGCCACAAAACATGATACTCCCCCAGATGCTTAACCAGAGTATACCATTGCCAGTCTTCCAGGCTCCTTCAACAATGGGTTACTATCATCAAGCTCCAGTCTCTTGGTCTTCAGCTTCAACCAACGGATTAATGCAATTCCCTCATCCTAACCACTATGTATACACTGGTCCCCTTGGATACAGTCTGAATGGAGAGTCTCCTTTATGCATGCAGTACGGGACCCCATTGAACCATTCAGCAGCTCCTTTCTTCAATTCCGGGCCAGTTCCAATTTTCCATCCGTTTGCTGAAACCAACACAATGAACACTGTTGACCAAGCTCAACCTCTTGAGCCATTGGAACACAGTTTTTTGAAGGAAGCAAATGAGAGGAGATTTAACGAAATGCCTTTAATGGAAACTCCAAGGAAAAGATGTCCACAAACTGATAGTGATGAGAACTTCTCCTTGTTCCACTTTGGTGGCCCGGTTGCTCTGTCCACAGGAAGTAAAGCGAATCCTGCTCGATCCAAAGATGGGATTTTGGAGGATTTCTCGTTGCAGTTCTCAGGAGATCATGTTTTTGGTGATCCAACCGGCAATAGCAAGAAGGAGAAAGAGAACACAGTTGGTGAAGAGTACAACTTGTTTGCGACAAGTAACAGTTTGAGGTTTTCTATCTTCTAAAGTTTTTTTATACAATTTCTTGGATTGTTTGGCGTCATGGAGAACGCAGTAAAATACCTTTGGTTGTTTTATTTCATGTAAAGATCTCTTCATATTTTAAAGGTTTTGTTTTACTCAGAATGAATA

1) TGCCAGTCAGCAATCTGAAG [ 3007] TTACTTTCCCCGTTGTCTGC [ 3174] psize 168 hbrdn 1

2) TGCCAGTCAGCAATCTGAAG [ 3007] TACTTTCCCCGTTGTCTGCT [ 3173] psize 167 hbrdn 1

3) AGCTTCGAAAGCTGGTGGTA [ 2368] ATGCCTCTGGTTGCAGATGT [ 2532] psize 165 hbrdn 1

4) TTCTGGGTTTTGGTCTGAGG [ 343] AGTCCAAGGCATCTGGAACA [ 507] psize 165 hbrdn 1

5) TCTGGGTTTTGGTCTGAGGA [ 344] AGCAACAGTCCAAGGCATCT [ 513] psize 170 hbrdn 1

6) TTCTGGGTTTTGGTCTGAGG [ 343] AGGCATCTGGAACAGCACAT [ 501] psize 159 hbrdn 1

7) TTTGGTCTGAGGACGACGAT [ 351] AGCAACAGTCCAAGGCATCT [ 513] psize 163 hbrdn 1

8) GAAAGCTTCGAAAGCTGGTG [ 2365] ATGCCTCTGGTTGCAGATGT [ 2532] psize 168 hbrdn 1

9) TTGGACCAGTTCTGGAGTGA [ 389] CAGAAAGAATCTGGGCGAAG [ 539] psize 151 hbrdn 1

10) TGAGGACGACGATGGACTTA [ 358] AGCAACAGTCCAAGGCATCT [ 513] psize 156 hbrdn 1

>gi|186507338|ref|NM_129765.3| Arabidopsis thaliana phospholipase D (PLDBETA1) mRNA, complete cds

Arabidopsis thaliana phospholipase D (PLDBETA1) mRNA, complete cds

AAATCCGCGTTTCTTTGATTAATAGAAATCCGCGTTTCTCTTCTTTTCCAATTTCTTCATTGATTCTTCTTCTCGTGTCGATCAATACAATACCAAGCCAAAATCTCCATAACTCCACGCTTGCAAATTTCTTCCCAGAGGAAATAAACTCTCTAGATCAGTAGCTTATATGCGTTACTGATAATGGATAATCACGGTCCTCGTTATCCATACCCTTACGGTCAGTACCCATACCCTTACCCATACCCAGCTCCTTATAGACCTCCCAGTTCAGAGCCATACCCACCTCCTCCAACCAATCAATACAGTGCTCCTTATTACCCTTACCCACCACCTCCATACGCAACACCACCACCATATGCATCACCACCACCGCCTCATCAGCACACTTCGGGTTCGCATTCTGGACCATTAGACTATAGCCACAACCCACAACCATCATCTCTCGCGGCTGCTCCTCCAGAGTATCACAGACATTCTTTTGACTACCAACCTTCTCCTTACCCTTATCAGCCCCAAGGTAACTTTGGTGCTTATGGTCCTCCGCCTCCTCACTATTCGTATCAAGAGCCAGCTCAATACCCTCCACCTGAAACTAAACCGCAAGAGCCTCTGCCTCCTCCGCAGCAAACTCAAGGTTTTCAAGAATATCGTAGGCAAGATTGTCTCAGTACCGGGGGAACAGGTCATGATAATGTAAGCAATTCTGGATCTTCTTATCCTCCTGTGGATGAACTTCTAGGTGGTCTGCATATTTCTACTAACCAACCAGGTCCCTCAGTTCCACAACTATCCTCCCTTCCTTCGAACTCTTGGCAAAGCCGCCCTGGGGACTTGTATGGTTACCCTAACAGCTCGTTTCCAAGTAACTCTCATTTGCCACAGTTAGGGAGAGTAGATTCCTCAAGTTCTTATTATGCATCTACCGAATCGCCACATAGTGCGGATATGCAGATGACACTGTTTGGTAAAGGGTCGTTGAAGGTCCTGTTGTTGCATGGGAATTTGGATATTTGGATCTATCATGCTAAAAATCTCCCTAACATGGATATGTTCCATAAGACACTGGGTGATATGTTTGGAAGATTGCCAGGGAAAATTGAAGGGCAGCTTACTAGTAAGATTACCAGTGATCCTTATGTTTCTGTATCAGTTGCTGGTGCTGTGATTGGAAGAACGTATGTCATGAGTAACAGCGAAAATCCTGTTTGGATGCAACATTTTTATGTTCCTGTTGCTCATCATGCTGCAGAAGTTCATTTTGTTGTTAAAGACAGTGATGTTGTGGGTTCACAGCTTATTGGATTGGTTACAATCCCGGTGGAGCAGATATACTCAGGTGCCAAGATCGAAGGAACTTATCCGATTCTAAACAGTAATGGGAAGCCTTGTAAACCAGGGGCAAACTTATCATTATCGATTCAGTATACTCCTATGGACAAACTCAGCGTTTATCATCATGGAGTTGGGGCAGGTCCTGATTACCAAGGGGTACCTGGAACATATTTTCCTCTTAGAAAAGGCGGAACTGTGAGATTGTATCAAGATGCACATGTCCCAGAAGGGATGCTCCCTGGTATAAGACTGGACAATGGAATGTCTTATGAACATGGGAAGTGTTGGCATGATATGTTTGATGCCATACGTCAGGCGCGGCGTTTGATTTATATCACAGGTTGGTCAGTGTGGCACAAAGTTAAGTTGATTCGTGATAAATTAGGTCCTGCATCCGAATGTACTCTTGGGGAGCTTCTAAGATCAAAGTCCCAAGAAGGAGTGAGAGTCCTCCTTTTGATATGGGATGACCCAACTTCACGTAGCATTTTGGGTTATAAAACAGATGGGGTTATGGCAACCCATGATGAGGAAACACGCCGTTTTTTTAAGCATTCCTCAGTTCAAGTCCTGCTTTGCCCCCGAAATGCTGGAAAGCGACATAGTTGGGTCAAGCAGAGGGAAGTTGGGACAATCTATACACATCATCAAAAAAACGTAATAGTAGATGCTGATGCTGGTGGTAACAGAAGGAAAATCATAGCTTTTGTCGGTGGGCTTGATCTATGTGATGGCCGATATGACACTCCTCAGCATCCGTTGTTCAGGACACTACAGACAATTCATAAAGATGATTTTCACAACCCCACTTTTACGGGAAATCTTTCAGGATGTCCAAGAGAACCGTGGCATGACTTACACAGTAAGATTGATGGCCCGGCTGCATATGATGTTCTGACCAACTTCGAAGAGAGATGGTTGAAGGCTGCAAAGCCTAGCGGGATCAAGAAGTTTAAGACTTCCTATGACGATGCGTTGTTAAGGATTGATAGGATTCCAGATATATTAGGAGTTTCTGATACTCCTACTGTTAGTGAGAACGATCCTGAGGCTTGGCATGTTCAGATTTTCCGTTCAATCGATTCAAACTCTGTGAAAGGTTTCCCAAAGGATCCAAAAGATGCTACATGCAAGAACTTGGTGTGTGGGAAGAACGTGCTGATTGATATGAGCATACACACAGCGTATGTCAAAGCCATTCGTGCAGCCCAACACTTCATCTATATTGAGAATCAGTATTTCATTGGGTCTTCATACAACTGGAATGCGCATAAGGACATAGGTGCGAATAATTTGATTCCTATGGAAATTGCGTTGAAGATAGCTGAAAAGATCAGGGCGAATGAACGGTTTGCTGCTTATATTGTCATTCCAATGTGGCCGGAAGGTGTTCCAACCGGCGCTGCTACCCAGAGGATTCTTTATTGGCAGCACAAGACAATACAGATGATGTATGAGACAATCTACAAGGCGTTAGTGGAGACAGGGCTTGAAGGAGCATTCTCCCCACAAGATTATCTCAATTTCTTCTGTCTTGGGAACAGAGAAATGGTGGATGGAATAGATAACTCAGGAACCGGGAGTCCAAGCAATGCAAACACTCCTCAGGCGTTGAGTCGGAAAAGCAGAAGATTTATGGTCTATGTTCACTCGAAAGGGATGGTAGTTGATGATGAATACGTAGTAATTGGATCTGCAAACATAAACCAACGATCAATGGAAGGCACAAGAGATACAGAGATTGCAATGGGAGCTTACCAGCCTCAACATACATGGGCAAGGAAACATTCTGGTCCTCGTGGTCAGATCTATGGATACAGAATGTCGCTTTGGGCAGAGCATATGGCAACATTAGATGACTGTTTCACACAGCCAGAGAGTATAGAGTGTGTAAGGAAAGTGAGAACAATGGGAGAGAGGAACTGGAAACAGTTTGCAGCTGAAGAAGTCTCAGACATGAGAGGACATTTATTAAAGTATCCAGTGGAAGTTGATCGAAAAGGAAAAGTCCGACCACTTCCCGGGAGTGAGACGTTCCCGGACGTCGGAGGTAACATTGTCGGATCATTCATAGCCATACAAGAGAATCTAACCATTTGATAATAAGGAAGGATCCAGTGGCACACTTTTGACATTTTTTTTGTGTGGATATTATATCTTGAGAGTTGATAATAAGAGGGTGGGAGATTTGATTAATTTGATAAACTTTTCACATTATTAATCGTGTATAAAGTGGTGGCTTATAAAAATGAATAAAGTTGTTAATAGTTTGGCTGATATATTATCACTCTAAATTTTCCTACCA

1) TGGGAGAGAGGAACTGGAAA [ 3244] ATCCGACAATGTTACCTCCG [ 3399] psize 156 hbrdn 1

2) TTTGGTGCTTATGGTCCTCC [ 525] TTATCATGACCTGTTCCCCC [ 694] psize 170 hbrdn 1

3) TCGGTGGGCTTGATCTATGT [ 2044] TGTGTAAGTCATGCCACGGT [ 2196] psize 153 hbrdn 1

4) TGTCGGTGGGCTTGATCTAT [ 2042] TGTGTAAGTCATGCCACGGT [ 2196] psize 155 hbrdn 1

5) CGACATAGTTGGGTCAAGCA [ 1932] AACAACGGATGCTGAGGAGT [ 2098] psize 167 hbrdn 1

6) TGATGGCCGATATGACACTC [ 2063] CGGGCCATCAATCTTACTGT [ 2213] psize 151 hbrdn 1

7) TGACCCAACTTCACGTAGCA [ 1802] TGCTTGACCCAACTATGTCG [ 1951] psize 150 hbrdn 1

8) CACAACCCACAACCATCATC [ 423] TGAGCTGGCTCTTGATACGA [ 577] psize 155 hbrdn 1

9) GCAACCCATGATGAGGAAAC [ 1851] TTACCACCAGCATCAGCATC [ 2020] psize 170 hbrdn 1

10) GGCAAGGAAACATTCTGGTC [ 3110] TTTCCAGTTCCTCTCTCCCA [ 3263] psize 154 hbrdn 1

>gi|186507344|ref|NM_129766.2| Arabidopsis thaliana RING/U-box domain-containing protein (AT2G42030) mRNA, complete cds

Arabidopsis thaliana RING/U-box domain-containing protein (AT2G42030) mRNA, complete cds

GATCGGTGAAAATTTCCATTCGACGAATCGGAGTTCCAAAAGCTCGCTGACGAGTTCTCAATAGTAACCTCTCCGGCGCTTATCGTGAAAAACCCTATGTTCCAGACTTCTCTTACGCTTTCCTTGCAAAATCTGCCTCAATTTTGGATTGAAGACAAGAGCTTGAGGCTTTTTCTCCAATCTTAATTGAAACTTTGGGGTTTTTCTCACGGGGATTTTGCGAATTAGGGTTTCGGATTGGGGATTTTGTTACATCGAAAGGACTTTGGAGATTCAGCGTTGGGCTCGGTAATTGAAGAATGCAGAATTCATAAACACCGCTCTGGAATTTTACTTTTTAGGATAACAAAGGAGGGTCATTTTATCTGTTAATTGATGGGTGAAGAGATATCTAGCACTGTGAACCTGGATTTGAATCTGGGTCCTGATCCTGAGTTAGGTCTCGAGCCAGCGACGAATTATGCTCGGTCTGATTGGGGTAATGGACTTGCTGCTAGGGAATCCGAATCTCTGAGAAGGTTTAGAAGTCGGTACCGGTCACGTTTCAGACGGATTGATATGCTTCCGGTTCTTGCTGAGACACATAGCCCGGCGATGGAATTGAGTCAGTTGTTGATCACTTCTGGAAATGTAGTTGCTTTGCCTGCTGGTGAGGGAAGCATGGCTAGTGGAGAAAGGGTCATCGAGGACTCCAAAAAGTGCGAGAATGGGAGCAAAGTGATGGCAGAAGACAATGTAACAGAGGAGAAAAGAGATGTTGAGAAGAGCGTTGGCAGCGATGGGAGCTTTTTCGATTGTTATATATGTTTGGATTTGTCCAAGGATCCAGTTGTTACTAATTGTGGCCACCTTTACTGTTGGTCTTGCCTTTACCAGTGGCTACAGGTTTCAGAGGCAAAAGAATGCCCGGTTTGTAAAGGGGAGGTATCTGTGAAAACAGTGACTCCAATCTATGGACGTGGGATACAGAAAAGAGAATCTGAGGAGGTTTCAAACACCAAGATCCCTTCGAGACCCCAAGCACGACGCACAGAGAGTTTGAGGACTACACTTAACAGGTCAGGTCATATACCAACGGAAATATTTAGACATTTGCAGGATAGGCTTGAAAGGGAATCTAGTACAGGAGAAAGACGCGCCAGACCATCTCTTAACCGGTTTATGACTTCGAGGGGAGTTAGAGCAGAGGAGAACCAGTCTAGTGAAGCACTGGTTGAACCTTCATCAGATGAGATTAACGATATTGATCTGATCTTAAATAATGCCCCTGAAAATGAAGAGGAAAACGAAAACCTAAGCTCGTCACGTGCTTTGGCAACCCAGAGACGATGGGCACAGATGTATGGGAGGGTGTCGTCGTTTACTTTGAGCTCTGCTGAAAGATTAGCGGATACTTATTTGATAACACATGCATTGGGGAGGAACCAAGAACAAAACAGCAGTCCTCCTGTTGGTGTTGAAGACAGGGATTCGTTCTCAAGCATAGTAGGTGTGATAAACTCGGAGAGTCAAGTTGAAACGGCGGCTGAGATTAATTCAATGTTGACTGTATCGACTTCTTCGTCTGTTAGAAGACATGAGAACAGTTCAAGGGTCTCTGATGTTGACAGTGCAGATTCTCGTCCACTGAGGAGAAGAAGGAGATTAGCTTAGAAGAGAATGGAGGAGAAGAAAGAGGAAGCTAGAGTAATAATCTGTAGTAGAAAGCAGTGATTTTATTGGTTTTTTCAGTTTGTTTTTTATATGGGATTTTGGATTTTTGTTGTTTCAGTTTTTATGCTAAAAGACTAATAAATAAATACATCAATAGGATATA

1) CGACGAATTATGCTCGGTCT [ 451] ACTGACTCAATTCCATCGCC [ 609] psize 159 hbrdn 1

2) AATGGACTTGCTGCTAGGGA [ 480] CAGCAGGCAAAGCAACTACA [ 648] psize 169 hbrdn 1

3) ATGGACTTGCTGCTAGGGAA [ 481] CAGCAGGCAAAGCAACTACA [ 648] psize 168 hbrdn 1

4) TGGACTTGCTGCTAGGGAAT [ 482] CAGCAGGCAAAGCAACTACA [ 648] psize 167 hbrdn 1

5) GGTCACGTTTCAGACGGATT [ 535] TCGATGACCCTTTCTCCACT [ 685] psize 151 hbrdn 1

6) TCTTGCCTTTACCAGTGGCT [ 861] TCTCGAAGGGATCTTGGTGT [ 1013] psize 153 hbrdn 1

7) GGTCACGTTTCAGACGGATT [ 535] CTCGCACTTTTTGGAGTCCT [ 704] psize 170 hbrdn 1

8) TCTTGCCTTTACCAGTGGCT [ 861] CGAAGGGATCTTGGTGTTTG [ 1010] psize 150 hbrdn 1

9) CTGGATTTGAATCTGGGTCC [ 405] AATCCGTCTGAAACGTGACC [ 554] psize 150 hbrdn 1

10) GGTCATCGAGGACTCCAAAA [ 677] CTGGATCCTTGGACAAATCC [ 828] psize 152 hbrdn 1

>gi|186507351|ref|NM_129771.3| Arabidopsis thaliana DNAJ heat shock N-terminal domain-containing protein (AT2G42080) mRNA, complete cds

Arabidopsis thaliana DNAJ heat shock N-terminal domain-containing protein (AT2G42080) mRNA, complete cds

ACAAACTTTTCTCCTCCGATCTCACAGAGATGAACGCCGCCATTAGAGCAGCGATTCTCCGACCACAAAGCTATTCTTCTCAGCTCAAAACCGCGTTTTTTCATTCAACTCCTGTCTTAGAACGCAAACGTCGCACTTCCTGGGAATCGAAAGCAAATGTTCACAAGAAGAGGTTCAGAAGAATGAGGGAAAAGCAAGAGTTATTGCGTAATGTCAATGCTTTTGCTGCAAACATGTTTACGAGCTGGCATGATGAGTTTGATGACGATGGTCCCTCATCCCGGAAGCAAACCTCGTGGTTCAAAAAACAGTACTCCAAGGAACCCAAAGGAAACCAGAATAACAAACACGGCCCCTACACTTGGGGTAAAAGGAATTTTGATTTTTGCGAAGTTGATGAGGACTTTGATGTCGACTATGTATTCCGAACTGCTTTTGGAGGATCCCGAGGTTTCTCTTTTTCATTCACTCATGAAGAGGATGAACCTCGATGGCGGCATCACTCTTCAAGGTTTTCCAACAACTCTAACAGGTCTTGGAGATCGAAATATCGGTTAGATGAAGATGAAGAAGAAGAAGACTATACTTCAGACTCCAGTGACTCTGAGTCTGAGCCAAACCAAGTTTCTCATAGACAAGCACTTGGCCTGAGTCCCTCAGGTCCCTTAAACCTTAAAGATGTCAAACATGCGTATCGAACTTGTGCATTAAAATGGCATCCAGATCGTCATCAAGGCTCTACCAAGGAGGCGGCTGAAGCAAAGTTCAAGCTCTGCAGTGTGGCTTATCAATCTTTATGTGAAAAGCTATCTGTGAACTAAGAGTTAGATAGGTCTTGATTACTTAGAGATACCTCATTACAATAATTTCCGAATTGTTTCATTTTGGCTTTGTTCAAGCAAAATTGCCTTTGTTCTGTCTTTTTCTATTTCTTCAAAATCTACTACTGATGTGTAATTTATATATCTCTTCCTCTTGAACCAGTTAATT

1) CGGCATCACTCTTCAAGGTT [ 494] TCAGGCCAAGTGCTTGTCTA [ 650] psize 157 hbrdn 1

2) CAGAATAACAAACACGGCCC [ 335] CCATCGAGGTTCATCCTCTT [ 493] psize 159 hbrdn 1

3) CGGCATCACTCTTCAAGGTT [ 494] GGCCAAGTGCTTGTCTATGA [ 647] psize 154 hbrdn 1

4) CCCCTACACTTGGGGTAAAA [ 352] AACCTTGAAGAGTGATGCCG [ 513] psize 162 hbrdn 1

5) CACTTCCTGGGAATCGAAAG [ 133] TGAACCACGAGGTTTGCTTC [ 302] psize 170 hbrdn 1

6) TAGACAAGCACTTGGCCTGA [ 631] TAAGCCACACTGCAGAGCTT [ 786] psize 156 hbrdn 1

7) GTCTTAGAACGCAAACGTCG [ 113] GGGACCATCGTCATCAAACT [ 274] psize 162 hbrdn 1

8) GTCTTAGAACGCAAACGTCG [ 113] AGGGACCATCGTCATCAAAC [ 275] psize 163 hbrdn 1

9) CCTGTCTTAGAACGCAAACG [ 110] GGGACCATCGTCATCAAACT [ 274] psize 165 hbrdn 1

10) CCTGTCTTAGAACGCAAACG [ 110] AGGGACCATCGTCATCAAAC [ 275] psize 166 hbrdn 1

>gi|186507358|ref|NM_129774.3| Arabidopsis thaliana uncharacterized protein (AT2G42110) mRNA, complete cds

Arabidopsis thaliana uncharacterized protein (AT2G42110) mRNA, complete cds

TATGGATCTTGTTGTTATCACAGAGACTACTATTCCGACGAAAATCACTCACGTGAACAAGAAATCCTCCGACGAATTGCTCCGTAAGTTCGCCGATCCTGACGACGTCGACGAGTCGTCAAAGTCAACGAAACGCCGGAAGAAATCAGCTAAGAGTTCTTCTCGAGAGAAGGGCGTTGACATCGAAAGTAACACTAGTGGCTTAGTGGAGAGGAAACGGCTCTTGCTTGCCCCGGCGAGTAAACGGAGATCTCTGTTTATCCGGCAGCTTGCTTCTGGAAAATCACATCTCAGGAACAAGTCTCTCGTCAGAACCATCGGCAAGACATGGCGTAAAACAATGGAAGGAGCTTCAAGAGTTTTCATCGAGAAGCATTATAACAGACACAGGCGTCTCACCAACGACGTCGTTTAAGCTTTTTCAATCTTTTTTTCTTTTCTCACTATTTTACATTCGTTTGTATTATTCTCACGTTTAGTGTTACATCTTTCTCGTC

1) GAATTGCTCCGTAAGTTCGC [ 73] AAGCAAGAGCCGTTTCCTCT [ 228] psize 156 hbrdn 1

2) GTCAAAGTCAACGAAACGCC [ 117] AGCTGCCGGATAAACAGAGA [ 269] psize 153 hbrdn 1

3) CGACGAATTGCTCCGTAAGT [ 69] AAGCAAGAGCCGTTTCCTCT [ 228] psize 160 hbrdn 1

4) ACGAGTCGTCAAAGTCAACG [ 110] AGCTGCCGGATAAACAGAGA [ 269] psize 160 hbrdn 1

5) GTCAAAGTCAACGAAACGCC [ 117] AAGCTGCCGGATAAACAGAG [ 270] psize 154 hbrdn 1

6) CGACGAATTGCTCCGTAAGT [ 69] AGCCGTTTCCTCTCCACTAA [ 221] psize 153 hbrdn 1

7) GTCAAAGTCAACGAAACGCC [ 117] GCTGCCGGATAAACAGAGAT [ 268] psize 152 hbrdn 1

8) GAACAAGAAATCCTCCGACG [ 54] AGCCGTTTCCTCTCCACTAA [ 221] psize 168 hbrdn 1

9) GGGCGTTGACATCGAAAGTA [ 171] GTTTTACGCCATGTCTTGCC [ 338] psize 168 hbrdn 1

10) ACGAGTCGTCAAAGTCAACG [ 110] AAGCTGCCGGATAAACAGAG [ 270] psize 161 hbrdn 1

>gi|186507366|ref|NM_201939.2| Arabidopsis thaliana putative plastid-lipid-associated protein 13 (AT2G42130) mRNA, complete cds

Arabidopsis thaliana putative plastid-lipid-associated protein 13 (AT2G42130) mRNA, complete cds

TTCTTCTTCTTCTTCTTCAATGGCGTTGATACATGGTTCGGTTCCTGGTACGTCTGCGGTTCGATTGGTTTTTTCAACCTCAGCGTCACCGTCGAGATTTTGTCTCAATGTTCCGGTGGTGAAGCAGGGTTGGAAGAATTCGTGCCGGAGAAGGGTTTTAAGAGCAATGGTGCAGGAAACAGTTCAAGGATCTCCTTTGGTTTACGCCAGAGAAATGGAGCGCCTTTCTGCTAAGGAATCGCTACTTCTCGCTGTATAACTTCTCTCACTTTTATAAACTCTTTGAATTACTTGGTTGTTATGTGAATATATGATTTGTTTAGATCTTCAATTTTACTCTGATTTTGAAATTGTGTCTCTTTTACTATCTCTGTCGTAAGCTGAGGATAAATGGGAAGACTTTAGGTGAATTGTGTATTTGTTAGATGTAATCGAGATTTTGCAGATAACAAAAGAGAATTTTTTTCTCAAAGTCCAGAAATTGAATGATCATGTTTCAGCTCAATTGAGTTTTTCTTAGTATAGGAACAGAGCTCTGTGTGTTTGGAACTGTCAAACTATTTTTGTCCTGAAGCCATCTATGTACAGTTGAAGGATGCTGGAGGCTTTGAGGCTTTAGTTACAGGGAAAACAACTAATATGCAAAGGATCGATGTTAATGAGAGGATTACGAGTCTAGAGCGGCTTAATCCAACTCCGAGACCAACCACGTCTCCTTGTTTCGAAGGCAGGTGGAATTTTGAGTGGTTTGGATCTGGAAGTCCTGGTTTACTCGCTGCTCGAGTTATATTCGAGAGGTTTCCTTCAACATTGGCGAATTTATCAAGAATGGAGATACTGATCAAGGATGCTAACGCAAAGGCGACTGCAAATATCAAACTTTTGAACTCGATAGAAAGCAAAATCATTCTGTCATCAAAATTGACTGTTGAGGGACCACTGAGATTGAAAGAAGAATATGTTGAAGGTATGCTTGAAACTCCAACAGTCATTGAAGAAGCAGTTCCTGAACAGCTAAAAAGCGCTTTGGGACAAGCTGCCACAACGTTGCAGCAGCTTCCTGCTCTTATCAAGGATACTCTAGCCAGTGGTCTGAGAATTCCCCTTAGTGGATCCTTTGAAAGATTTTTCATGATATCTTATCTAGATGAAGAGATTCTTATAGTAAGAGATACCGAAGGAGTGCCTGAAGTTCTAACGAGGATAGAAACACCATCATCGACCGTTGTAGAAACTATCGAATATGATAGTTAGATGTGACTAGCCATTGTTCTTGTTCTTGTTCCTATGTTCTCTGTGTGTTATATTACAAGAACCAACCACCAAGTTGGTGTCTGTAAATGTATGACGTTAATAACTTGAGGGTCATCTCCATACATCAAAACCCCTTCCTTCTTTTGTGTTCGCTCGGCAAATCTTCTCAAGTTATCCAAGTACTTGCAGATTCTCTGTTTTGATCATCTTAATAAACTCTTTCTATGT

1) ATACCGAAGGAGTGCCTGAA [ 1171] CCAACTTGGTGGTTGGTTCT [ 1330] psize 160 hbrdn 4

2) GCGTTGATACATGGTTCGGT [ 22] GTTTCCTGCACCATTGCTCT [ 179] psize 158 hbrdn 4

3) CTGGAGGCTTTGAGGCTTTA [ 598] CCAGGACTTCCAGATCCAAA [ 766] psize 169 hbrdn 5

4) TGGAGGCTTTGAGGCTTTAG [ 599] CCAGGACTTCCAGATCCAAA [ 766] psize 168 hbrdn 5

5) TACATGGTTCGGTTCCTGGT [ 29] GTTTCCTGCACCATTGCTCT [ 179] psize 151 hbrdn 5

6) ACATGGTTCGGTTCCTGGTA [ 30] GTTTCCTGCACCATTGCTCT [ 179] psize 150 hbrdn 5

7) CTGGAGGCTTTGAGGCTTTA [ 598] GGACTTCCAGATCCAAACCA [ 763] psize 166 hbrdn 5

8) TGGAGGCTTTGAGGCTTTAG [ 599] GGACTTCCAGATCCAAACCA [ 763] psize 165 hbrdn 5

9) ATACATGGTTCGGTTCCTGG [ 28] GTTTCCTGCACCATTGCTCT [ 179] psize 152 hbrdn 5

10) CCAACCACGTCTCCTTGTTT [ 702] CCTTTGCGTTAGCATCCTTG [ 861] psize 160 hbrdn 5

>gi|186507395|ref|NM_201940.3| Arabidopsis thaliana C-CAP/cofactor C-like domain-containing protein (AT2G42230) mRNA, complete cds

Arabidopsis thaliana C-CAP/cofactor C-like domain-containing protein (AT2G42230) mRNA, complete cds

ATGTTCAAAGATCTGCAACTTCATTCATAACAGTTCTTTTTCTCTCGTTCTCTGGTTTAAAGCATAATGACGGAAGAGCTCATCGACCAATCACCACCACCTGACCCAGACCCGATTCAAAATTCGAATTTAATAATCCACCCAAGACGTGTTCCTTTCGAGCACGGCCTTCTTCCGATCCAGAAGCTCGTTTTTACCGACCCGATTCAAACTCTAGCTCCGATCAAGCAAAAGTTAGCTTCTTTGGCGACGAATCATCGCGTCGGATCTGCTGCCATTTCCGATGCACTCTCGATCTCCGATGACCATGCGCGCCTCGTTCTCGAAACTCTCTCTTCGGTGCTCCATTGCGAGACTGACCCATTGGTTTTGGCCAAACCTGAGGAAGTTGATTCCGTGGGAGCTGATTTGAGGGATCTGATATTGTTTCTCTACATTCAATCGTATAAGAAGTTATTGCCTAGGACGCATAAAGACTCTGCTTCTGTGGCTGATGTTTGGCCTTCGACTTCTGCTTTTGATGGATACTTGTCGGCATTATCTCCAATTCAGCTTGTTCGTAGCAACAGCCGTCGGTTTATGCCATCACCGACAGATGAAGAAGCTCATCAATTATCGTATCTGCAAAAGCACATTGCAAATATTATTTCTCTCCTTGCAGAGCCTGTGGAGGGACAAGGAGATGATTCTTTGGTCCTGTCTATGGAGAGTTTGGAGCACATTGGGTTTCTTGTTCATTATGGTGATAAGGGATTTGATGTACCTTCTTTAAGCCAAGCTACTCCTTTTTTTGCGAATTCTGATCCTGATATGCCCGCTGTCCCCGTTCCTGCTTCCCAAGTGAATGATTGGCTTCTCAAAGACATAGCTTCCGCCTTAGAAAGCATTTCTAACAGAATCTCTGGGAAAGAAAATGGGGCATCTAATGCCTCTGATCAGAATGCTGCAATGGCTGATTCTAGTGTAGCTCTTAATAAAGTTTCATCTAACGACAGAGGTCCATGTATTATTGAGGGAGTCTCTAAGACCTCACTTTTCAAGCAGGCTTCTGATCTTAAGGGTAGATCAGTGAAGGTTGCCAATTGCCATGATTCTGTTATTTATCTGTTAGCGCCATTGAGATATGCAACTGTGTATGGGTGTTCTGATTCTACTATCGTTCTGGGAGCTGCTGGCAAGGCAGTAAAAGTTGAGCATTGTGAGAGAGTTCATGTGATTGTAGCTACCAAACGAATTTGCATCGCCAATTGCCGTGAATGTGTGTTCTTTTTGGGAGTCAATCAGCGACCTCTTATAGTTGGTGATAACCACAAACTACAGGTTGCTCCATATAATACATTTTACTCACACTTGGAGGAGCACATAACCGAGGTAGGAATTCAGCCAACTATCAACAAATGGAACGAATCCTTGGCGCTCGGAGCAATTGATCCGCATGACTCACTATCACATCCGACTGGTGCCTCTGATAAACAAGCTGAACCGGCTTCTTGTGTGGACCCTGACCAGTTCACTACCTTTTTGATCCCAAACTGGTTTGCGGGTGAGGCGATTGGTTCCACGAAAGACAATCCATTTCCATTGCCAGATGCATATAAGGCAGTGCAGCAGACAAACCTTAAGAACTTAGAGGAAACAAGACAATCCTTGAGAGAAACACCTCTCGAAGAAAACCGAAAACGAGAACTCACTACAGCGTTCCACATGTATTTCAAAGACTGGCTATACGCGACGGGAAATATTCGGCAACTTTACTGCCTACAAGGCGATTAAGACCAATGGCGATTGAAAATGTCAGAGTCACTGTAGAGTTCTCTTGATCCGTTCCTTCTCAGAGATTTGCTAGCACGTTTGACATCAGAATTTAACTGCACAGCACGCTCTCTAACTCCTAAAGCTCTGTTTTTTTGTGTGTAGGTTTCTCTCTGCTTTTATCGTATTTATTAGTCTTAAATATCCACGTTTTTGTCCTTGAAAAACATTAAGCCATCTTGTCTTGTTGTTGATTTCTTTCAATTGTTATAACAACTTTTCTCAATACCATATATATAAATCTTTTAGTAACTA

1) CCGACCCGATTCAAACTCTA [ 196] AATGGAGCACCGAAGAGAGA [ 348] psize 153 hbrdn 2

2) CCAGAAGCTCGTTTTTACCG [ 179] AATGGAGCACCGAAGAGAGA [ 348] psize 170 hbrdn 2

3) CACCCAAGACGTGTTCCTTT [ 138] ATCGGAGATCGAGAGTGCAT [ 302] psize 165 hbrdn 2

4) GCTATACGCGACGGGAAATA [ 1721] GAGCGTGCTGTGCAGTTAAA [ 1882] psize 162 hbrdn 2

5) CGAGACTGACCCATTGGTTT [ 350] AGTCGAAGGCCAAACATCAG [ 509] psize 160 hbrdn 2

6) CGAGACTGACCCATTGGTTT [ 350] GAAGTCGAAGGCCAAACATC [ 511] psize 162 hbrdn 2

7) GAGTTTGGAGCACATTGGGT [ 707] GGCGGAAGCTATGTCTTTGA [ 875] psize 169 hbrdn 2

8) AGAGTTTGGAGCACATTGGG [ 706] GGCGGAAGCTATGTCTTTGA [ 875] psize 170 hbrdn 2

9) AAGCTCGTTTTTACCGACCC [ 183] AATGGAGCACCGAAGAGAGA [ 348] psize 166 hbrdn 2

10) GAGTTTGGAGCACATTGGGT [ 707] AGGCGGAAGCTATGTCTTTG [ 876] psize 170 hbrdn 2
